# Supplementary material for: Endogenous retroviral elements LTR8B and MER65 rewire PSG9 regulation to control trophoblast syncytialization and pre-eclampsia risk
Source: Genome Biol. 2026 Mar 9;27:73. doi: 10.1186/s13059-026-03944-z (PMC12969887; doi:10.1186/s13059-026-03944-z)

Fig. S1. Conservation LTR8B and MER65-int element in primates. UCSC browser snapshot showing the comparative genomics (GRCh38/hg38) of the PSG9 locus. The LTR8B and MER65-int are specifically present in anthropoid primates (light blue).

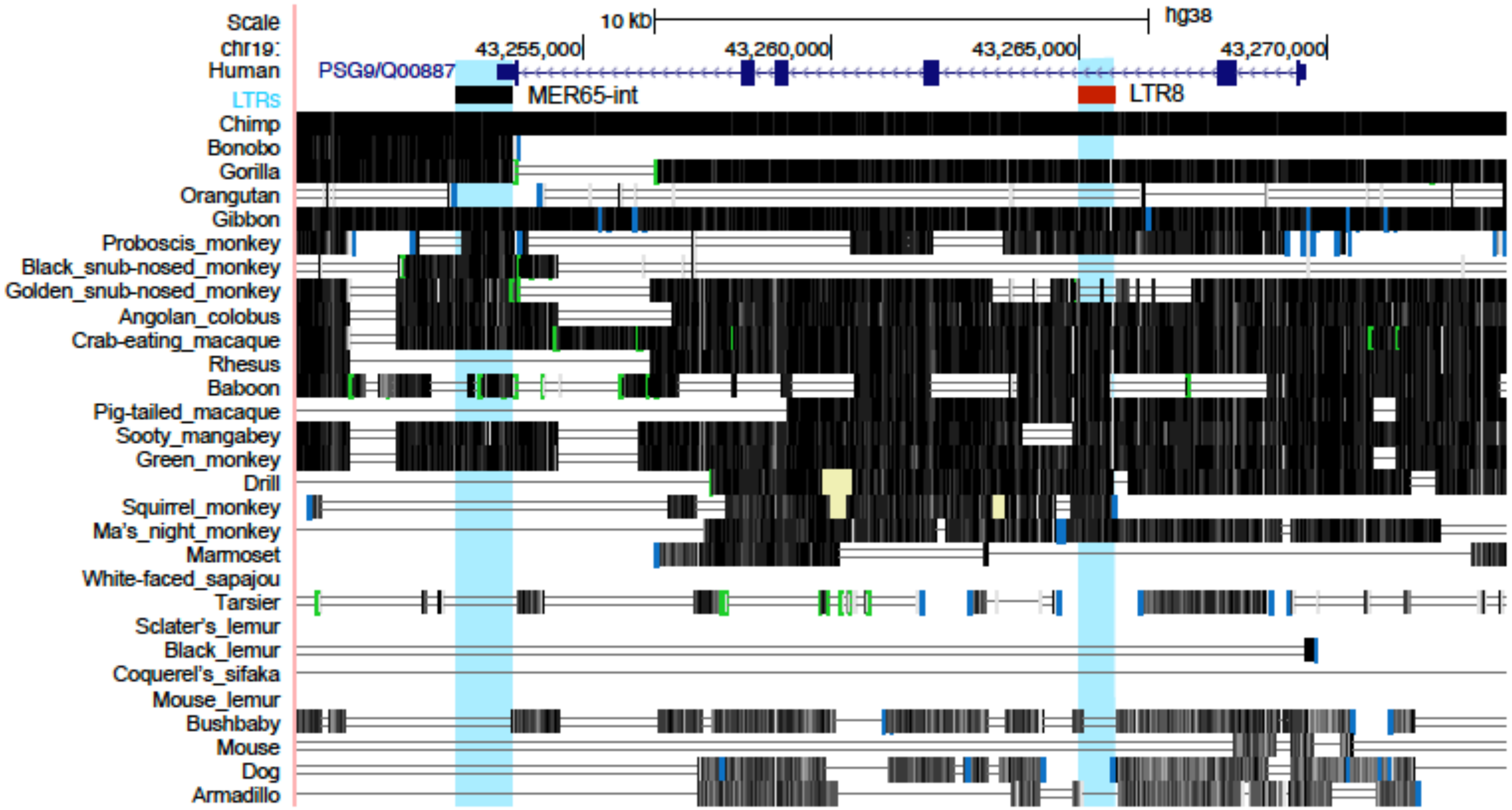

Fig. S2. Specificity of the PSG9 antibody. Specificity studies of PSG9 antibodies using Western blotting. (Left panel) Purified recombinant PSG proteins were loaded on the gels 2 µg /lane: PSG1 V5His, PSG3 V5His, PSG4 V5His, PSG5 V5His, PSG6 V5His, PSG7 V5His, PSG8 V5 His, PSG9 V5His and PSG11V5 His. Primary Ab Novus rabbit polyclonal NBP-2 19979 at a 1:1,000 dilution, followed by goat anti-rabbit HRP conjugated Ab. Note that this Ab is advertised as PSG9-specific but it is not [43]. (Right panel) 1.5 µg / lane of recombinant PSG proteins PSG1 V5His, PSG2 V5His, PSG4 V5His, PSG5 V5His, PSG6 V5His, PSG7 V5His, PSG8 V5 His, PSG9 V5His and 20 µg lysates of BeWo, SHGPL-4 cells. Primary Ab was rabbit polyclonal anti-PSG9 (ab64425) 1:1000, followed by anti-rabbit-HRP 1:5000. Please note that the band (~100kDa) most likely represents a dimer of the recombinant PSG9 protein. The PSG9 monomer is present in BeWo and SGHPL4 cells.

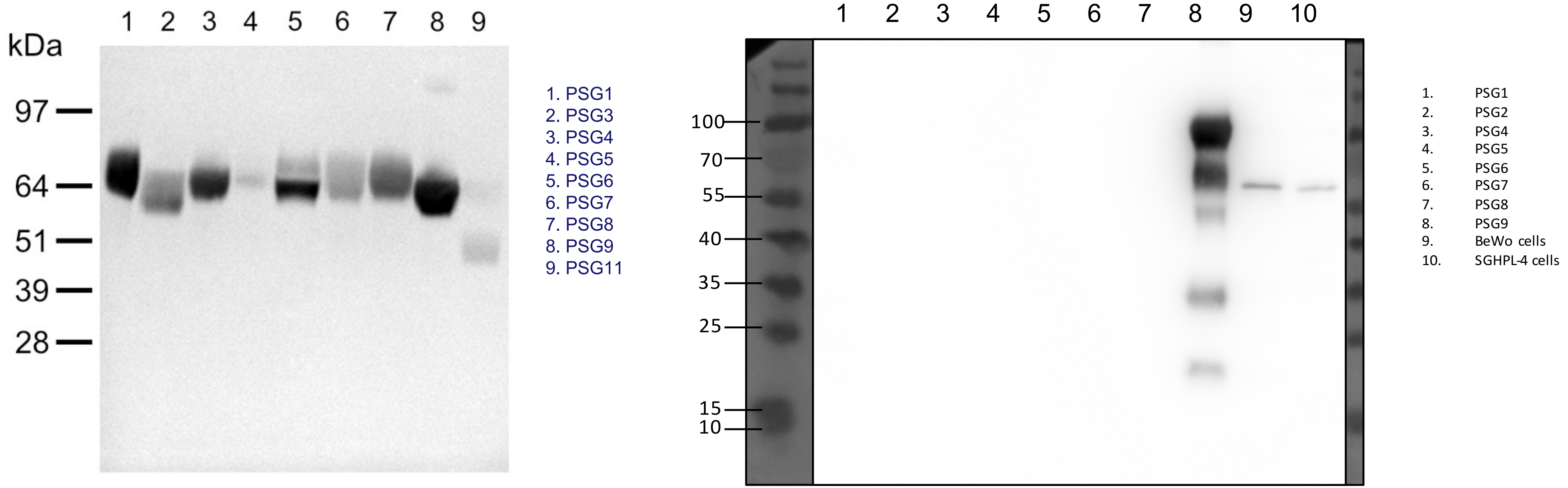

Fig S3. Endogenous retroviral (ERV) sequences identified at the PSG gene array. Note that LTR8B and MER-65-int ERVs are associated with all PSG family members, suggesting that these elements were co-amplified with the PSG genes.

	LTR8B	MER65-int	LTR16B1/B2
PSG1	X	X	no
PSG2	X	X	X
PSG3	X	X	X
PSG4	X	X	no
PSG5	X	X	X
PSG6	X	X	X
PSG7	X	X	no
PSG8	X	X	no
PSG9	X	X	X
PSG11	X	X	X

Fig. S4. **Schematic of the luciferase reporter assay to analyse ERV-derived sequences for enhancer activity.**

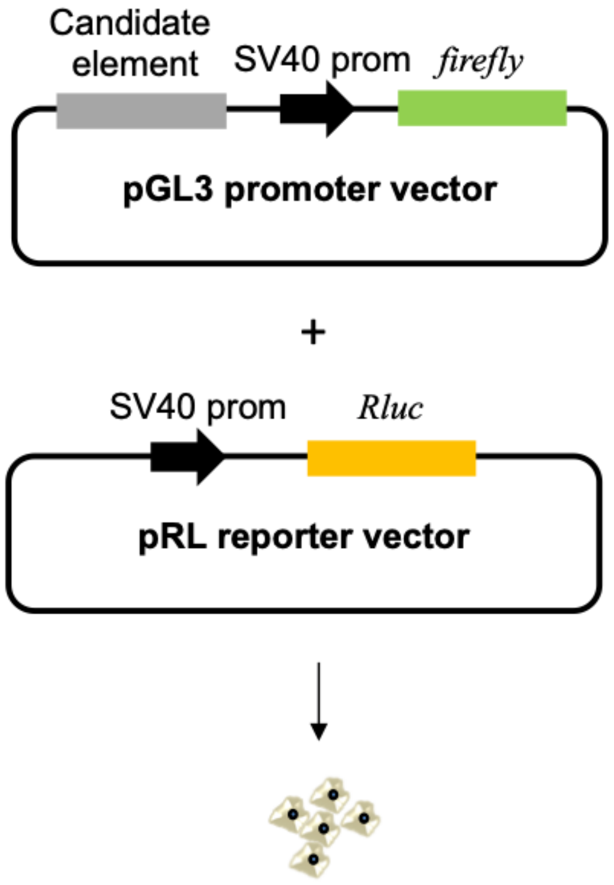

Fig S5. A Cap Analysis of Gene Expression (CAGE) of the PSG9 locus. CAGE analysis of transcription start site (TSS) usage in human trophoblast cells. Cap Analysis of Gene Expression (CAGE) was performed to map 5′-capped RNA ends at the PSG9 locus. Genomic coordinates are shown relative to the hg38 reference genome. **B** RNA-seq reads mapped to the PSG9 and the neighbouring PSG4 locus. The UCSC Genome Browser tracks show placental RNA-seq read alignments over the PSG9 (on the right) and PSG4 (on the left) loci. MER65-int elements are indicated in blue, LTR8B elements are indicated by red diamonds and gene models are indicated in navy blue. Shaded regions mark polyadenylation sites within MER65-int sequences. The read coverage patterns indicate that transcription terminates at these MER65-int-associated sites in both PSG9 and PSG4, supporting their role in alternative 3′ end usage beyond PSG9.

A

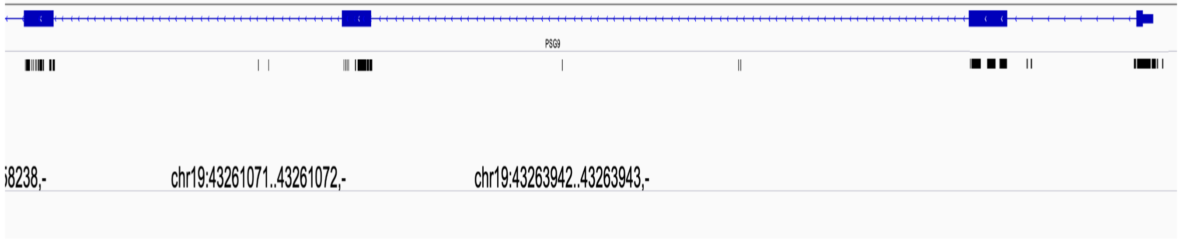

B

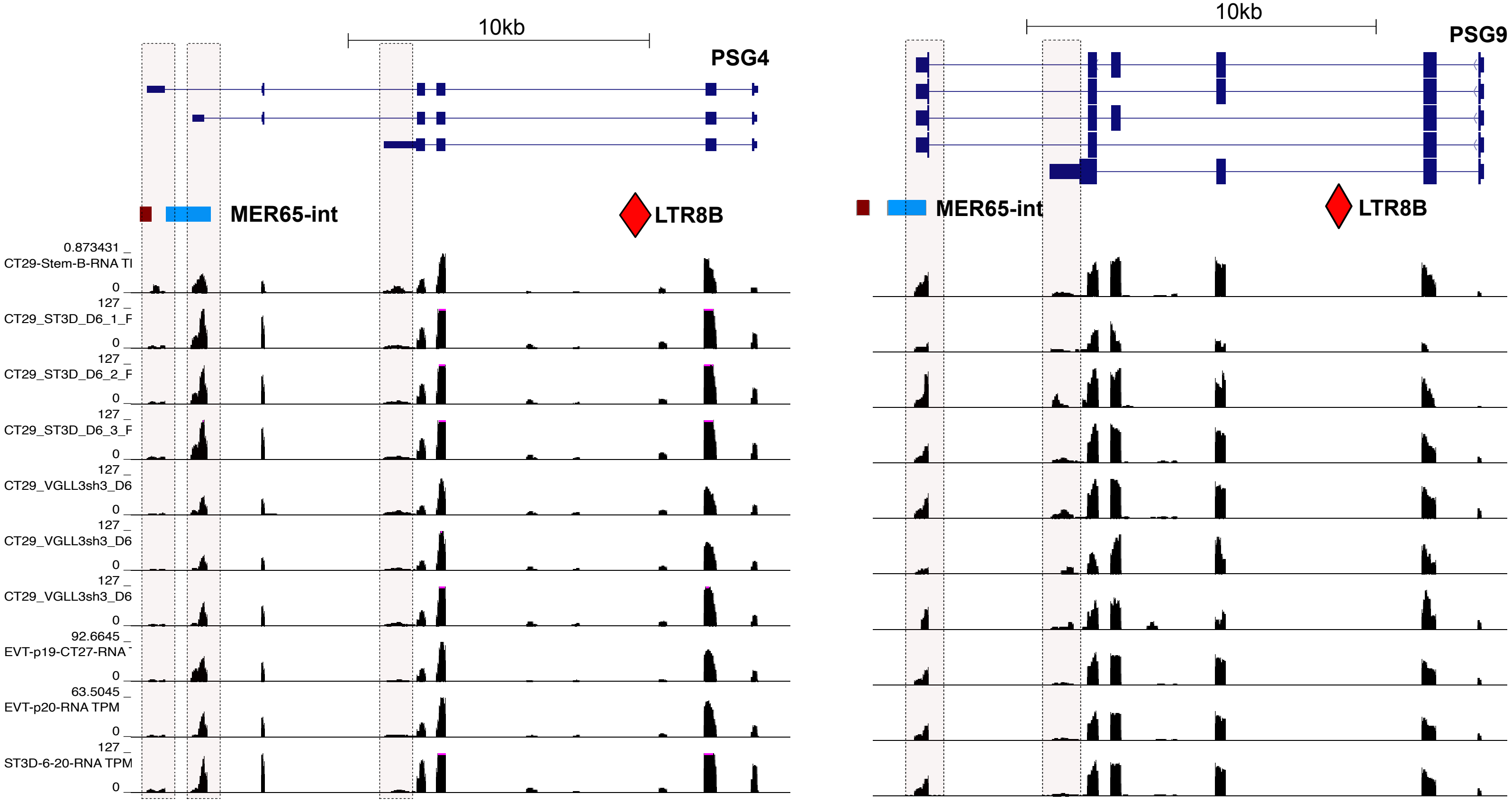

Fig. S6. Characterisation of the LTR8B/PSG9 locus. Chromatin interaction profiling of the *PSG9* gene region (Hg38: Chr19:41,265,378-45,265,378). The upper panel shows the arc-diagram of the identified interactions with the *P*-value < -log10 (2). The lower panel shows the genes in the interaction range.

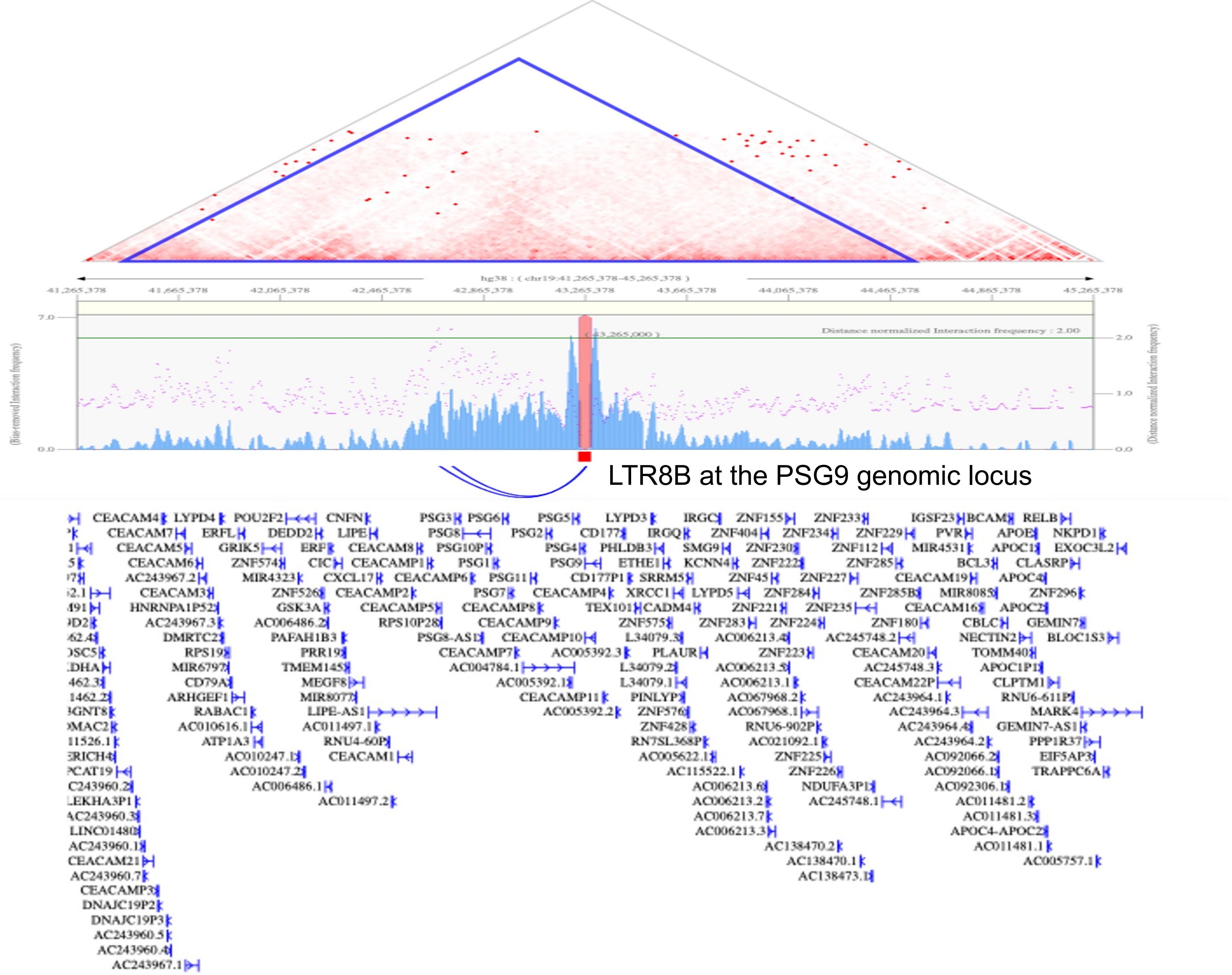

Fig. S7. Characterisation of the LTR8B/PSG9 locus using promoter capture profiling–Hi-C (PC-Hi-C). Genome browser snapshot showing the chromatin features of the genomic region overlapping with the LTR8B in the PSG9 2nd intron in human PSC-derived trophoblasts (PC-Hi-C analysis). One-to-all interaction frequency. Blue bar graph represents bias-removed chromatin interaction frequency, and magenta dots represent distance-normalized interaction frequency. Arc-diagram shows the identified interactions with the distance normalized interaction frequency >2, interaction range = 2Mb. (Bottom panel) shows the genes among the interaction range. The PC Hi-C shows the interaction of *PSG9* promoter/enhancer with *PSG4* promoter and other genomic regions (GRCh37/hg19). The arc-diagram shows the identified interactions with the *P*-value < -log10 (2), interaction range = 1Mb.

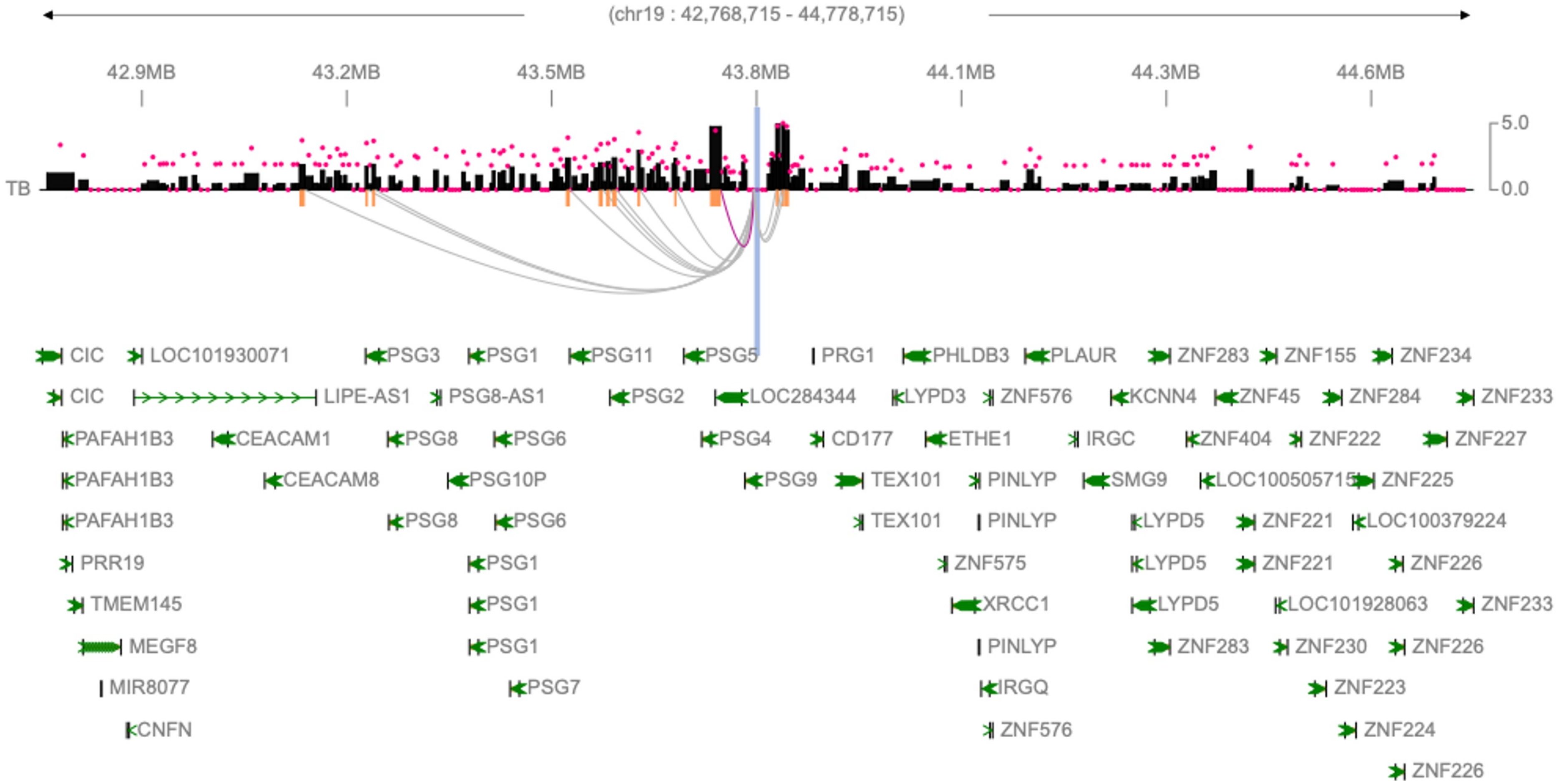

Fig. S8. Knocking out LTR8B at the PSG9 locus (KO). A (Upper panel) The CRISPR/Cas9-mediated deletion of LTR8B/PSG9 (990bp) using the guide RNA pair, shRNA1 and sgRNA3. Confirmed by genotyping and Sanger sequencing. **(Lower panel)** The CRISPR/Cas9-mediated deletion of LTR8B/PSG9 (690bp) using the guide RNA pair, shRNA2 and sgRNA3. Confirmed by genotyping and Sanger sequencing (Lower panel). **B** Downregulation of selected STB genes upon KO-LTR8B. **C** Downregulation of selected genes, involved in STB differentiation. **D** Deletion of LTR8B/PSG9 results in increased levels of CDX2 and TEAD4. **E** Overexpression of PSG9 (both membrane-bound and secreted PSG9 isoforms (PSG9-201, PSG9-202 and both together) does not rescue the expression of STB marker genes in a LTR8B KO background (P6-B4). N = 3 independent replicates, mean ± SD. Unpaired t test. Control vs. isoform-2 for *CGB* expression, ** P = 0.001; control vs. each sample in the rest, not significant (ns).

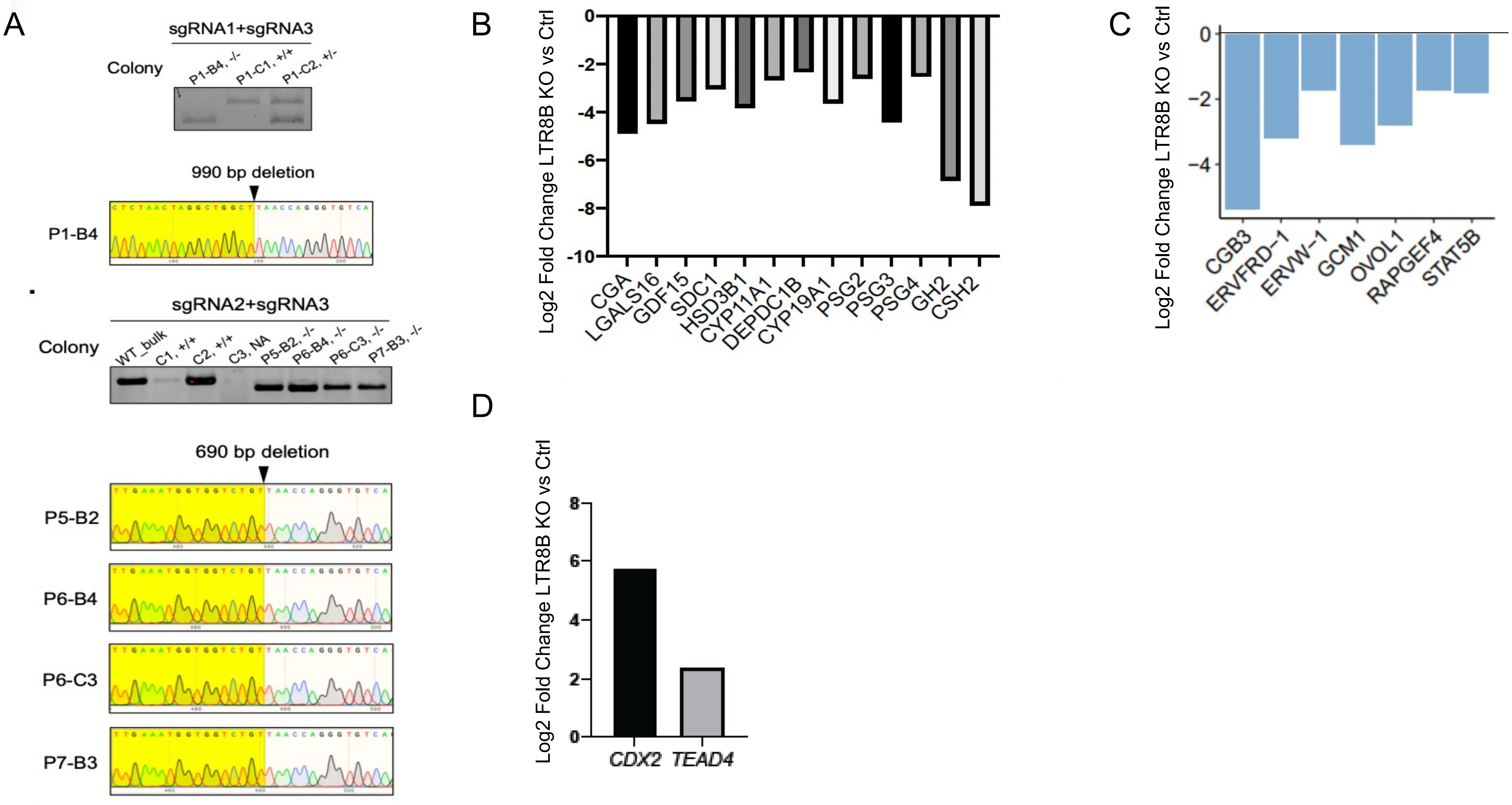

Fig S9. RNA-seq read alignment across the PSG locus revealed that CRISPR–Cas9-mediated deletion of LTR8B at the PSG9 locus abolished transcription of PSG9, while other members of the PSG family retained detectable transcript levels. Although expression of these PSGs was reduced, reads mapped across their exons. This suggests that the LTR8B element at the PSG9 locus primarily acts as a local enhancer for neighbouring PSGs, rather than off-target editing effects being responsible for the reduction**.**

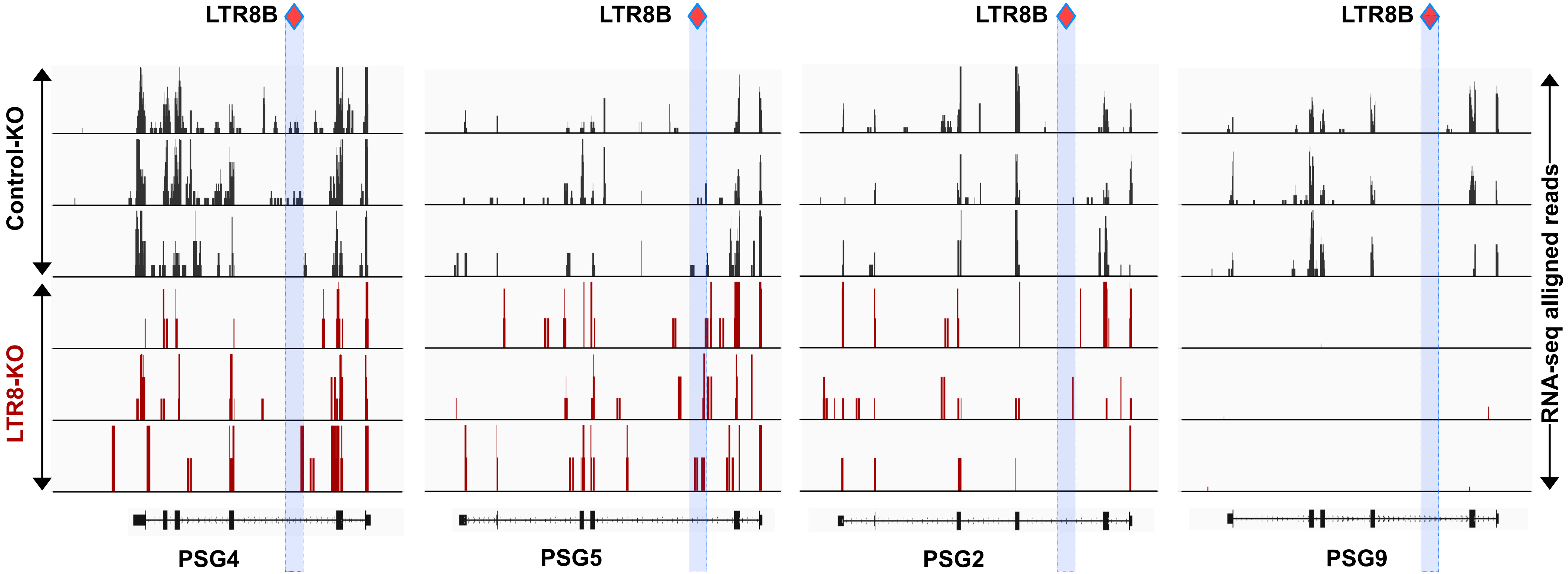

Fig S10. The rescue assay of LTR8B deletion by overexpression of PSG9 isoforms 201, 202 and 201_202. N = 3 independent replicates, mean ± SD. Unpaired t test. Control vs. PSG9-201 for *CGB* expression, ** $P = 0.001$; control vs. each sample in the rest, not significant (ns).

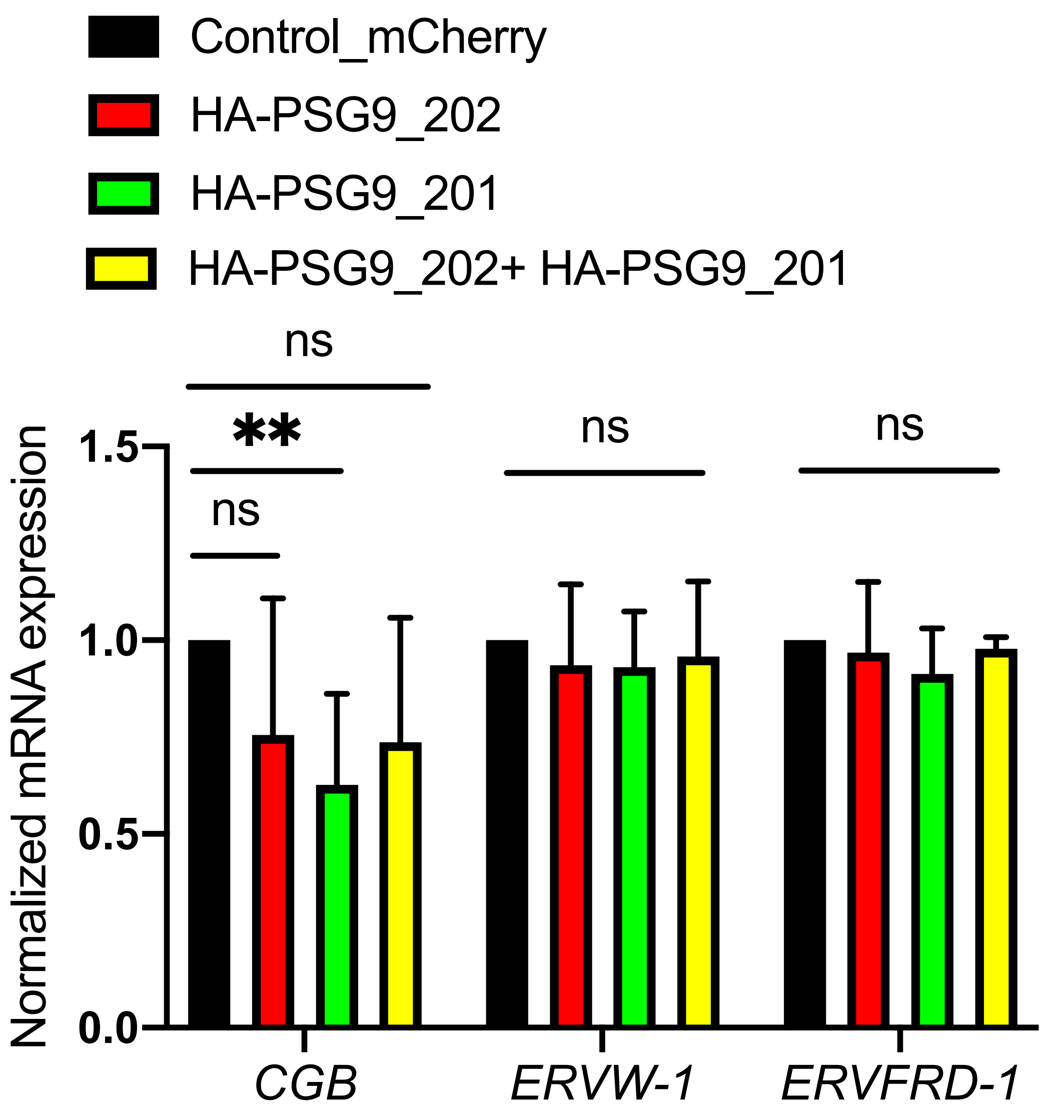

Fig. S11. Impact of the ectopic expression of trophoblast specific TFs on PSG9/LTR8B. A (Top Panel) Validation of the ectopic over-expression (OE) of HA-tagged TFs (HA-GATA3 and HA-TFAP2A) by Western blotting (representative image of three independent replicates). **A (Bottom Panel)** RT-qPCR analysis on the overdosing effect of GATA3 and TFAP2A TFs on the reporter gene expression in STBs. N = 8 technical replicates, mean ± SD. Ordinary one-way ANOVA followed by multiple comparation. Control vs. GATA3 or TFAP2A, **** $P < 0.0001$. **B** Validation of the ectopic over-expression (OE) of HA-tagged TFs (HA-GATA2, HA-GATA3, HA-TFAP2A, HA-TFAP2C). **C** RT-qPCR analysis on the overdosing effect of various TFs on the expression of PSG1 in STBs. N = 3 independent replicates, mean ± SD. One-way ANOVA followed by multiple comparation. The measured differences were not significant (level $P < 0.05$).

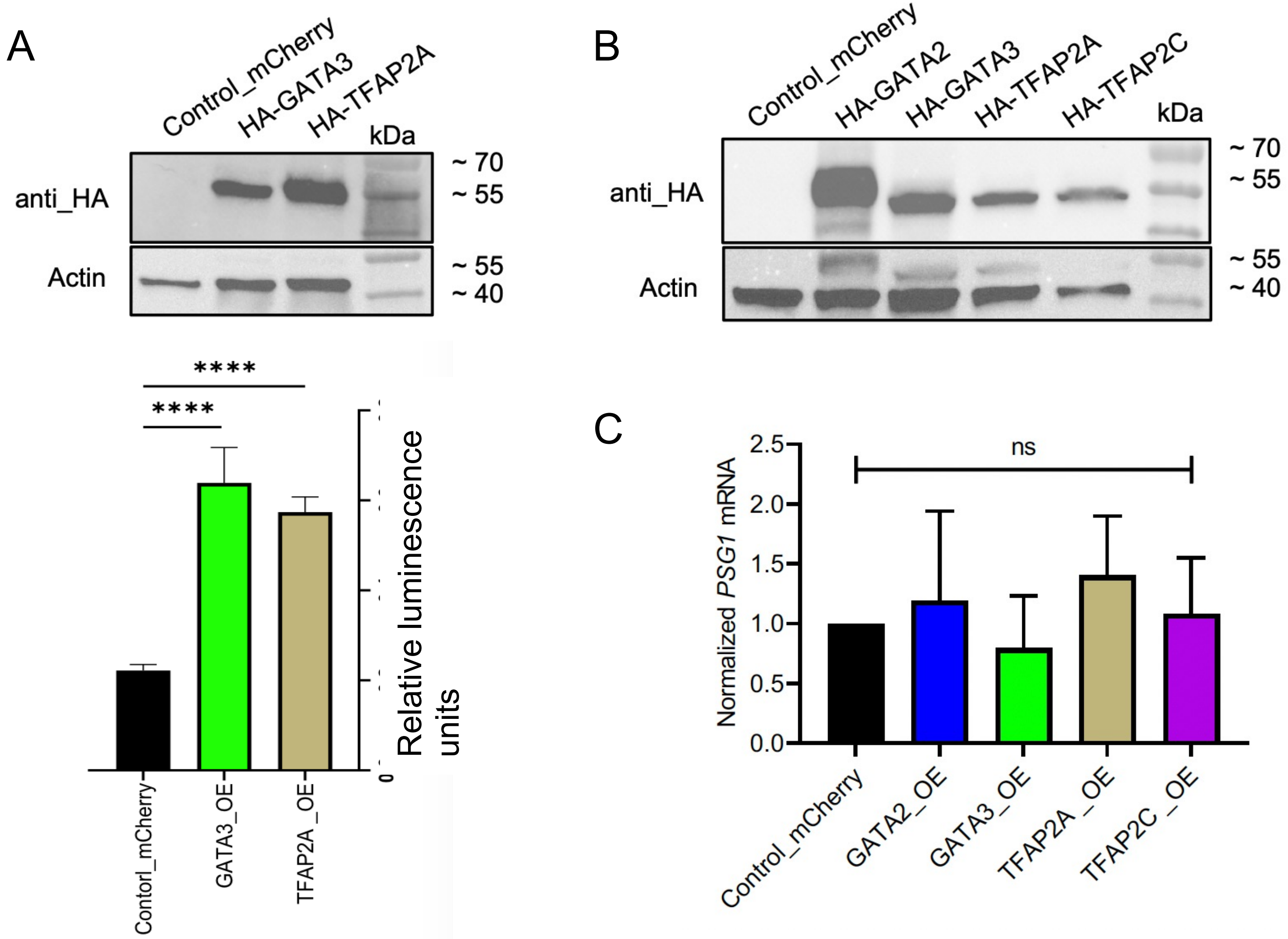

Fig S12. DLX5 is present in the regulome of GATA3 and is downregulated inDLX5 depleted TSCs. A TF–gene regulons were inferred from single-nucleus RNA-seq (snRNA-seq) trophoblast datasets using SCENIC. Yellow circles represent GATA2 and GATA3 hubs. Squares denote TFs: blue = GATA2-specific regulons, red = GATA3-specific regulons, green = TFs shared by both GATA2 and GATA3. Grey circles represent non-TF target genes. Solid lines indicate predicted positive regulation; dashed lines indicate predicted negative regulation. The networks highlight distinct TF neighborhoods for each factor, with limited overlap (e.g., ARID3A, ATF4). GATA2 is connected to TFs/cofactors such as CEBPB, JUN/FOSL2 (AP-1), ATF3, DLX3/4, SNAI1, and WWTR1/TAZ, whereas GATA3 is connected to DLX5, RBPJ (Notch), ARID5B, BHLHE41, HMGA1/HMGN2, JUND, and others. **B** The transcript levels of PSG9 are decreased upon DLX5 knockdown (KD).

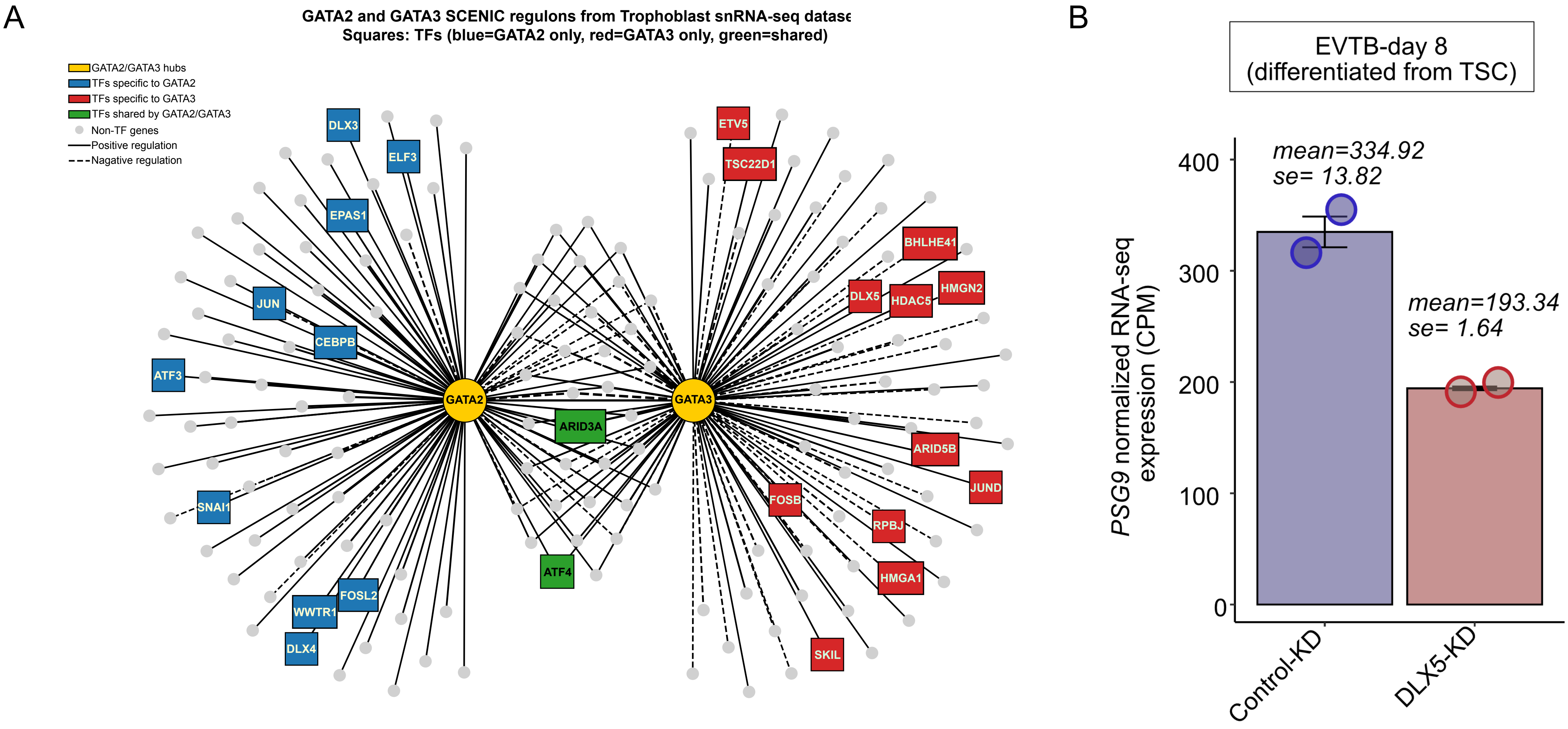

Fig. S13. A Annotated PSG9 isoforms. The consensus polyA sequence is embedded in the MER65-int element at the PSG9 locus. **B** Prediction of polyA signals in the transcripts of PSG family members using PolyApred. Prediction results based on A[A/U]UAAA* PAS signature sequence.

A

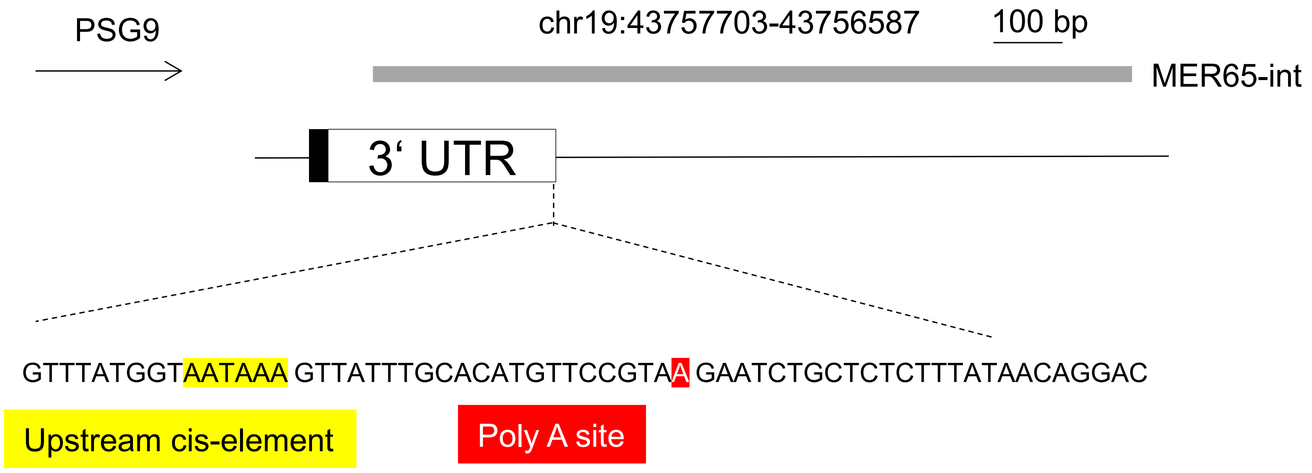

B

PSG	MER65-int GRCh38/hg38	PSG transcript	PSG transcript coordinates	prediction	PolyA motif	form the end (bp)
PSG1	chr19:42866757-42867953	ENST00000436291.7	chr19:42,866,464-42,879,713	Positive	AATAAA	18
PSG2	chr19:43063357-43064840	ENST00000406487.6	chr19:43,064,209-43,082,701	Positive	AATAAA	18
PSG3	chr19:42720790-42722258	ENST00000595140.5	chr19:42,721,642-42,729975	Positive	AATAAA	18
PSG4	chr19:43192995-43194192	ENST00000405312.8	chr19:43,192,702-43,205,638	Positive	AATAAA	20
PSG5	chr19:43166888-43168375	ENST00000342951.11	chr19:43,167,743-43,186,536	Positive	AATAAA	18
PSG6	chr19:42901229-42902351	ENST00000187910.7	chr19:42,902,086-42,917,894	Positive	AATAAA	12
PSG7	chr19:42924426-42925625	ENST00000406070.7	chr19:42,924,132-42,937,207	Positive	AATAAA	20
PSG8	chr19:42752983-42754185	NM_001130167.2	chr19:42,752686-42,7656778	Positive	AATAAA	23
PSG9	chr19:43006801-43008272	ENST00000320078.12	chr19:43,007,656-43,026,469	Positive	AATAAA	18
PSG9	chr19:43235514-43237011	ENST00000418820.6	chr19:43,211,791-43,269,530	Negative	-	N/A
PSG11	chr19:43006801-43008272	NM_001113410.2	chr19:43,007,656-43,026,469	Positive	AATAAA	18

Fig. S14. MER65 defines subcellular localization of PSG9. A Schematic of the luciferase reporter assay to validate the polyA signal embedded in MER65-int. **B** Hydrophobicity analysis of the PSG9-202 and PSG9-201 isoforms. Note: **C** Transmembrane prediction of the PSG9-202 and PSG9-201 isoforms (TMHMM probabilities).

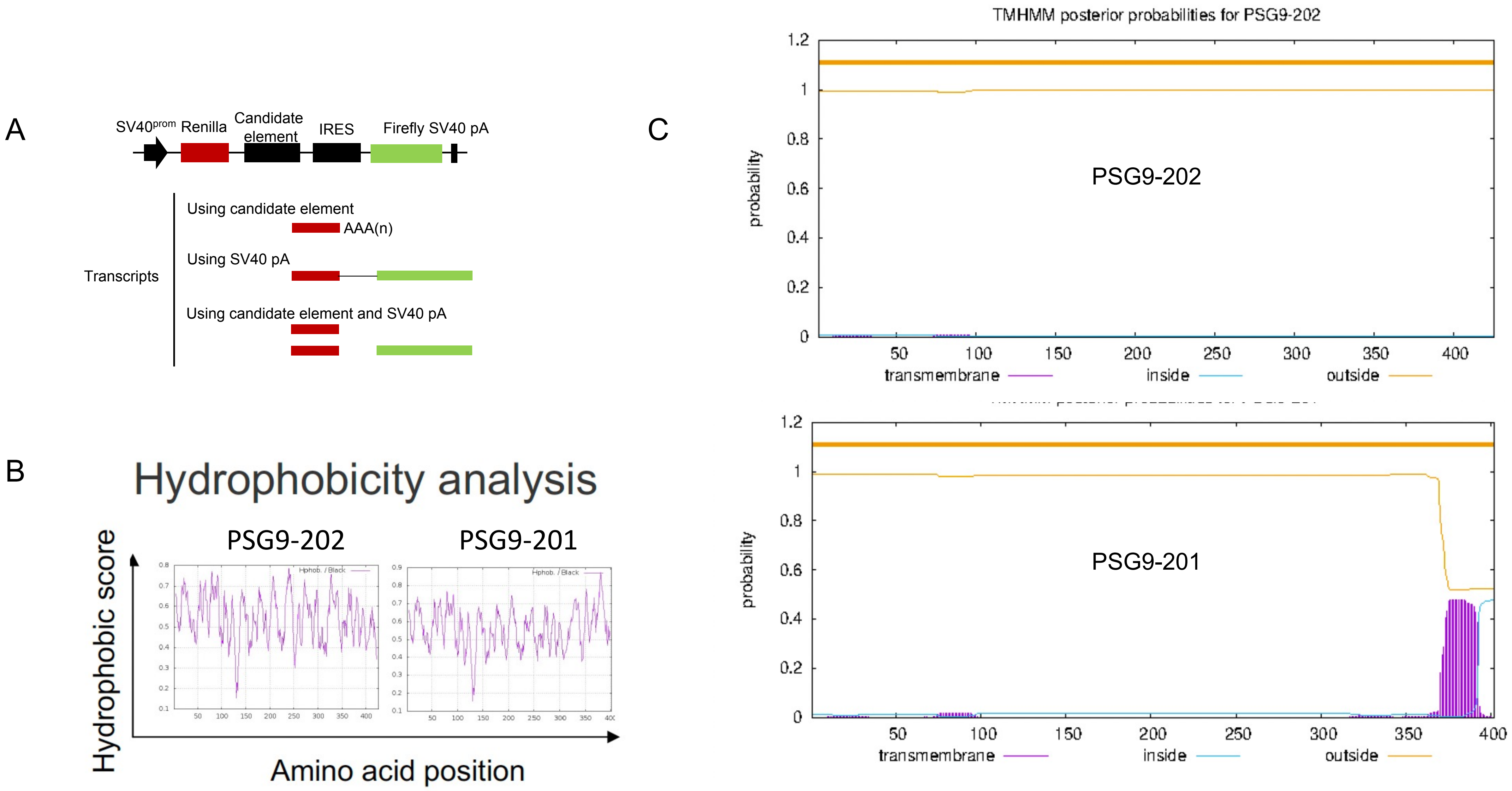

Fig. S15 Characterisation of the membrane bound and secreted PSG9 isoforms. A Pairwise comparison of PSG9-201 and PSG9-202 amino acid sequences. Note that PSG9-201 lacks the A2 domain but contains a predicted transmembrane region. **B** The strategy of marking PSG9-201 and PSG9-202 protein variants with HA and FLAG tags, respectively, and stably coexpress them in BeWo cells with the help of the *Sleeping Beauty* transposon system.

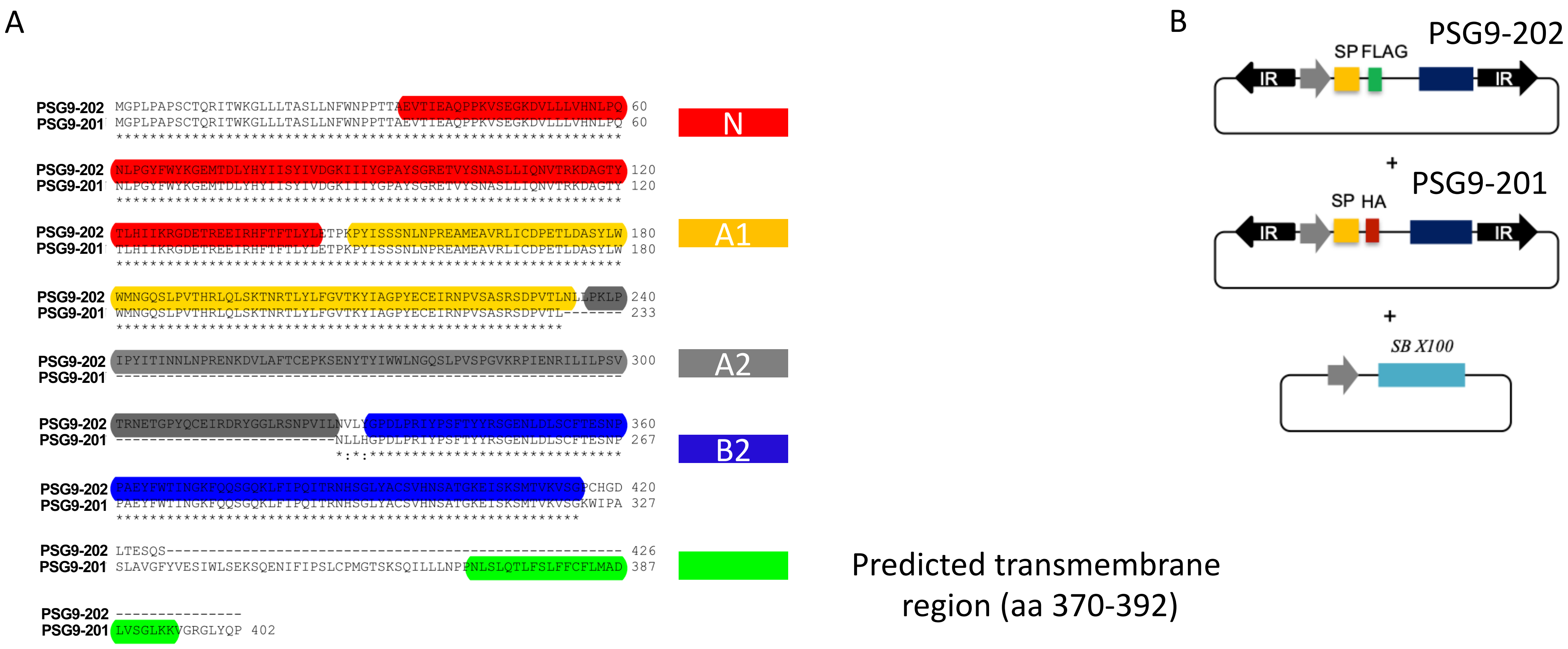

Supplement: Supplementary file 2 — Additional file 2. Supplementary Figures S1-S9 and uncropped images. [file 13059_2026_3944_MOESM2_ESM.pdf]
