# Supplementary material for: Endogenous retroviral elements LTR8B and MER65 rewire PSG9 regulation to control trophoblast syncytialization and pre-eclampsia risk
Source: Genome Biol. 2026 Mar 9;27:73. doi: 10.1186/s13059-026-03944-z (PMC12969887; doi:10.1186/s13059-026-03944-z)
Supplement: Supplementary file 3 — Additional file 3. Patients and clinical data. [file 13059_2026_3944_MOESM3_ESM.pdf]

Patients and clinical data

PE was defined as women with the new onset of hypertension (systolic blood pressure of at least 140 mmHg or more and/or diastolic blood pressure of at least 90 mmHg) after 20-week gestation, accompanied by proteinuria (≥ 0.3 g protein in a 24-hour urine specimen). Pregnant women with multiple gestations, smoking history or chronic diseases (e.g., diabetes, thyroid disease, kidney dysfunction, autologous Immune diseases) were excluded from the current study. Early-onset PE is defined as delivery < 34 gestational weeks. Late-onset PE is defined as delivery ≥ 34 gestational weeks. BMI: body mass index.

The following cohorts were used in this study:

Oslo cohort: The placental samples are comprising of patient samples from bio-bank collection at Oslo University Hospital, Norway. The Oslo cohort consists of placental tissues collected during elective Cesarean sections in 24 early onset (EO) PE (delivery < 34 gestational weeks) and 27 controls with normotensive and uncomplicated pregnancies. The uncomplicated pregnancy group consisted of healthy, normotensive women undergoing elective Cesarean section due to breech presentation or other reasons. The Oslo Pregnancy Biobank samples stem from an ongoing recruitment of pregnant patients. The study (and the present research collaboration) is approved by the Regional Committee of Medical Research Ethics South Eastern Norway (ref: 2013/2092 and 529-02162). Informed written consent was obtained from each participant.

**Oslo Characteristics at delivery**	**Controls (n=27)**	**EO-PE (n=24)**	**Statistics**
**Maternal age (Years)**	31.2 ± 4.2	31.6 ± 5.6	P=0.63
**BMI (kg/m^2)**	28.6± 3.4	31.5 ± 5.2	**P ≤ 0.01
**Gestational week**	39.0 ± 0.9	33.7 ± 3.7	***P ≤ 0.001
**Systolic BP (mm Hg)**	119 ± 11.6	165 ± 16.2	***P ≤ 0.001
**Diastolic BP (mm Hg)**	72.1 ± 10.9	101.1 ± 6.7	***P ≤ 0.001
**Baby weight (g)**	3492± 402	2170 ± 1005	***P ≤ 0.001

Charité-Aachen cohort:

Preeclamptic and IUGR term placenta samples and gestational age matched controls were collected in the University Hospital of the RWTH Aachen, Germany (as described above) and at Charité – Universitätsmedizin Berlin. Sampling was approved by the local ethical committees (Aachen: EK 148/07; Berlin: EA2/132/12) and informed consent was obtained from each participating woman. Clinical characteristics of the PE, IUGR and control cases from the study population are listed in the table.

Charite/Aachen Characteristics at delivery	Control early (n=36)	Control late (n=65)	IUGR early (n=28)	IUGR late (n=13)	PE Early onset (n=14)	PE late onset (n=23)
**Maternal age (Y)**	31.4 ± 5.7	32.5 ± 5.6	27.7 ± 6.4	27.6 ± 5.8*	33.0 ± 5.3	31.2 ± 6.2
**BMI (kg/m^2)**	25.0 ± 6.1	23.6 ± 4.6	24.0 ± 4.8	26.2 ± 7.0	26.0 ± 5.1	27. ± 7.0*
**Gestational days**	203.3 ± 22.1	264.7 ± 13.0	208.6 ± 19.0	264.3 ± 11.8	206.1 ± 21.3	254.5 ± 12.0
**Systolic BP (mm Hg)**	115 ± 9.7	116.6 ± 10.8	122.9 ± 13.7***	104.5 ± 15.5*	152.6 ± 12.4***	153.5 ± 14.5***
**Diastolic BP (mm Hg)**	65.0 ± 8.0	69.7 ± 8.1	70.7 ± 11.7***	65.2 ± 10.3	95.9 ± 8.7***	94.8 ± 8.8***

Data are presented as mean ± standard deviation. *P ≤ 0.05, ***P ≤ 0.001 vs. control.

***Charité*-Berlin cohort:** In addition to the placenta samples collected from patients in doi: 10.1161/CIRCULATIONAHA.117.028110, a further three control and five EO-PE placentae were obtained from HELIOS Klinikum Berlin-Buch with the approval of the Regional Committee of the Medical Faculty of Charité Berlin (making a total of ten EO-PE and eight healthy placentae). EO-PE patients were defined as having hypertension (systolic blood pressure (SBP) ≥140 mmHg or diastolic blood pressure (DBP) ≥90 mmHg) and proteinuria (≥0.3 g in a 24-hour urine specimen) before the 34(th) week of pregnancy. The gestational age was 30.5 ± 4.5 weeks. Control patients were defined as having had an uncomplicated term pregnancy. All samples were collected and processed within two hours of Caesarean section delivery.

Manchester Antenatal Vascular Service Cohort:

Maternal plasma samples included in this study were collected from 24th - 28th weeks of gestation from a high-risk cohort in the United Kingdom, the Manchester Antenatal Vascular Service (The MAViS clinic). Informed written consent was collected from each participant. The study was approved by the NRES (National Research Ethics Service) Committee North West 11/NW/0426. The

inclusion criteria for women in the MAViS study were: (i) chronic hypertension BP ≥140/90 at ≤20 weeks; (ii) chronic hypertension requiring antihypertensive treatment ≤20 weeks; (iii) pre-gestational diabetes mellitus with evidence of vascular complications (hypertension, nephropathy); (iv) history of ischaemic heart disease; and (v) previous early onset preeclampsia.

Hungary cohort:

Serum samples were obtained from women with uneventful pregnancies (healthy controls) and women with preeclampsia at the First Department of Obstetrics and Gynaecology, Semmelweis University, Budapest, Hungary. The study was approved by the Regional and Institutional Committee of Science and Research Ethics at Semmelweis University (IRB No. 188/2008), and written informed consent was obtained from all participants. The research was conducted in accordance with the principles of the Declaration of Helsinki. All participants were Caucasian and resided in the same geographic region of Hungary. The inclusion criteria required women to be at least 18 years old and to have a singleton pregnancy. Exclusion criteria included multifetal gestation, pre-existing chronic hypertension, diabetes mellitus, autoimmune diseases, angiopathies, renal disorders, maternal or fetal infections, or fetal congenital anomalies. Preeclampsia was diagnosed according to the following criteria: systolic blood pressure ≥140 mmHg or diastolic blood pressure ≥90 mmHg on at least two occasions, separated by ≥6 hours, after 20 weeks of gestation in a previously normotensive woman, in conjunction with proteinuria (≥0.3 g/24 hours or ≥1+ on a urine dipstick test, excluding urinary tract infections). Clinical characteristics of the study groups are summarized in the Table below.

**Hungary Cohort Characteristics**	**Controls (n=92)**	**PE (n=67)**	**Statistics**
**Maternal age (Years)**	31.4 ± 3.9	31.2 ± 4.5	ns
**BMI (kg/m^2)**	27.4± 4.3	29.7 ± 3.4	*P ≤ 0.05
**Gestational week**	36.0 ± 0.8	35.5 ± 0.7	ns
**Systolic BP (mm Hg)**	120 ± 4.5	150 ± 10.1	***P ≤ 0.001
**Diastolic BP (mm Hg)**	70.2 ± 9.3	100.9 ± 8.6	***P ≤ 0.001
**Baby weight (g)**	3415 ± 480	2575 ± 1210	***P ≤ 0.001
