# Supplementary material for: Endogenous retroviral elements LTR8B and MER65 rewire PSG9 regulation to control trophoblast syncytialization and pre-eclampsia risk
Source: Genome Biol. 2026 Mar 9;27:73. doi: 10.1186/s13059-026-03944-z (PMC12969887; doi:10.1186/s13059-026-03944-z)
Supplement: Supplementary file 4 — Additional file 4. RNA-Sequencing quality metrics. [file 13059_2026_3944_MOESM4_ESM.pdf]

Quality control of RNA-sequencing

1. **Edited trophoblast lines**

1.1. Quality control of raw reads

The reads quality was examined using 'FastQC' and 'MultiQC' {Ewels, 2016 #510} with the scripts following http://www.bioinformatics.babraham.ac.uk/projects/fastqc/.

fastqc path/to/sample.fq.gz
multiqc path/to/folder/*_fastqc.zip

An overview of general statistics of sequence reads

Sample	% Duplicate Reads	% GC	Length	% Failed in FastQC	Reads (millions)
S1_Control-1_1	68.9%	50%	100 bp	27%	37.3
S1_Control-1_2	69.8%	51%	100 bp	27%	37.3
S2_LTR8KO-1_1	67.8%	49%	100 bp	27%	37.3
S2_LTR8KO-1_2	67.9%	50%	100 bp	27%	37.3
S5_Control-3_1	72.4%	50%	100 bp	27%	36.1
S5_Control-3_2	72.1%	51%	100 bp	27%	36.1
S6_LTR8KO-3_1	69.6%	49%	100 bp	27%	37.3
S6_LTR8KO-3_2	69.5%	50%	100 bp	27%	37.3
S7_Control-4_1	68.6%	50%	100 bp	27%	37.3
S7_Control-4_2	68.4%	51%	100 bp	27%	37.3
S8_LTR8KO-4_1	67.8%	50%	100 bp	27%	37.3
S8_LTR8KO-4_2	67.4%	51%	100 bp	27%	37.3

1.2. Mean quality value across each base position in the read.

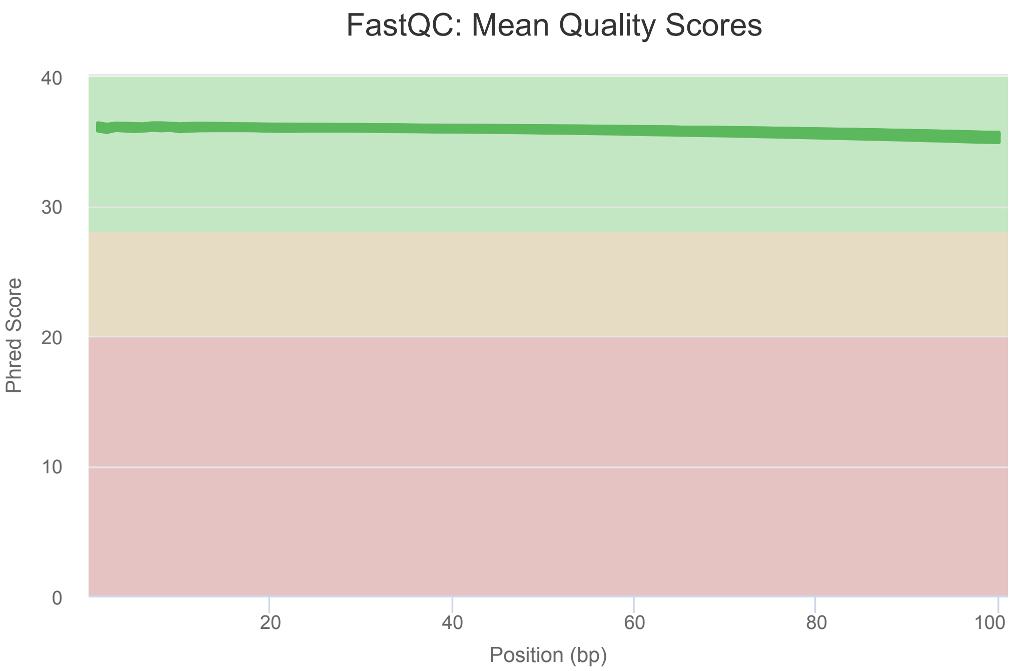

1.3. Reads alignment to reference human genome

Reads were aligned to the GRCh37/hg19 reference genome using the aligner 'Spliced Transcripts Alignment to a Reference (STAR)' {Dobin, 2013 #1297}. The alignment quality was further assessed by 'RSeQC' package {Wang, 2012 #1638}.

- Generating genome indexes files

```
--runThreadN 4 \
--runMode genomeGenerate \
--genomeDir /path/to/genomeDir \
--genomeFastaFiles /path/to/ GRCh37.p13.genome.fa \
--sjdbGTFfile /path/to/annotations.gff3 \
--sjdbGTFtagExonParentTranscript Parent \
--sjdbOverhang 99
```

- Mapping paired-end reads (e.g. S1_Control-1) to the hg19 reference genome

```
--runThreadN 4 \

--genomeDir /path/to/genomeDir \
--readFilesIn /path/to/S1_Control-1_1.fq.gz /path/to/S1_Control-1_2.fq.gz \
--readFilesCommand zcat \
--outSAMtype BAM SortedByCoordinate \
--outFileNamePrefix /path/to/output folder
```

1.4. Reads mapping summary

Sample	Input reads	Uniquely mapped reads (%)	Multiple mapping reads (%)	Unmapped reads (%)	Chimeric reads (%)
S1	37251546	93.85	5.75	3.39	0
S2	37295556	94.01	5.57	0.41	0
S5	36097204	93.46	6.18	0.37	0
S6	37267910	93.82	5.72	0.46	0
S7	37295246	93.68	5.88	0.44	0
S8	37253286	93.77	5.84	0.39	0

1.5. Quality control of alignment

The read-mapping quality was examined with the package 'RSeQC' following the instructions.

- saturation of sequencing depth

junction_saturation.py -i sample.bam -r hg19.refseq.bed12 -o output

- reads coverage over gene body

geneBody_coverage.py -r hg19.housekeeping.bed -i /path/to/the directory containing BAM files -o output

- inner distance (or insert size) between two paired RNA reads

inner_distance.py -i sample.bam -o output -r hg19.refseq.b

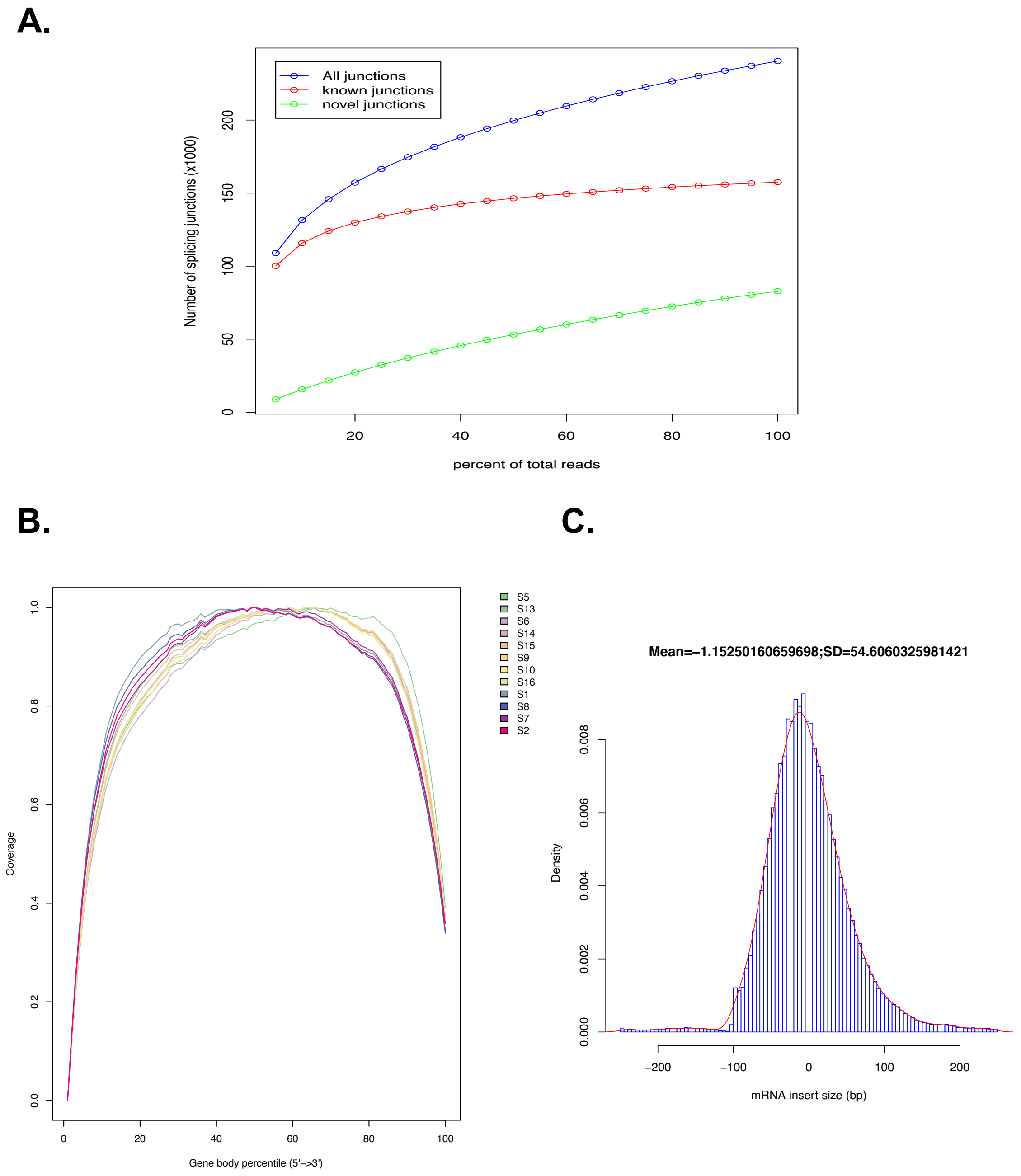

Figure. Quality control of reads mapping. (**A**) Saturation analysis of sequencing depth for splice junctions. One representative output shows current sequencing depth is saturated for ‘known junctions’ (red line) but not for ‘novel junctions’. (**B**) The RNA-seq reads coverage over gene body. (**C**) One representative output showing the inner distance (or insert size) between two paired RNA reads.

- Reads distribution over genome feature

read_distribution.py -i sample.bam -r hg19.refseq.bed12

1.6. One representative output showing RNA-seq reads distribution over genome feature.

Total Reads: 74157576

Total Tags 103633695

Total Assigned Tags 97730839

Group	Total_bases	Tag_count	Tags/Kb
CDS_Exons	35440972	73192762	2065.20
5'UTR_Exons	34573261	5607849	162.20
3'UTR_Exons	56295984	15264063	271.14
Introns	1444230997	3162355	2.19
TSS_up_1kb	30387574	59790	1.97
TSS_up_5kb	135838145	112870	0.83
TSS_up_10kb	243470833	149874	0.62
TES_down_1kb	32140566	146485	4.56
TES_down_5kb	139627575	287309	2.06
TES_down_10kb	246486380	353936	1.44

1.7. Counting reads

For reads quantitation, paired-end reads were managed with 'SAMtools' and counted using 'featureCounts' in the 'Rsubread' package {Liao, 2019 #513}.

- Re-sorting the BAM files generated by the 'STAR' aligner

samtools sort sample.bam -o sample.sorted.bam

- Indexing sorted BAM files

samtools index sample.sorted.bam

- Running 'featureCounts'

```
SAMPLE_DIR="path/to/the file folder containing all sample.sorted.bam"
TOOL_DIR="path/to/folder containing featureCount package"
for file in $(ls $SAMPLE_DIR/*.sorted.bam)
do
    sample='basename $file'
  $TOOL_DIR/featureCounts -p -T 4 -s 2 -a /path/to/gencode.v19.annotation.gtf -t exon -g
gene_id -o /path/to/output/$sample"_featureCounts.txt" $file
done
```

1.8. Differential expression analysis

The expression quantification was done by the package 'DEseq2' {Love, 2014 #1417} following the instructions. The p-values attained by the Wald test were corrected for multiple testing using the Benjamini and Hochberg method. The adjusted p-values (padj) were used to determine significant genes.

- Count matrix input

```
library("DESeq2")
dds <- DESeqDataSetFromMatrix(countData = cts,
                              colData = coldata,
                              design = ~ condition)
```

- Differential expression analysis

```
dds <- DESeq(dds)

res <- results(dds)
```

- Exporting results to CSV files

```
resOrdered <- res[order(res$pvalue),]

resSig <- subset(resOrdered, padj < 0.05)
write.csv(as.data.frame(resSig),
      file="resSig.csv")
```

- Results visualization

The differentially expressed genes were presented as volcano plots with the package 'EnhancedVolcano' following https://github.com/kevinblighe/EnhancedVolcano.

```
EnhancedVolcano(LTR8KO_resLFC,
          lab = rownames(LTR8KO_resLFC),
          x = 'log2FoldChange',
          y = 'padj',
          title = 'LTR8 KO versus Wild type',
          selectLab = c('CGA','CSH2',
'CSHL1','CYP11A1','CYP19A1','DEPDC1B','ERVW-
1','GDF15','GH2','HSD3B1','LGALS14','PSG9','PSG4','PSG3','PSG2','SDC1','ERVFRD-
1','CGB','LGALS16','LGALS3','NPNT','TEAD4','CNTNAP2','BIN1','CDX2','PODXL','HIF1A','
PLAC8'),
          xlab = bquote(~Log[2]~ 'fold change'),
          pCutoff = 10e-6,
          FCcutoff = 2,
          pointSize = 4.0,
          labSize = 6.0,
          labCol = 'black',
          boxedLabels = TRUE,
          colAlpha = 1,
          shape = c(1, 4, 23, 25),
          cutoffLineType = 'twodash',
          cutoffLineWidth = 1.0,
          drawConnectors = TRUE,
```

```
widthConnectors = 0.5,
colConnectors = 'black',
border = 'full',
borderWidth = 1.0,
borderColour = 'black')
```

1.9. Gene ontology analysis

For LTR8 KO and control samples, the top 1000 differently expressed genes were selected for gene ontology analysis with the online package 'Gorilla' {Mates, 2009 #1426}.

2. Human trophoblasts RNA sequencing

a) **Principal Component Analysis and Batch Effect Correction**

b) Gene expression data (TPM values) from 18 samples (8 control, 10 preeclampsia) were processed for principal component analysis (PCA). Low-expression genes were filtered using the following criteria: genes with maximum TPM < 0.1 across all samples or expressed in fewer than 2 samples were excluded. The filtered dataset was $\log_2$-transformed using $\log_2(\text{TPM} + 0.1)$ to handle zero values and reduce skewness.

c) **PCA Analysis:** The transformed expression matrix was standardized using z-score normalization, and PCA was performed using the scikit-learn library in Python. Principal components were calculated to capture the major sources of variation in the dataset.

d) **Batch Effect Correction:** To remove technical variation while preserving biological signal, batch correction was applied using [specify your actual method, e.g., ComBat, limma removeBatchEffect, or similar]. The correction enhanced group separation between control and preeclampsia samples while maintaining biological variance.

e) **Statistical Validation:** Group separation quality was quantified using the ratio of between-group distance to within-group variance. Statistical significance of group separation was assessed using Welch's t-test on PC1 coordinates. Visualization included 95% confidence ellipses around each group.

f) **Software:** Analysis was performed using Python 3.x with pandas, numpy, scikit-learn, and matplotlib libraries. High-quality figures were generated at 300 DPI resolution in SVG format for publication.

Mean Variance

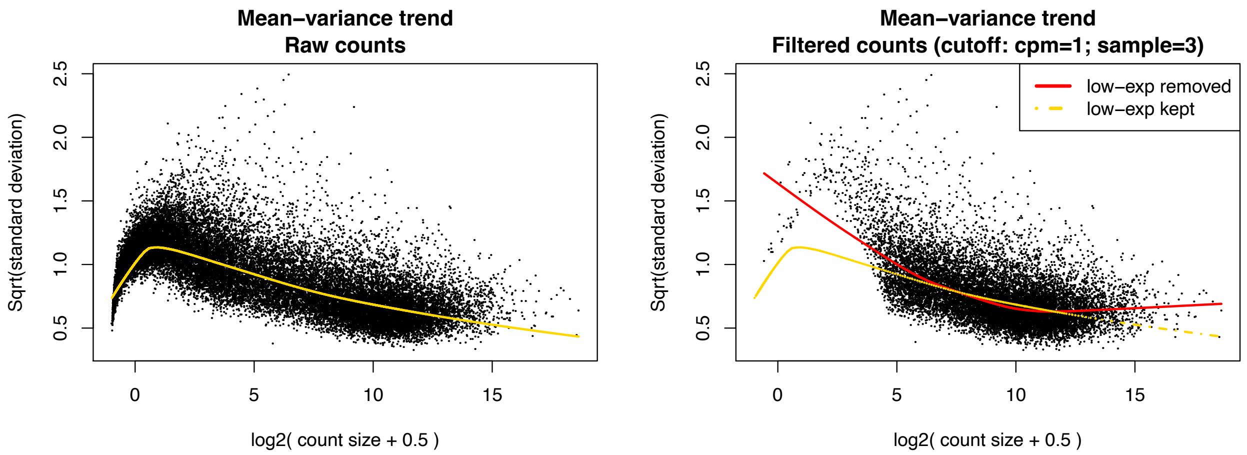

Transcript expression distribution

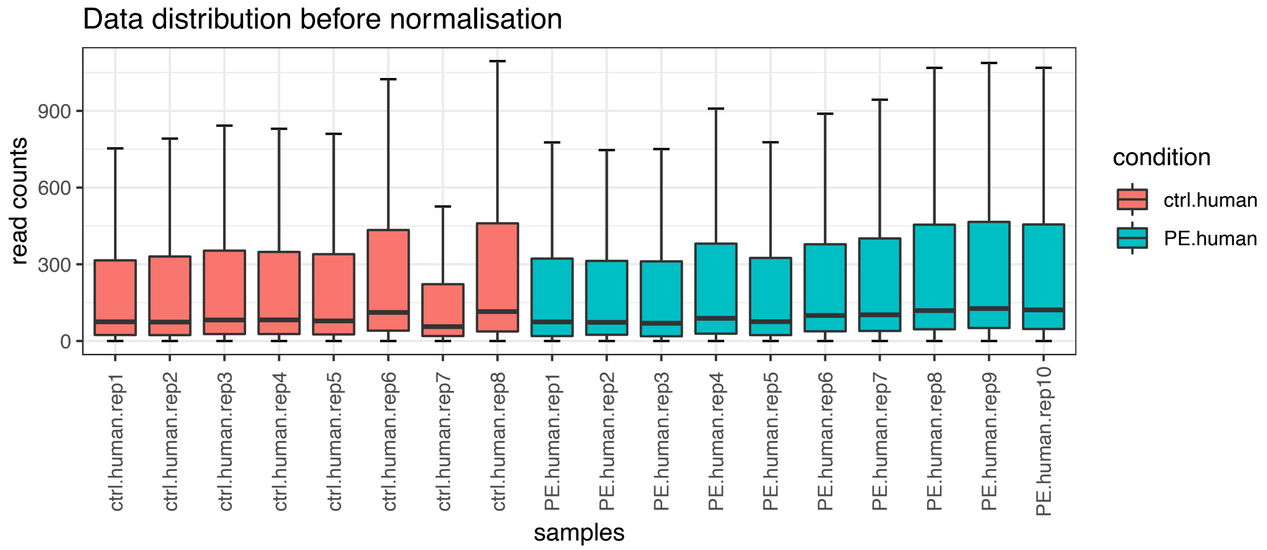

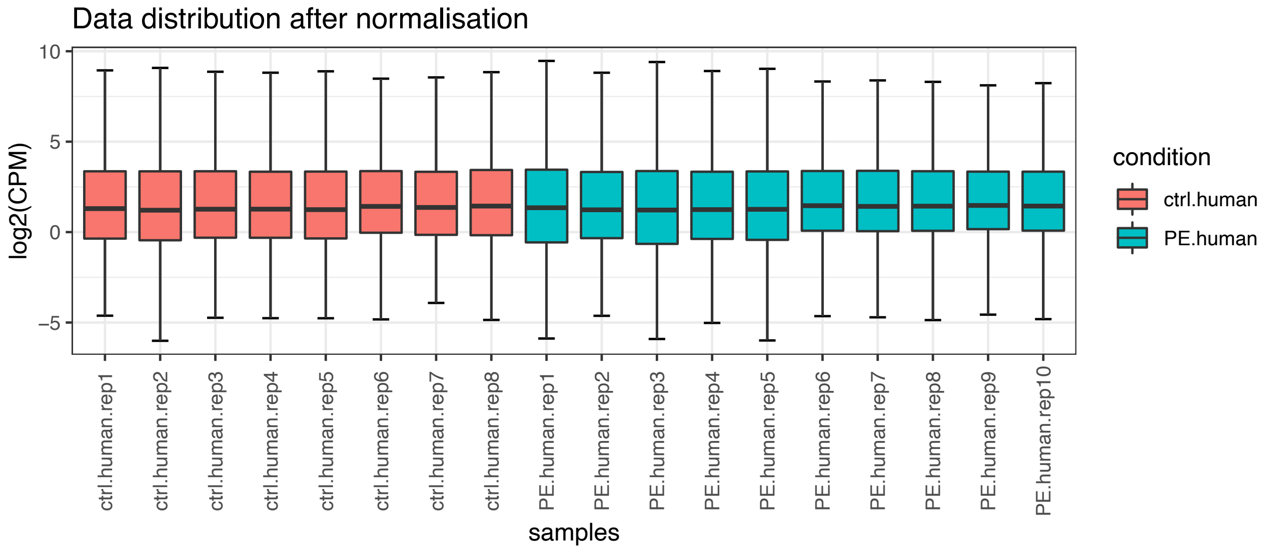

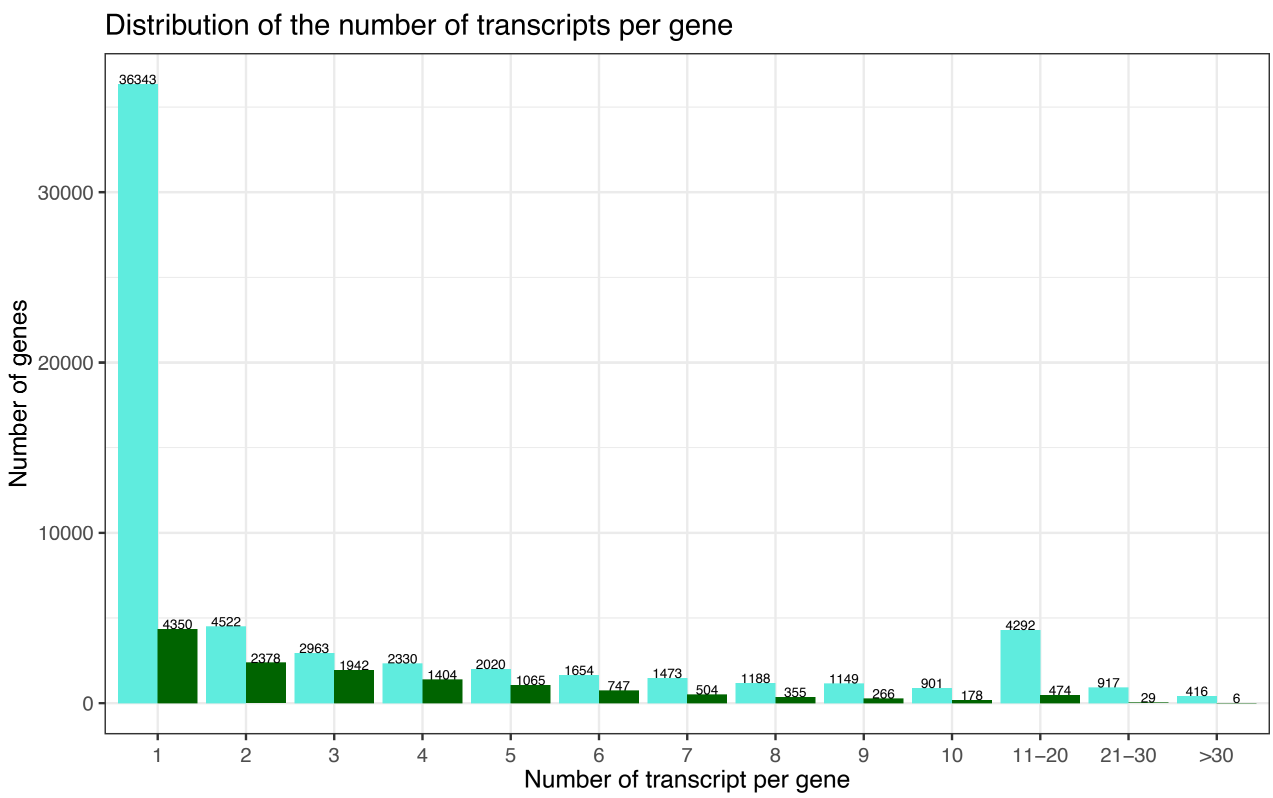

PCA_results

sample	group	color	short	PC1_original	PC2_original	PC1_corrected	PC2_corrected
**ctrl.human.rep1**	Control	#2E86AB	C.rep1	30.810996125787653	57.490542847450406	15.810996125787653	62.457684377562735
**ctrl.human.rep2**	Control	#2E86AB	C.rep2	21.269707123348926	25.224374031210296	6.269707123348926	23.84173101949845
**ctrl.human.rep3**	Control	#2E86AB	C.rep3	39.69536554220521	26.264999859993758	24.69536554220521	32.74188524100068
**ctrl.human.rep4**	Control	#2E86AB	C.rep4	26.665033477721252	31.983689870811656	11.665033477721252	47.21398843489191
**ctrl.human.rep5**	Control	#2E86AB	C.rep5	32.97105689410222	34.66674597498855	17.97105689410222	32.32521222775519
**ctrl.human.rep6**	Control	#2E86AB	C.rep6	-46.570017962055005	-11.792871949188928	-61.570017962055005	-14.134241518680733
**ctrl.human.rep7**	Control	#2E86AB	C.rep7	-38.10047212942296	-18.74181170498271	-53.10047212942296	-2.9496835499087943
**ctrl.human.rep8**	Control	#2E86AB	C.rep8	-61.13165383187499	-1.2926984009074949	-76.13165383187498	6.381648890621593
**PE.human.rep1**	PE	#A23B72	P.rep1	140.27309242602308	-124.41992587264245	155.27309242602308	-129.11466973199197
**PE.human.rep2**	PE	#A23B72	P.rep2	6.211843481633231	28.637793126397604	21.21184348163323	34.06339356225725
**PE.human.rep3**	PE	#A23B72	P.rep3	-2.8885372912069904	25.489604813810196	12.11146270879301	20.85542788568557
**PE.human.rep4**	PE	#A23B72	P.rep4	49.58109272305223	23.345915306659474	64.58109272305222	18.688617770956906
**PE.human.rep5**	PE	#A23B72	P.rep5	36.50486700598119	62.20330711353645	51.50486700598119	64.62292982919679
**PE.human.rep6**	PE	#A23B72	P.rep6	-37.911312765653996	-29.90431389160188	-22.911312765653996	-49.03711633817986
**PE.human.rep7**	PE	#A23B72	P.rep7	-50.189893212507066	-45.65711404701064	-35.189893212507066	-62.90629237214097
**PE.human.rep8**	PE	#A23B72	P.rep8	-47.814134049599225	-27.39720247214432	-32.814134049599225	-33.020077764554046
**PE.human.rep9**	PE	#A23B72	P.rep9	-30.494554158097912	-35.634066896932666	-15.494554158097912	-45.762378100276905
**PE.human.rep10**	PE	#A23B72	P.rep10	-68.8824793994369	-20.466967709447317	-53.8824793994369	-17.324494383494578

Raw read quality was checked with FastQC, removing reads with quality < Q30 and trimming 2 bases from each end. Each sample yielded at least 70 million high-quality reads. Reads were mapped to the human reference genome (hg19/GRCh37) and transcriptome (RefSeq GTF) using TopHat v2.0.8, Bowtie v2.0.5, and SAMtools 0.1.17, with parameters: -p 8 –r 150 --mate-std-dev 140 -library-type fr-firststrand. On average, 75% of reads mapped uniquely to annotated

genes, and ~10% to repetitive regions. Transcript quantification was performed using Cufflinks v2.0.8 (FPKM), and read counts generated with featureCounts (Subread). Differentially expressed genes (DEGs) were identified with DESeq2, using CPM normalization, negative binomial GLMs, and dispersion/fold change estimates. The number of total reads in each sample and number of uniquely mapped.
