# Supplementary material for: Endogenous retroviral elements LTR8B and MER65 rewire PSG9 regulation to control trophoblast syncytialization and pre-eclampsia risk
Source: Genome Biol. 2026 Mar 9;27:73. doi: 10.1186/s13059-026-03944-z (PMC12969887; doi:10.1186/s13059-026-03944-z)
Supplement: Supplementary file 5 — Additional file 5. Information on the PSG9 isoforms. [file 13059_2026_3944_MOESM5_ESM.pdf]

Name	Transcript ID	bp	Protein	Biotype	CCDS	UniProt	RefSeq Match	Flags
**PSG9-202**	ENST00000270077.8	1707	426aa	Protein coding	CCDS12618	Q00887-1	NM_002784.5	TSL:1GENCODE basicAPPRIS P2MANE Select v0.5
PSG9-206	ENST00000593948.5	1429	333aa	Protein coding	CCDS77314	M0R0U8	-	TSL:1GENCODE basic
PSG9-205	ENST00000443718.7	1421	333aa	Protein coding	CCDS77312	E7EW65	-	TSL:2GENCODE basic
PSG9-203	ENST00000291752.9	1055	240aa	Protein coding	CCDS77311	G3XAA7	-	TSL:1GENCODE basic
**PSG9-201**	ENST00000244293.11	2073	402aa	Protein coding	-	Q00887-2	-	TSL:2GENCODE basic
**PSG9-209**	ENST00000621109.4	2019	419aa	Protein coding	-	A0A087WYK1	-	TSL:5GENCODE basicAPPRIS ALT2
PSG9-208	ENST00000596730.1	1812	309aa	Protein coding	-	M0R0E4	-	TSL:3GENCODE basic
PSG9-204	ENST00000418820.6	1748	326aa	Protein coding	-	H7C1I4	-	TSL:5GENCODE basic
PSG9-207	ENST00000595404.1	2830	No protein	Retained intron	-	-	-	TSL:5
