# Supplementary material for: Endogenous retroviral elements LTR8B and MER65 rewire PSG9 regulation to control trophoblast syncytialization and pre-eclampsia risk
Source: Genome Biol. 2026 Mar 9;27:73. doi: 10.1186/s13059-026-03944-z (PMC12969887; doi:10.1186/s13059-026-03944-z)
Supplement: Supplementary file 6 — Additional file 6. Transmembrane prediction analysis of PSG family members. [file 13059_2026_3944_MOESM6_ESM.pdf]

TMHMM result

```
# ENST00000306322|ENST00000306322.1||PSG11 Length: 213
# ENST00000306322|ENST00000306322.1||PSG11 Number of predicted TMHs:  0
# ENST00000306322|ENST00000306322.1||PSG11 Exp number of AAs in TMHs: 0.00406
# ENST00000306322|ENST00000306322.1||PSG11 Exp number, first 60 AAs:  0.00024
# ENST00000306322|ENST00000306322.1||PSG11 Total prob of N-in:        0.01453
ENST00000306322|ENST00000306322.1||PSG11	TMHMM1.0	outside	     1   213
```

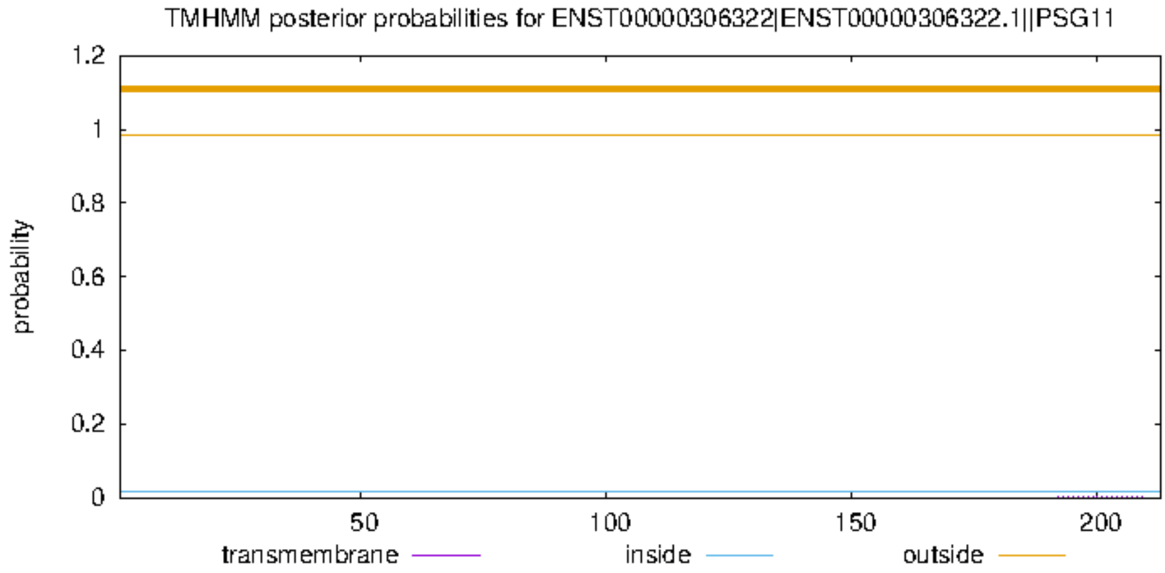

plot in postscript, script for making the plot in gnuplot, data for plot

```
# ENST00000443718|ENST00000443718.1|E7EW65|PSG9 Length: 333
# ENST00000443718|ENST00000443718.1|E7EW65|PSG9 Number of predicted TMHs:  0
# ENST00000443718|ENST00000443718.1|E7EW65|PSG9 Exp number of AAs in TMHs: 0.23632
# ENST00000443718|ENST00000443718.1|E7EW65|PSG9 Exp number, first 60 AAs:  0.03281
# ENST00000443718|ENST00000443718.1|E7EW65|PSG9 Total prob of N-in:        0.00938
ENST00000443718|ENST00000443718.1|E7EW65|PSG9	TMHMM1.0	outside	     1   333
```

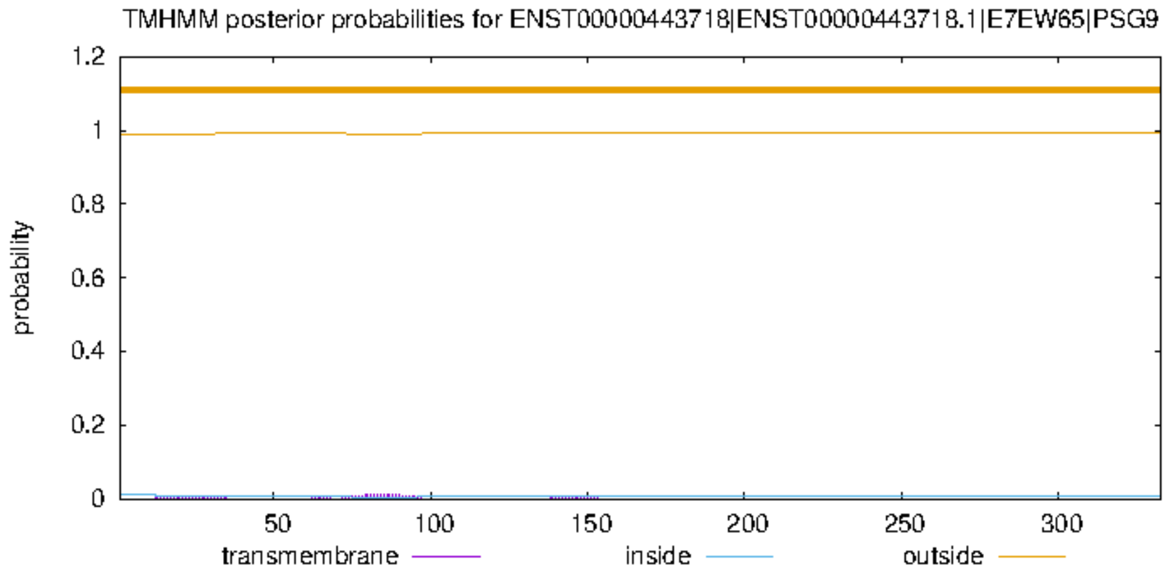

plot in postscript, script for making the plot in gnuplot, data for plot

```
# ENST00000404580|ENST00000404580.1|E7EQY3|PSG5 Length: 333
# ENST00000404580|ENST00000404580.1|E7EQY3|PSG5 Number of predicted TMHs:  0
# ENST00000404580|ENST00000404580.1|E7EQY3|PSG5 Exp number of AAs in TMHs: 0.47659
# ENST00000404580|ENST00000404580.1|E7EQY3|PSG5 Exp number, first 60 AAs:  0.44586
# ENST00000404580|ENST00000404580.1|E7EQY3|PSG5 Total prob of N-in:        0.02063
ENST00000404580|ENST00000404580.1|E7EQY3|PSG5	TMHMM1.0	outside	     1   333
```

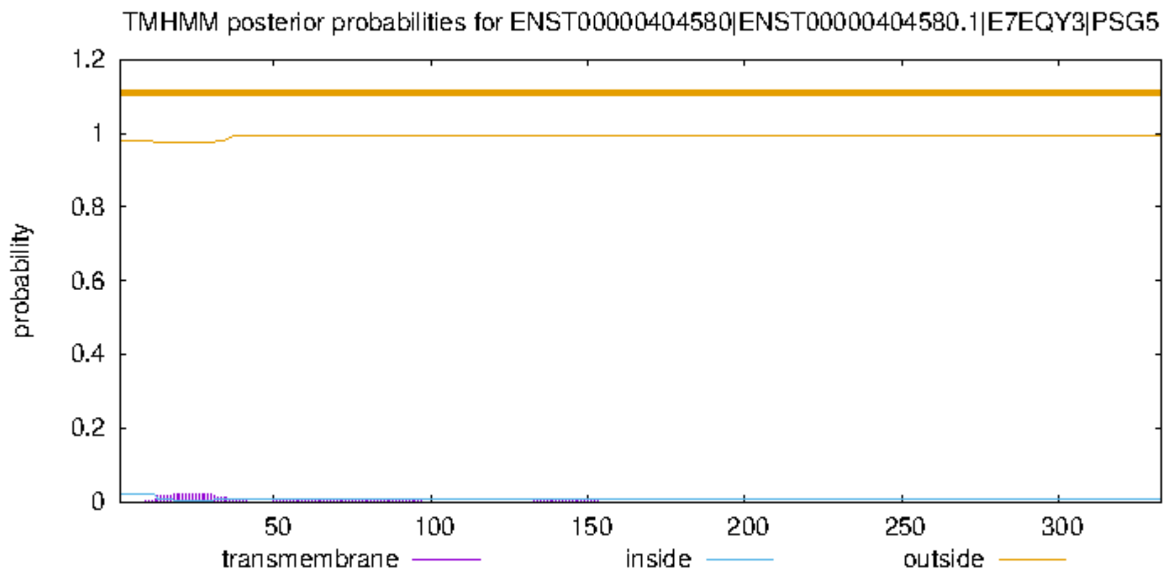

plot in postscript, script for making the plot in gnuplot, data for plot

---

```
# ENST00000594378|ENST00000594378.1|M0QXP2|PSG3 Length: 91
# ENST00000594378|ENST00000594378.1|M0QXP2|PSG3 Number of predicted TMHs:  0
# ENST00000594378|ENST00000594378.1|M0QXP2|PSG3 Exp number of AAs in TMHs: 1.02208
# ENST00000594378|ENST00000594378.1|M0QXP2|PSG3 Exp number, first 60 AAs:  1.02184
# ENST00000594378|ENST00000594378.1|M0QXP2|PSG3 Total prob of N-in:        0.39743
ENST00000594378|ENST00000594378.1|M0QXP2|PSG3   TMHMM1.0        outside      1    91
```

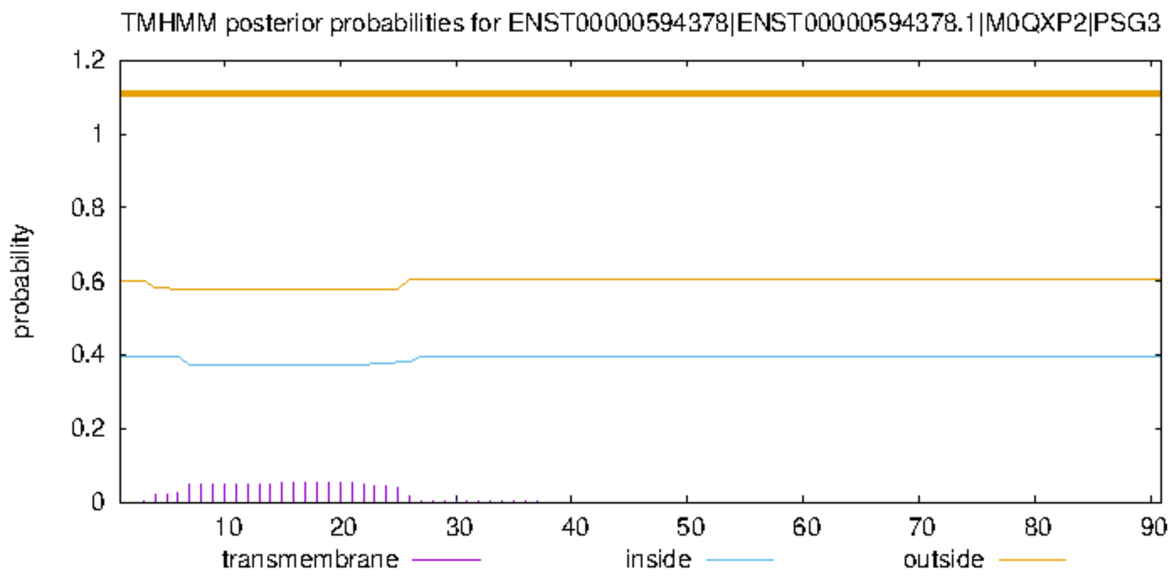

plot in postscript, script for making the plot in gnuplot, data for plot

---

```
# ENST00000270077|ENST00000270077.1||PSG9 Length: 426
# ENST00000270077|ENST00000270077.1||PSG9 Number of predicted TMHs:  0
# ENST00000270077|ENST00000270077.1||PSG9 Exp number of AAs in TMHs: 0.1688
# ENST00000270077|ENST00000270077.1||PSG9 Exp number, first 60 AAs:  0.02987
# ENST00000270077|ENST00000270077.1||PSG9 Total prob of N-in:        0.00729
ENST00000270077|ENST00000270077.1||PSG9 TMHMM1.0        outside      1   426
```

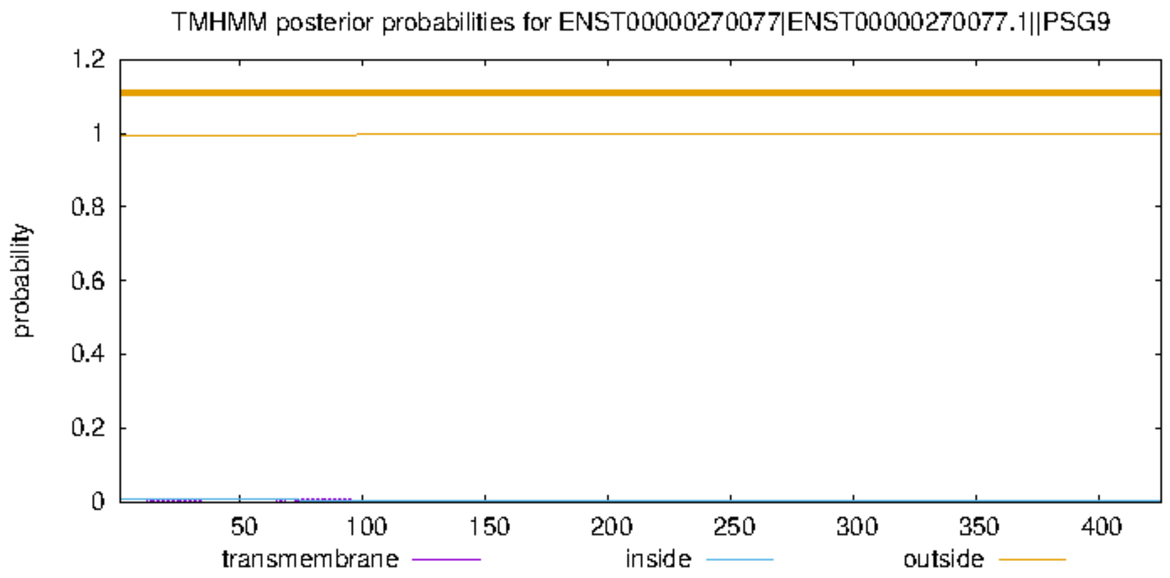

plot in postscript, script for making the plot in gnuplot, data for plot

---

```
# ENST00000244295|ENST00000244295.1||PSG4 Length: 326
# ENST00000244295|ENST00000244295.1||PSG4 Number of predicted TMHs:  0
# ENST00000244295|ENST00000244295.1||PSG4 Exp number of AAs in TMHs: 0.10412
# ENST00000244295|ENST00000244295.1||PSG4 Exp number, first 60 AAs:  0.04476
# ENST00000244295|ENST00000244295.1||PSG4 Total prob of N-in:        0.00571
ENST00000244295|ENST00000244295.1||PSG4 TMHMM1.0        outside      1   326
```

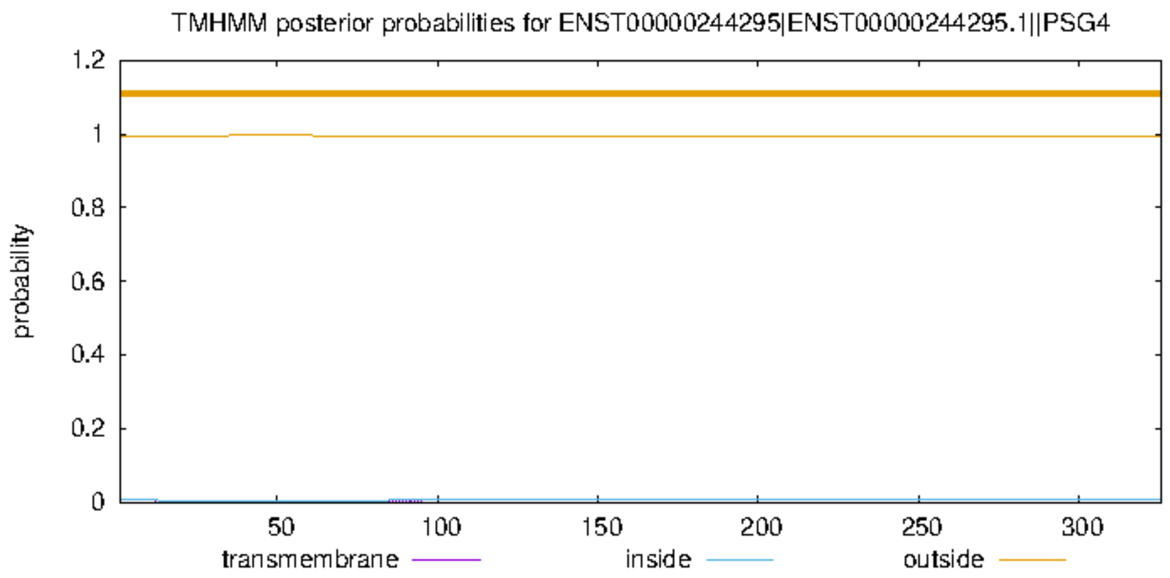

plot in postscript, script for making the plot in gnuplot, data for plot

---

```
# ENST00000407356|ENST00000407356.1||PSG5 Length: 335
# ENST00000407356|ENST00000407356.1||PSG5 Number of predicted TMHs:  0
# ENST00000407356|ENST00000407356.1||PSG5 Exp number of AAs in TMHs: 0.47535
# ENST00000407356|ENST00000407356.1||PSG5 Exp number, first 60 AAs:  0.44477
# ENST00000407356|ENST00000407356.1||PSG5 Total prob of N-in:        0.02058
ENST00000407356|ENST00000407356.1||PSG5 TMHMM1.0        outside      1   335
```

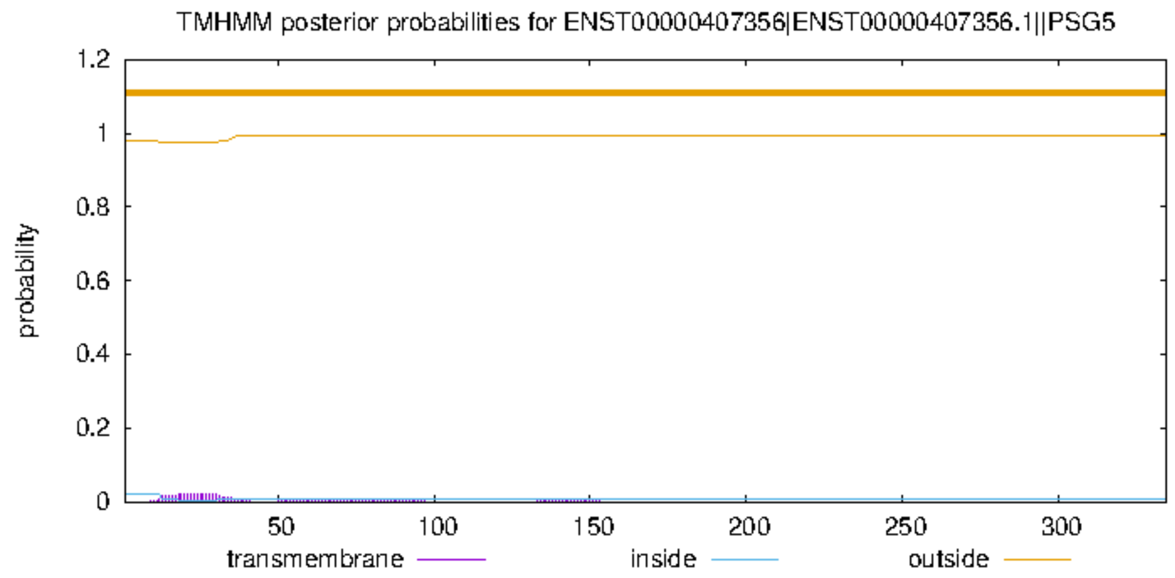

[plot](#) in postscript, [script](#) for making the plot in gnuplot, [data](#) for plot

---

```
# ENST00000244296|ENST00000244296.1||PSG1 Length: 426
# ENST00000244296|ENST00000244296.1||PSG1 Number of predicted TMHs:  0
# ENST00000244296|ENST00000244296.1||PSG1 Exp number of AAs in TMHs: 0.20757
# ENST00000244296|ENST00000244296.1||PSG1 Exp number, first 60 AAs:  0.12401
# ENST00000244296|ENST00000244296.1||PSG1 Total prob of N-in:        0.01098
ENST00000244296|ENST00000244296.1||PSG1 TMHMM1.0        outside      1   426
```

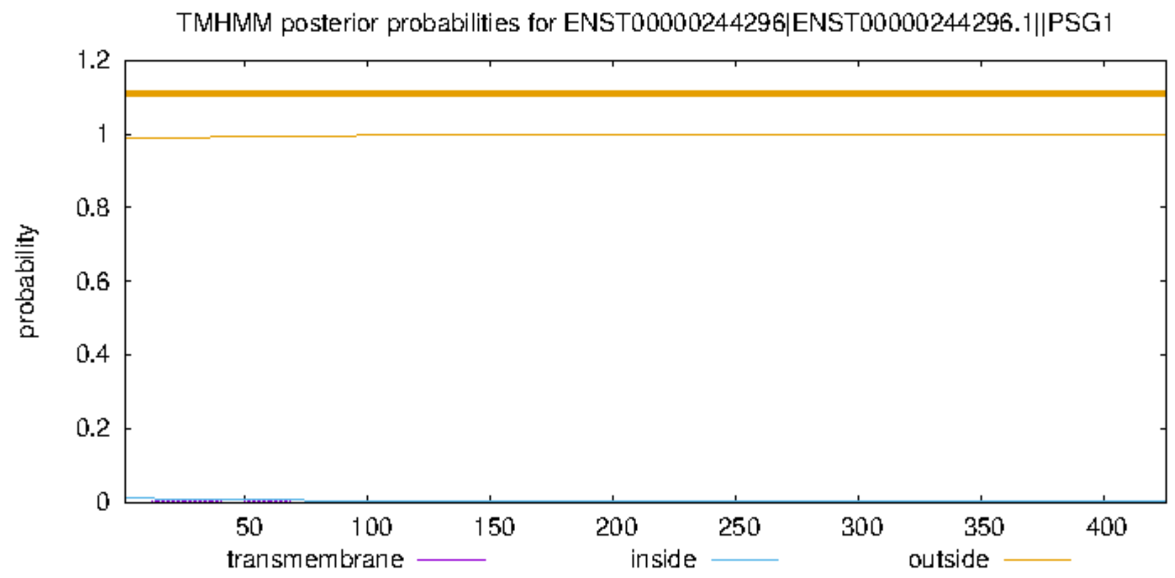

[plot](#) in postscript, [script](#) for making the plot in gnuplot, [data](#) for plot

---

```
# ENST00000366175|ENST00000366175.1||PSG5 Length: 335
# ENST00000366175|ENST00000366175.1||PSG5 Number of predicted TMHs:  0
# ENST00000366175|ENST00000366175.1||PSG5 Exp number of AAs in TMHs: 0.47535
# ENST00000366175|ENST00000366175.1||PSG5 Exp number, first 60 AAs:  0.44477
# ENST00000366175|ENST00000366175.1||PSG5 Total prob of N-in:        0.02058
ENST00000366175|ENST00000366175.1||PSG5 TMHMM1.0        outside      1   335
```

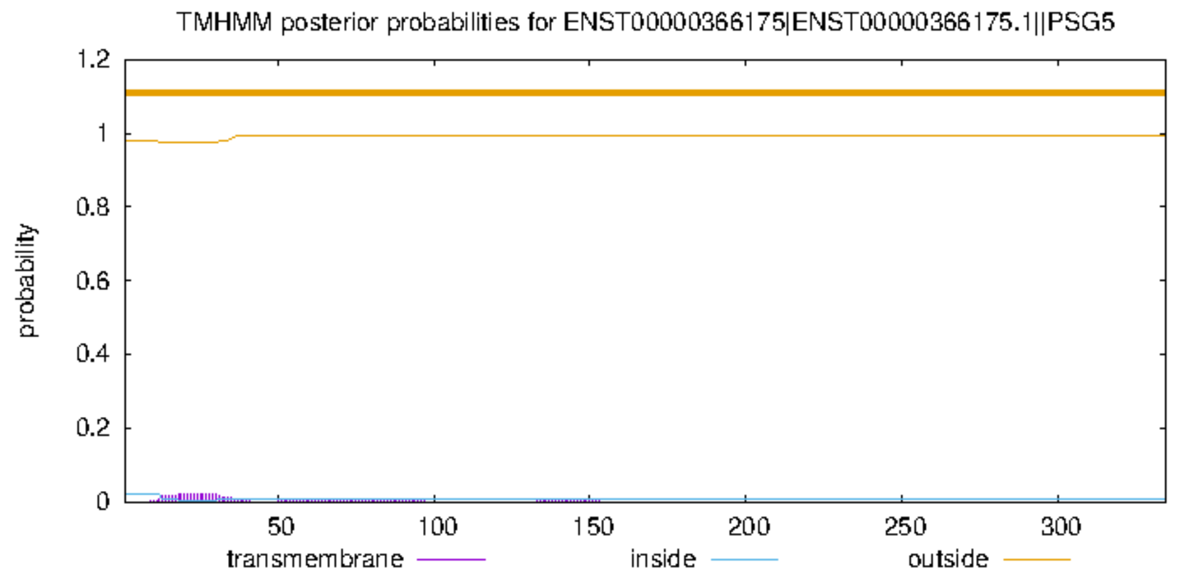

[plot](#) in postscript, [script](#) for making the plot in gnuplot, [data](#) for plot

---

```
# ENST00000446844|ENST00000446844.1|A0A087WT09|PSG7 Length: 426
# ENST00000446844|ENST00000446844.1|A0A087WT09|PSG7 Number of predicted TMHs:  0
# ENST00000446844|ENST00000446844.1|A0A087WT09|PSG7 Exp number of AAs in TMHs: 0.02238
# ENST00000446844|ENST00000446844.1|A0A087WT09|PSG7 Exp number, first 60 AAs:  0.0204
# ENST00000446844|ENST00000446844.1|A0A087WT09|PSG7 Total prob of N-in:        0.00175
ENST00000446844|ENST00000446844.1|A0A087WT09|PSG7	TMHMM1.0	outside	     1   426
```

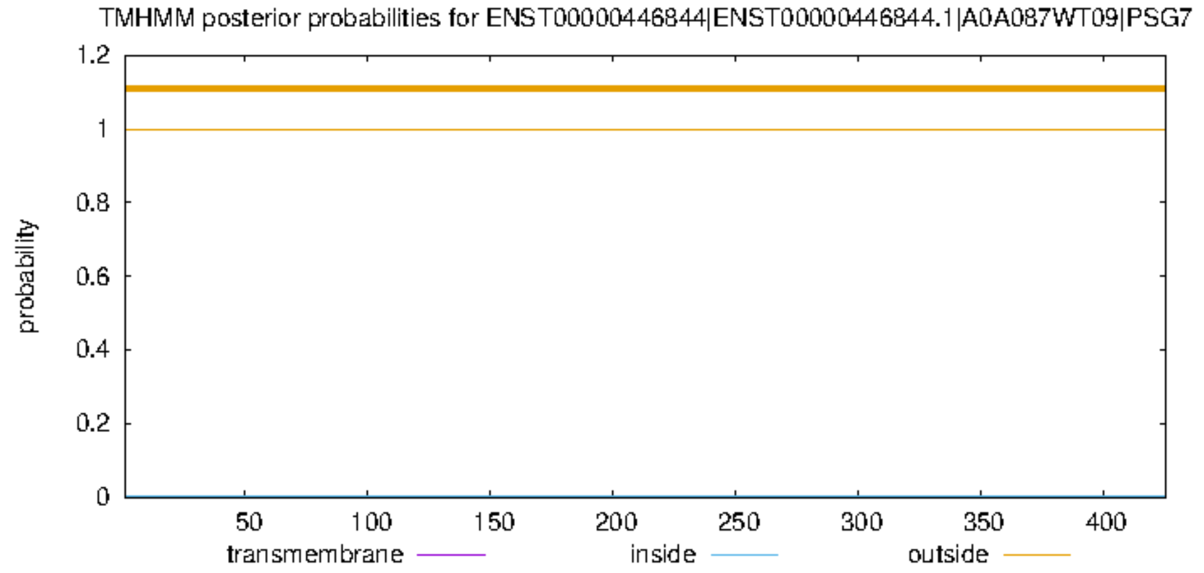

[plot](#) in postscript, [script](#) for making the plot in gnuplot, [data](#) for plot

---

```
# ENST00000433626|ENST00000433626.1||PSG4 Length: 326
# ENST00000433626|ENST00000433626.1||PSG4 Number of predicted TMHs:  0
# ENST00000433626|ENST00000433626.1||PSG4 Exp number of AAs in TMHs: 0.10321
# ENST00000433626|ENST00000433626.1||PSG4 Exp number, first 60 AAs:  0.04474
# ENST00000433626|ENST00000433626.1||PSG4 Total prob of N-in:        0.00570
ENST00000433626|ENST00000433626.1||PSG4 TMHMM1.0	outside	     1   326
```

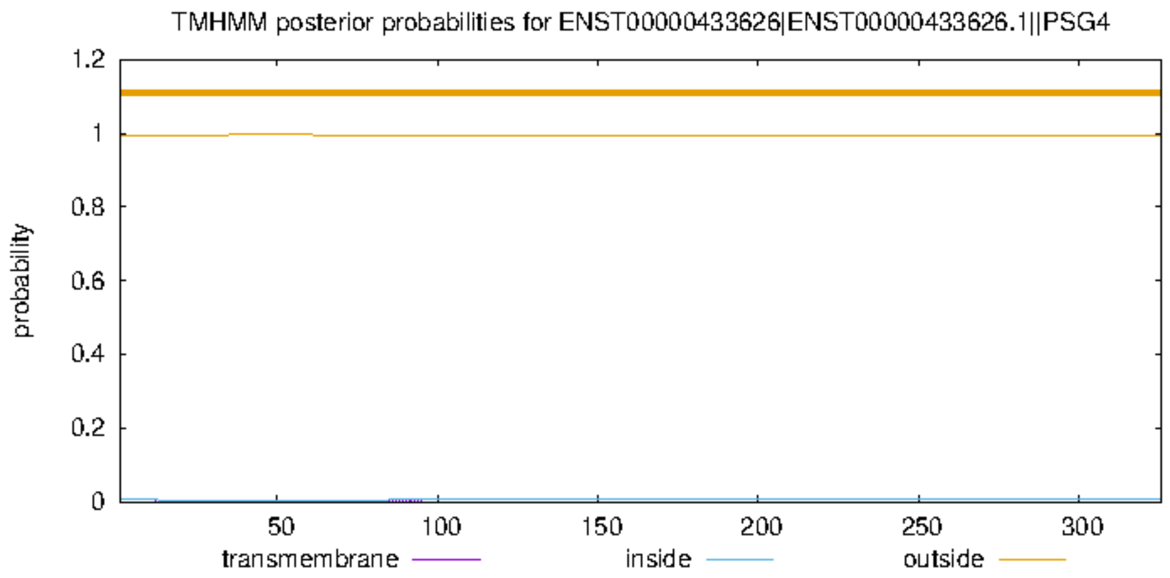

[plot](#) in postscript, [script](#) for making the plot in gnuplot, [data](#) for plot

---

```
# ENST00000306511|ENST00000306511.1||PSG8 Length: 426
# ENST00000306511|ENST00000306511.1||PSG8 Number of predicted TMHs:  0
# ENST00000306511|ENST00000306511.1||PSG8 Exp number of AAs in TMHs: 0.0699699999999999
# ENST00000306511|ENST00000306511.1||PSG8 Exp number, first 60 AAs:  0.02917
# ENST00000306511|ENST00000306511.1||PSG8 Total prob of N-in:        0.00364
ENST00000306511|ENST00000306511.1||PSG8 TMHMM1.0        outside      1   426
```

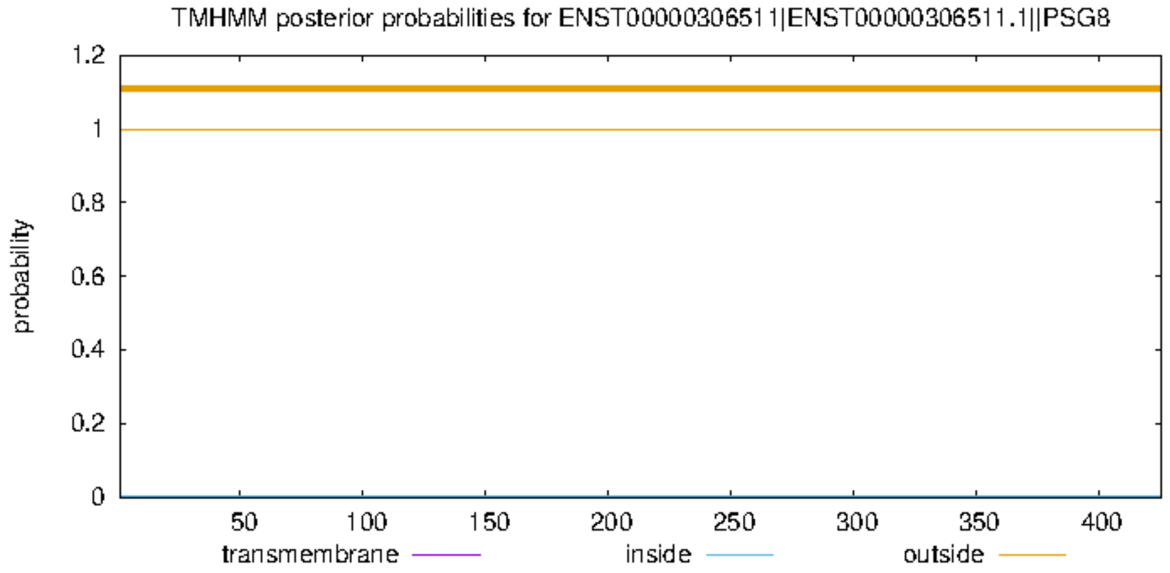

[plot](#) in postscript, [script](#) for making the plot in gnuplot, [data](#) for plot

---

```
# ENST00000478387|ENST00000478387.1|M0QYP3|PSG8 Length: 40
# ENST00000478387|ENST00000478387.1|M0QYP3|PSG8 Number of predicted TMHs:  0
# ENST00000478387|ENST00000478387.1|M0QYP3|PSG8 Exp number of AAs in TMHs: 0.02253
# ENST00000478387|ENST00000478387.1|M0QYP3|PSG8 Exp number, first 60 AAs:  0.02253
# ENST00000478387|ENST00000478387.1|M0QYP3|PSG8 Total prob of N-in:        0.08291
ENST00000478387|ENST00000478387.1|M0QYP3|PSG8   TMHMM1.0        outside      1    40
```

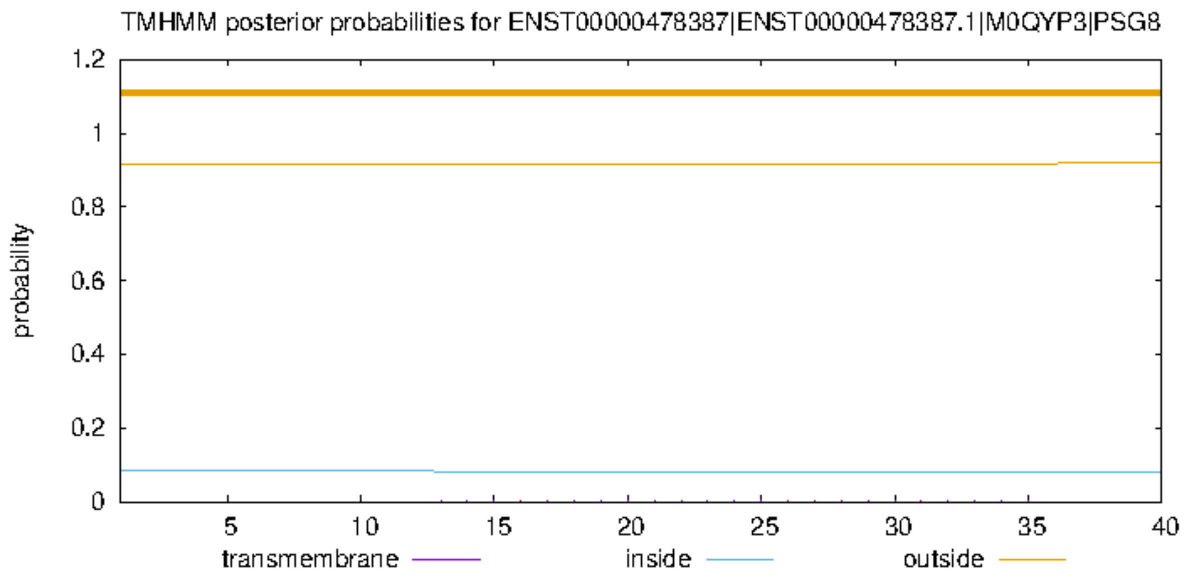

plot in postscript, script for making the plot in gnuplot, data for plot

---

```
# ENST00000292125|ENST00000292125.1||PSG6 Length: 435
# ENST00000292125|ENST00000292125.1||PSG6 Number of predicted TMHs:  0
# ENST00000292125|ENST00000292125.1||PSG6 Exp number of AAs in TMHs: 0.39474
# ENST00000292125|ENST00000292125.1||PSG6 Exp number, first 60 AAs:  0.25916
# ENST00000292125|ENST00000292125.1||PSG6 Total prob of N-in:        0.01219
ENST00000292125|ENST00000292125.1||PSG6 TMHMM1.0        outside      1   435
```

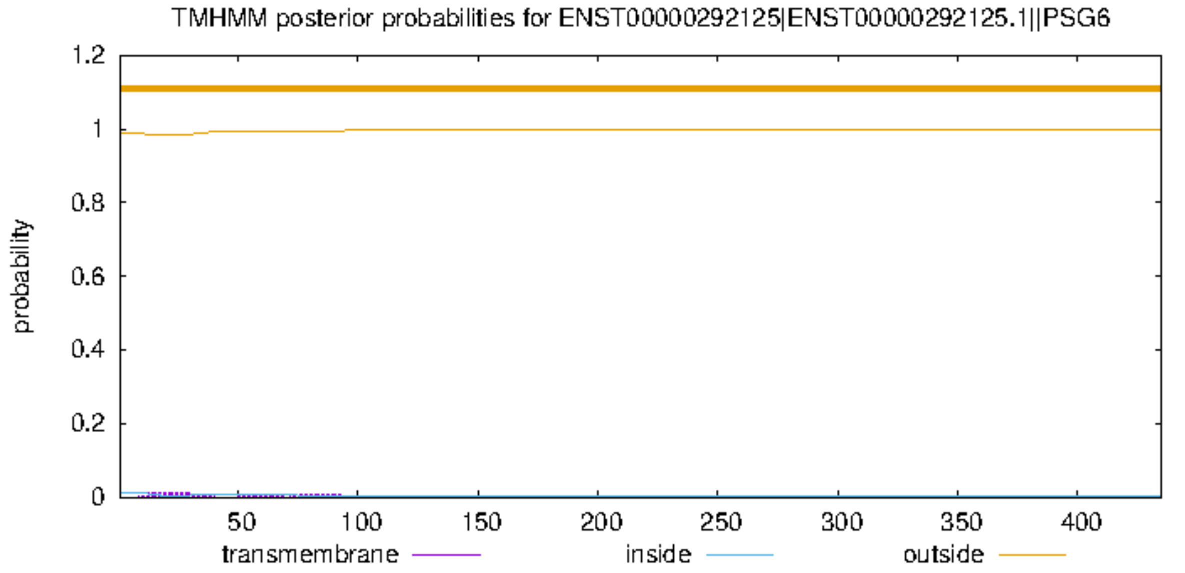

plot in postscript, script for making the plot in gnuplot, data for plot

---

```
# ENST00000436291|ENST00000436291.1||PSG1 Length: 419
# ENST00000436291|ENST00000436291.1||PSG1 Number of predicted TMHs:  0
# ENST00000436291|ENST00000436291.1||PSG1 Exp number of AAs in TMHs: 0.20808
# ENST00000436291|ENST00000436291.1||PSG1 Exp number, first 60 AAs:  0.12421
# ENST00000436291|ENST00000436291.1||PSG1 Total prob of N-in:        0.01113
ENST00000436291|ENST00000436291.1||PSG1 TMHMM1.0        outside      1   419
```

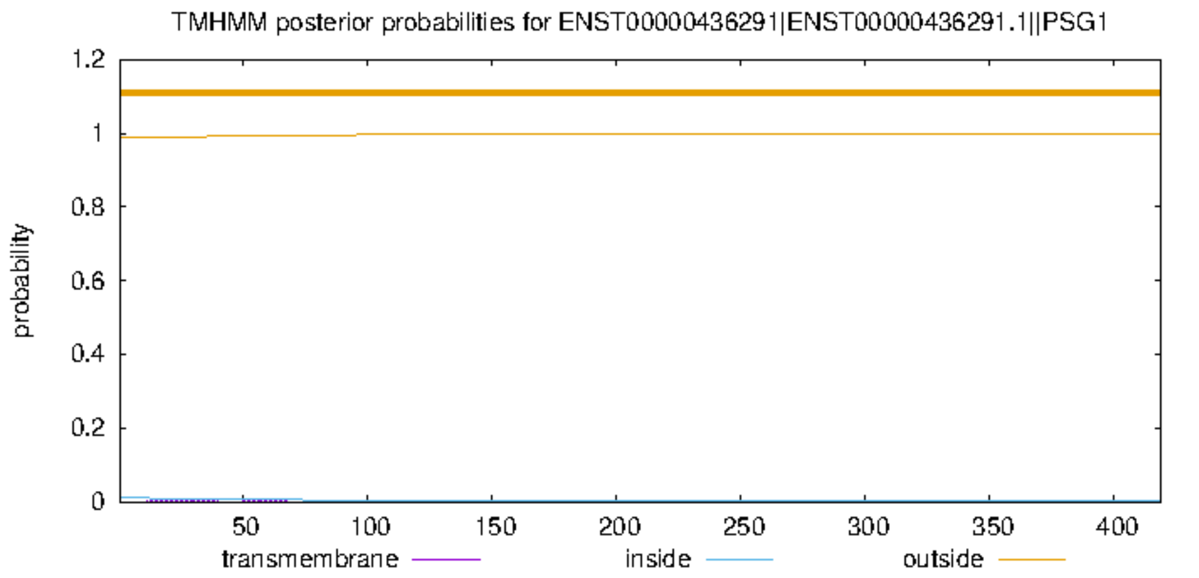

plot in postscript, script for making the plot in gnuplot, data for plot

---

```
# ENST00000329509|ENST00000329509.1|A0A0A0MR77|PSG2 Length: 117
# ENST00000329509|ENST00000329509.1|A0A0A0MR77|PSG2 Number of predicted TMHs:  0
# ENST00000329509|ENST00000329509.1|A0A0A0MR77|PSG2 Exp number of AAs in TMHs: 0.00364
# ENST00000329509|ENST00000329509.1|A0A0A0MR77|PSG2 Exp number, first 60 AAs:  0.00191
# ENST00000329509|ENST00000329509.1|A0A0A0MR77|PSG2 Total prob of N-in:        0.36562
ENST00000329509|ENST00000329509.1|A0A0A0MR77|PSG2       TMHMM1.0        outside      1   117
```

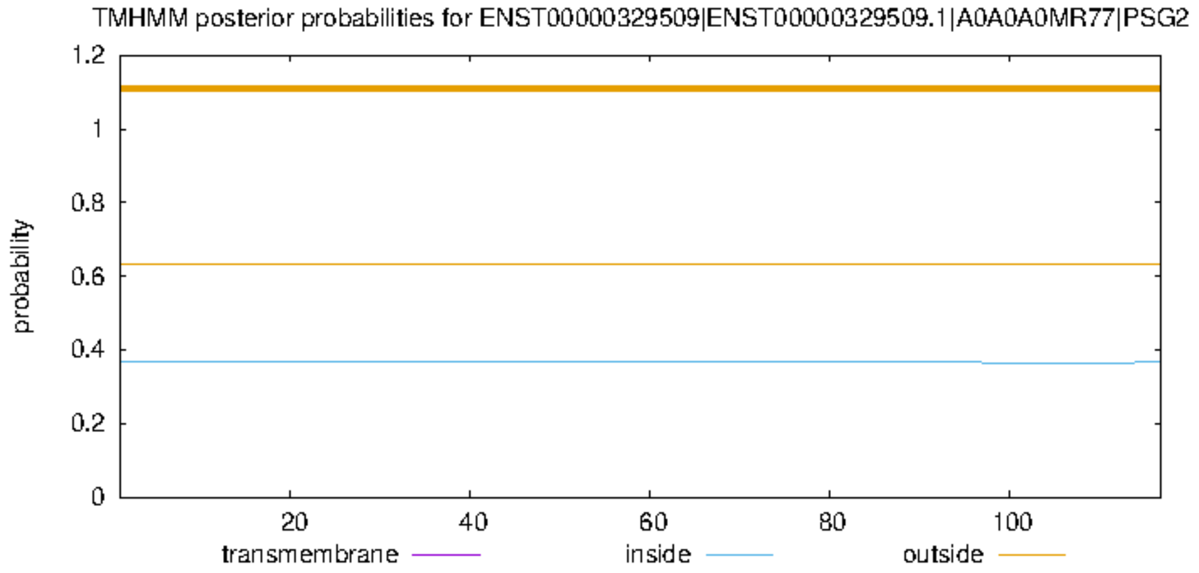

plot in postscript, script for making the plot in gnuplot, data for plot

---

```
# ENST00000291752|ENST00000291752.1|G3XAA7|PSG9 Length: 240
# ENST00000291752|ENST00000291752.1|G3XAA7|PSG9 Number of predicted TMHs:  0
# ENST00000291752|ENST00000291752.1|G3XAA7|PSG9 Exp number of AAs in TMHs: 0.44496
# ENST00000291752|ENST00000291752.1|G3XAA7|PSG9 Exp number, first 60 AAs:  0.04266
# ENST00000291752|ENST00000291752.1|G3XAA7|PSG9 Total prob of N-in:        0.01628
ENST00000291752|ENST00000291752.1|G3XAA7|PSG9   TMHMM1.0        outside      1   240
```

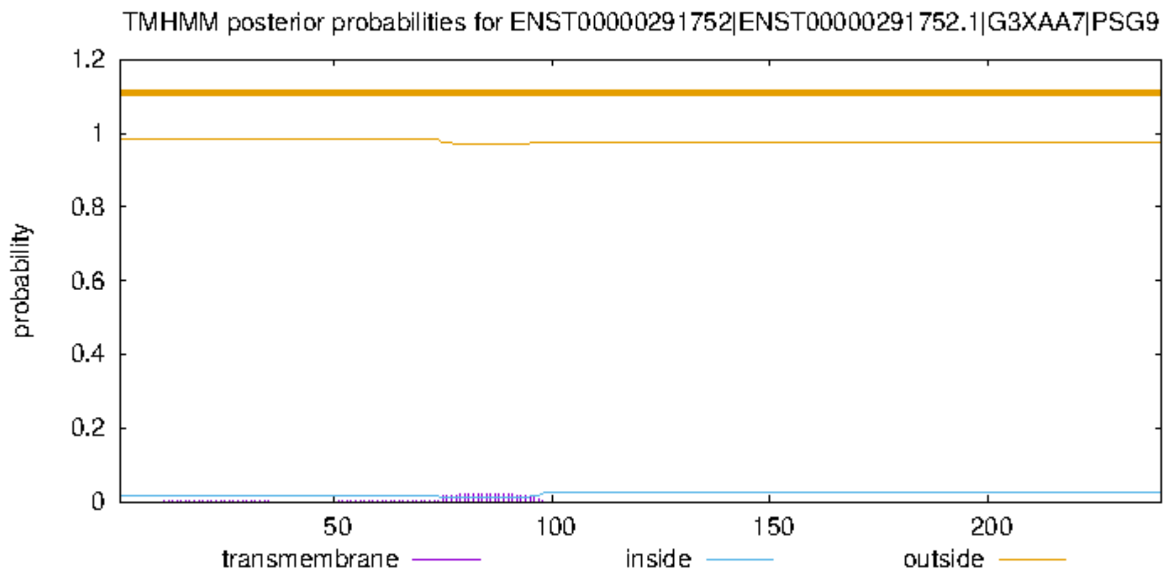

[plot](#) in postscript, [script](#) for making the plot in gnuplot, [data](#) for plot

---

```
# ENST00000593983|ENST00000593983.1|M0R2P9|PSG11 Length: 126
# ENST00000593983|ENST00000593983.1|M0R2P9|PSG11 Number of predicted TMHs:  0
# ENST00000593983|ENST00000593983.1|M0R2P9|PSG11 Exp number of AAs in TMHs: 0.674379999999999
# ENST00000593983|ENST00000593983.1|M0R2P9|PSG11 Exp number, first 60 AAs:  0.65312
# ENST00000593983|ENST00000593983.1|M0R2P9|PSG11 Total prob of N-in:        0.17752
ENST00000593983|ENST00000593983.1|M0R2P9|PSG11  TMHMM1.0        outside      1   126
```

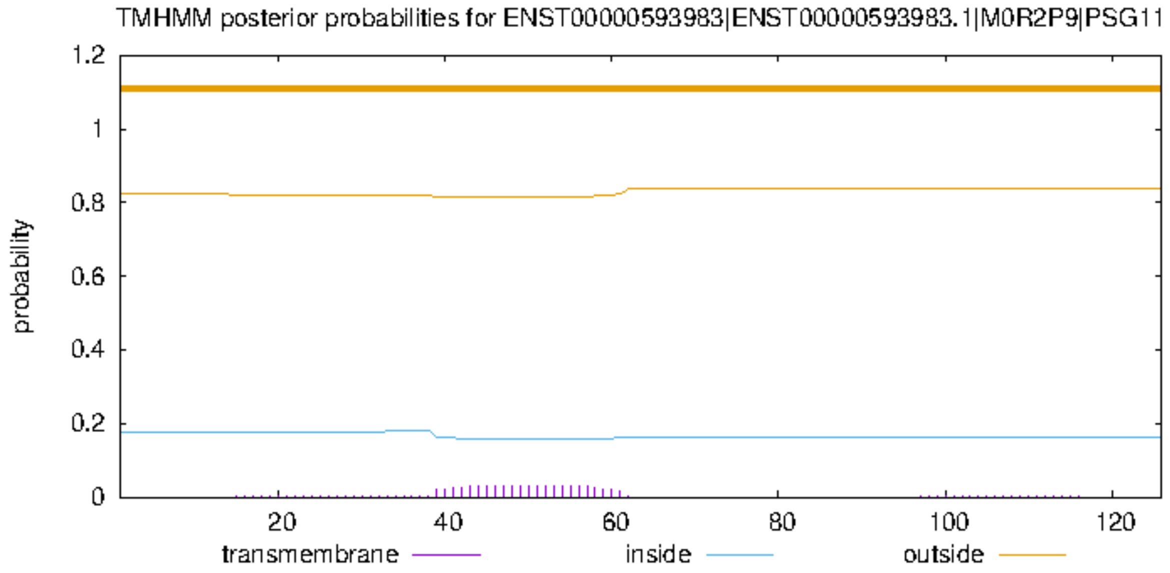

[plot](#) in postscript, [script](#) for making the plot in gnuplot, [data](#) for plot

---

```
# ENST00000403486|ENST00000403486.1||PSG11 Length: 213
# ENST00000403486|ENST00000403486.1||PSG11 Number of predicted TMHs:  0
# ENST00000403486|ENST00000403486.1||PSG11 Exp number of AAs in TMHs: 0.00406
# ENST00000403486|ENST00000403486.1||PSG11 Exp number, first 60 AAs:  0.00024
# ENST00000403486|ENST00000403486.1||PSG11 Total prob of N-in:        0.01453
ENST00000403486|ENST00000403486.1||PSG11        TMHMM1.0        outside      1   213
```

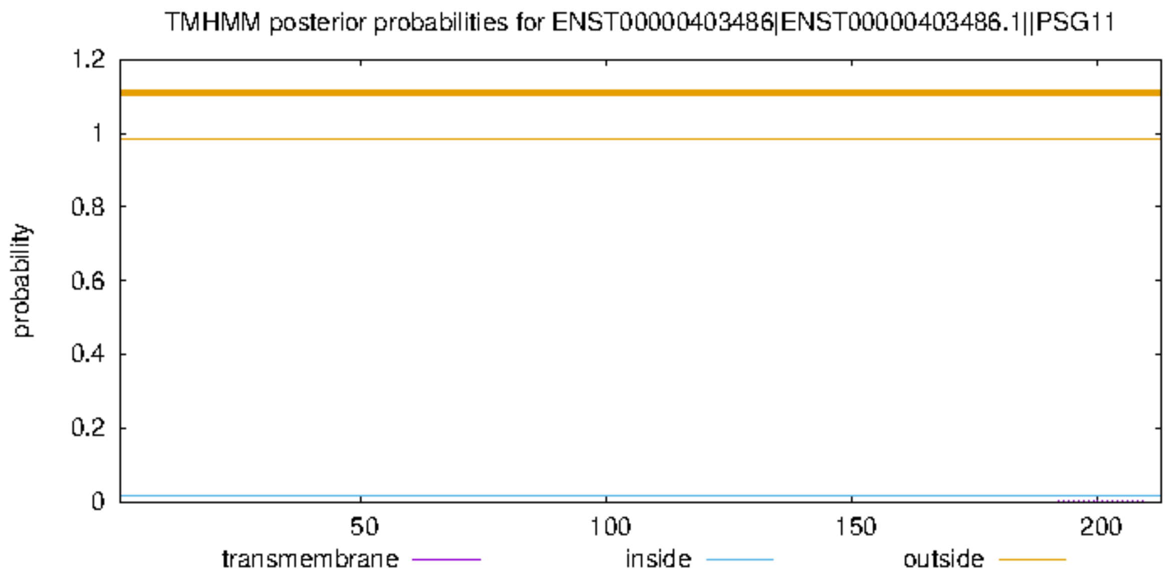

[plot](#) in postscript, [script](#) for making the plot in gnuplot, [data](#) for plot

---

```
# ENST00000342951|ENST00000342951.1||PSG5 Length: 335
# ENST00000342951|ENST00000342951.1||PSG5 Number of predicted TMHs:  0
# ENST00000342951|ENST00000342951.1||PSG5 Exp number of AAs in TMHs: 0.47535
# ENST00000342951|ENST00000342951.1||PSG5 Exp number, first 60 AAs:  0.44477
# ENST00000342951|ENST00000342951.1||PSG5 Total prob of N-in:        0.02058
ENST00000342951|ENST00000342951.1||PSG5 TMHMM1.0        outside      1   335
```

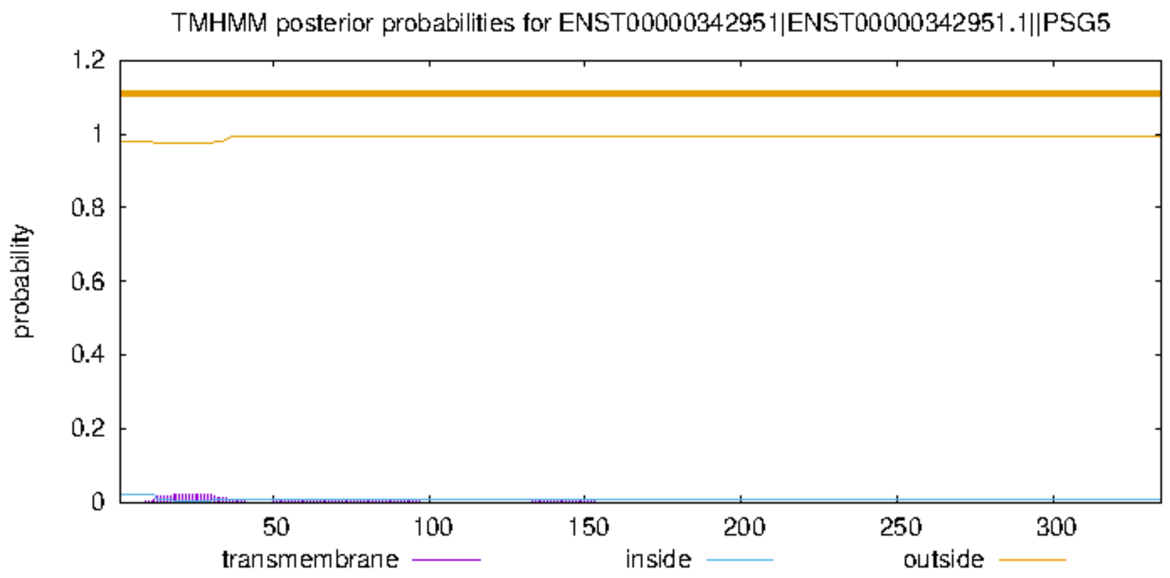

[plot](#) in postscript, [script](#) for making the plot in gnuplot, [data](#) for plot

---

```
# ENST00000312439|ENST00000312439.1||PSG1 Length: 428
# ENST00000312439|ENST00000312439.1||PSG1 Number of predicted TMHs:  0
# ENST00000312439|ENST00000312439.1||PSG1 Exp number of AAs in TMHs: 0.2088
# ENST00000312439|ENST00000312439.1||PSG1 Exp number, first 60 AAs:  0.12459
# ENST00000312439|ENST00000312439.1||PSG1 Total prob of N-in:        0.01140
ENST00000312439|ENST00000312439.1||PSG1 TMHMM1.0        outside      1   428
```

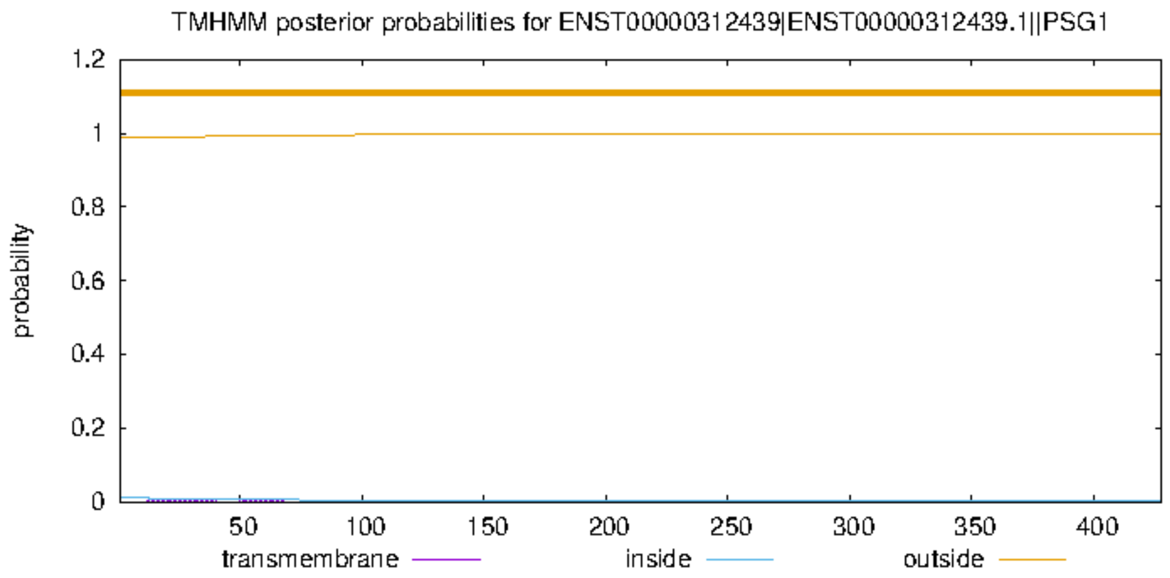

plot in postscript, script for making the plot in gnuplot, data for plot

---

```
# ENST00000451895|ENST00000451895.1|C9JWP2|PSG4 Length: 252
# ENST00000451895|ENST00000451895.1|C9JWP2|PSG4 Number of predicted TMHs:  0
# ENST00000451895|ENST00000451895.1|C9JWP2|PSG4 Exp number of AAs in TMHs: 0.23777
# ENST00000451895|ENST00000451895.1|C9JWP2|PSG4 Exp number, first 60 AAs:  0.00057
# ENST00000451895|ENST00000451895.1|C9JWP2|PSG4 Total prob of N-in:        0.10852
ENST00000451895|ENST00000451895.1|C9JWP2|PSG4   TMHMM1.0        outside      1   252
```

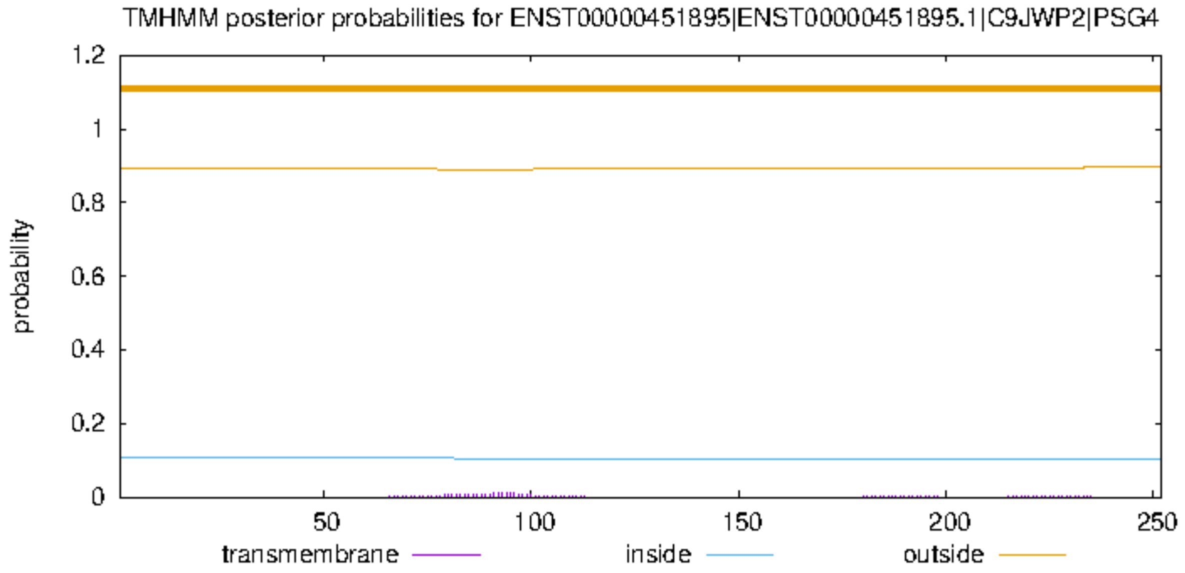

plot in postscript, script for making the plot in gnuplot, data for plot

---

```
# ENST00000187910|ENST00000187910.1||PSG6 Length: 424
# ENST00000187910|ENST00000187910.1||PSG6 Number of predicted TMHs:  0
# ENST00000187910|ENST00000187910.1||PSG6 Exp number of AAs in TMHs: 0.39944
# ENST00000187910|ENST00000187910.1||PSG6 Exp number, first 60 AAs:  0.26223
# ENST00000187910|ENST00000187910.1||PSG6 Total prob of N-in:        0.01234
ENST00000187910|ENST00000187910.1||PSG6 TMHMM1.0        outside      1   424
```

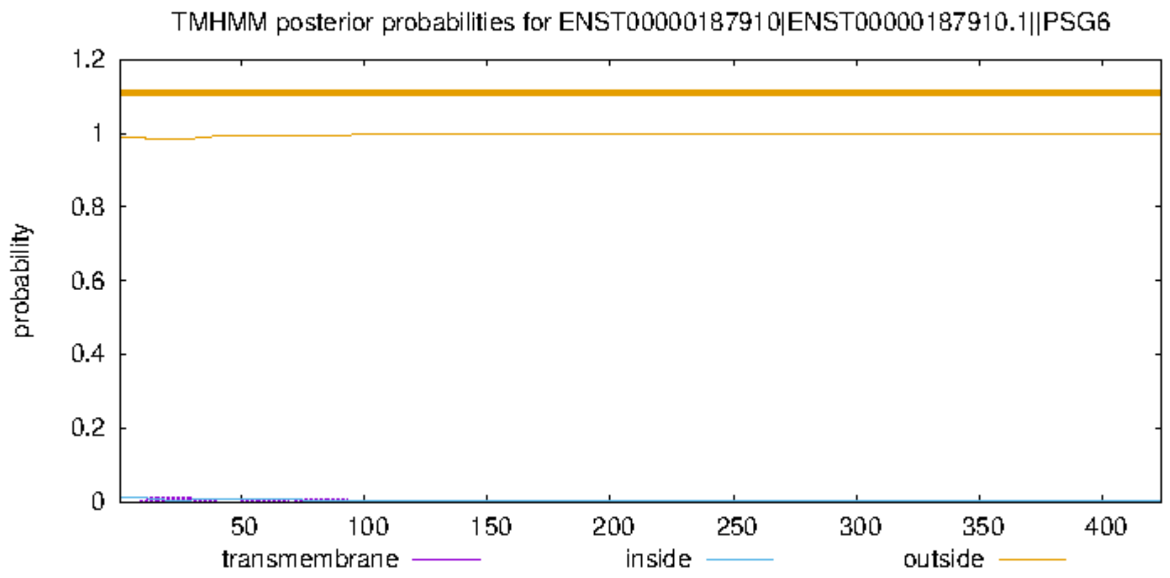

plot in postscript, script for making the plot in gnuplot, data for plot

---

```
# ENST00000402603|ENST00000402603.1|B5MCE1|PSG6 Length: 331
# ENST00000402603|ENST00000402603.1|B5MCE1|PSG6 Number of predicted TMHs:  0
# ENST00000402603|ENST00000402603.1|B5MCE1|PSG6 Exp number of AAs in TMHs: 0.46345
# ENST00000402603|ENST00000402603.1|B5MCE1|PSG6 Exp number, first 60 AAs:  0.30499
# ENST00000402603|ENST00000402603.1|B5MCE1|PSG6 Total prob of N-in:        0.01450
ENST00000402603|ENST00000402603.1|B5MCE1|PSG6   TMHMM1.0        outside      1   331
```

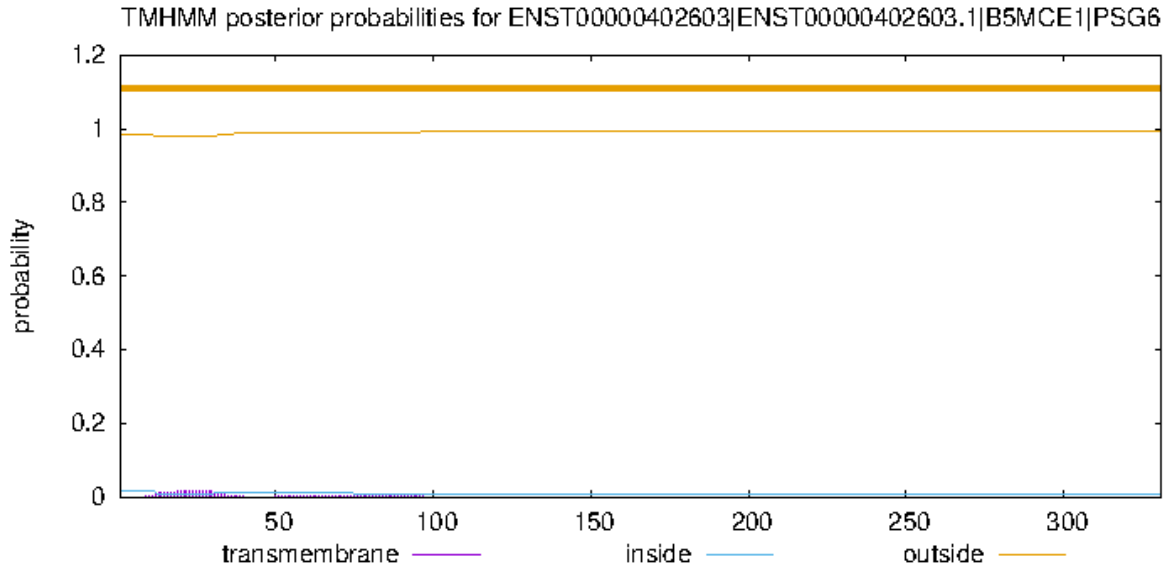

plot in postscript, script for making the plot in gnuplot, data for plot

---

```
# ENST00000244293|ENST00000244293.1||PSG9 Length: 402
# ENST00000244293|ENST00000244293.1||PSG9 Number of predicted TMHs:  0
# ENST00000244293|ENST00000244293.1||PSG9 Exp number of AAs in TMHs: 8.0099
# ENST00000244293|ENST00000244293.1||PSG9 Exp number, first 60 AAs:  0.0334
# ENST00000244293|ENST00000244293.1||PSG9 Total prob of N-in:        0.00976
ENST00000244293|ENST00000244293.1||PSG9 TMHMM1.0        outside      1   402
```

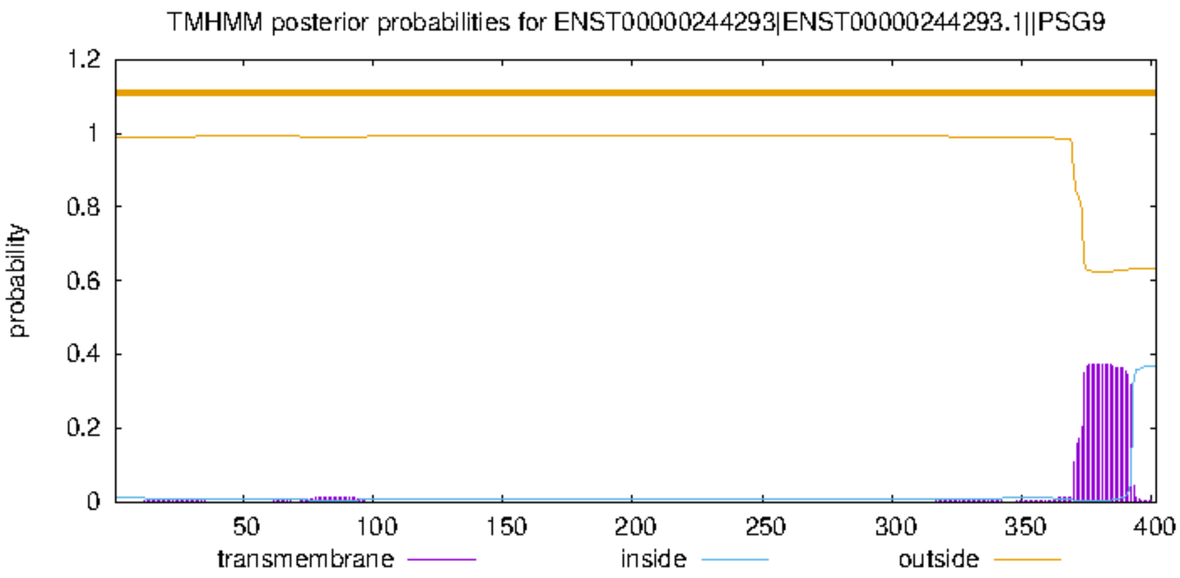

[plot](#) in postscript, [script](#) for making the plot in gnuplot, [data](#) for plot

---

```
# ENST00000405312|ENST00000405312.1||PSG4 Length: 419
# ENST00000405312|ENST00000405312.1||PSG4 Number of predicted TMHs:  0
# ENST00000405312|ENST00000405312.1||PSG4 Exp number of AAs in TMHs: 0.0685000000000001
# ENST00000405312|ENST00000405312.1||PSG4 Exp number, first 60 AAs:  0.04001
# ENST00000405312|ENST00000405312.1||PSG4 Total prob of N-in:        0.00347
ENST00000405312|ENST00000405312.1||PSG4 TMHMM1.0        outside      1   419
```

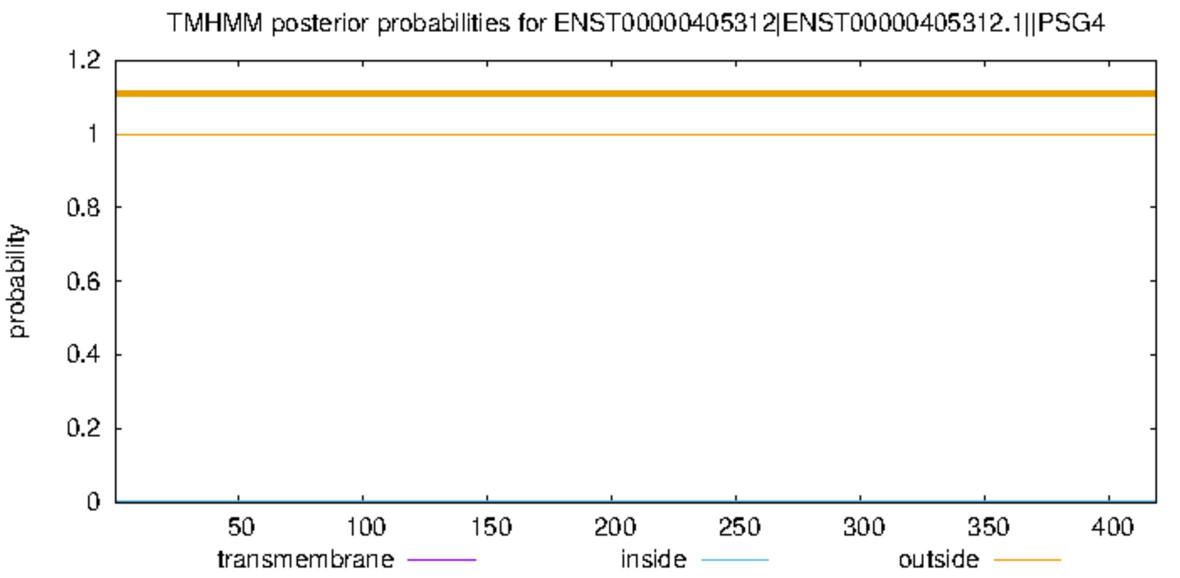

[plot](#) in postscript, [script](#) for making the plot in gnuplot, [data](#) for plot

---

```
# ENST00000418820|ENST00000418820.1|H7C1I4|PSG9 Length: 326
# ENST00000418820|ENST00000418820.1|H7C1I4|PSG9 Number of predicted TMHs:  0
# ENST00000418820|ENST00000418820.1|H7C1I4|PSG9 Exp number of AAs in TMHs: 0.24538
# ENST00000418820|ENST00000418820.1|H7C1I4|PSG9 Exp number, first 60 AAs:  0.03323
# ENST00000418820|ENST00000418820.1|H7C1I4|PSG9 Total prob of N-in:        0.00966
ENST00000418820|ENST00000418820.1|H7C1I4|PSG9   TMHMM1.0        outside      1   326
```

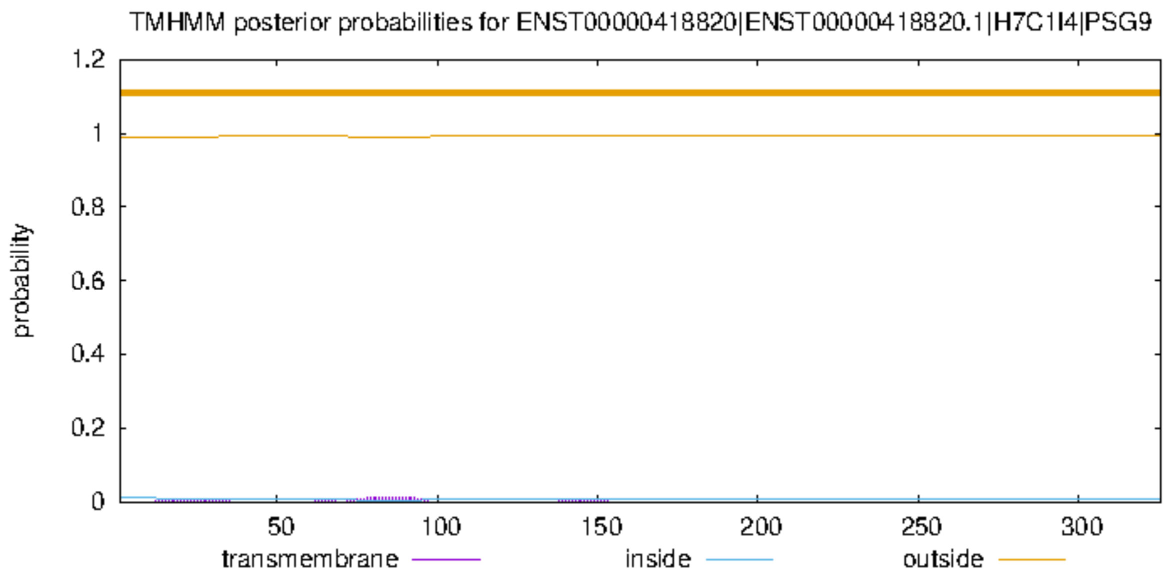

plot in postscript, script for making the plot in gnuplot, data for plot

---

```
# ENST00000404209|ENST00000404209.1||PSG8 Length: 419
# ENST00000404209|ENST00000404209.1||PSG8 Number of predicted TMHs:  0
# ENST00000404209|ENST00000404209.1||PSG8 Exp number of AAs in TMHs: 0.0702899999999999
# ENST00000404209|ENST00000404209.1||PSG8 Exp number, first 60 AAs:  0.02924
# ENST00000404209|ENST00000404209.1||PSG8 Total prob of N-in:        0.00370
ENST00000404209|ENST00000404209.1||PSG8 TMHMM1.0        outside      1   419
```

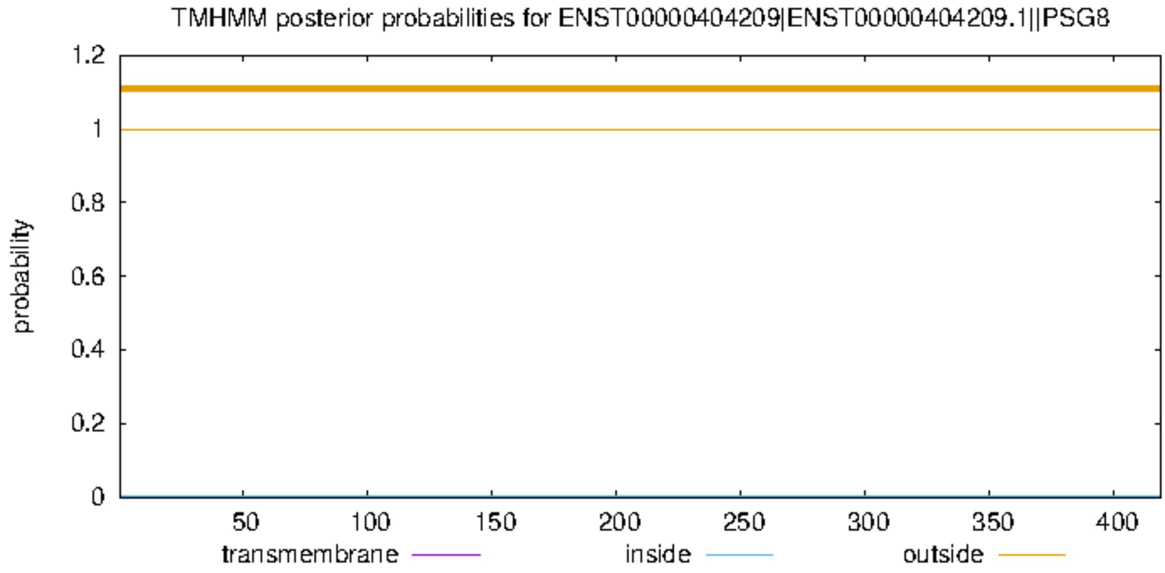

plot in postscript, script for making the plot in gnuplot, data for plot

---

```
# ENST00000406636|ENST00000406636.1||PSG8 Length: 297
# ENST00000406636|ENST00000406636.1||PSG8 Number of predicted TMHs:  0
# ENST00000406636|ENST00000406636.1||PSG8 Exp number of AAs in TMHs: 0.000440000000000001
# ENST00000406636|ENST00000406636.1||PSG8 Exp number, first 60 AAs:  0
# ENST00000406636|ENST00000406636.1||PSG8 Total prob of N-in:        0.00335
ENST00000406636|ENST00000406636.1||PSG8 TMHMM1.0        outside      1   297
```

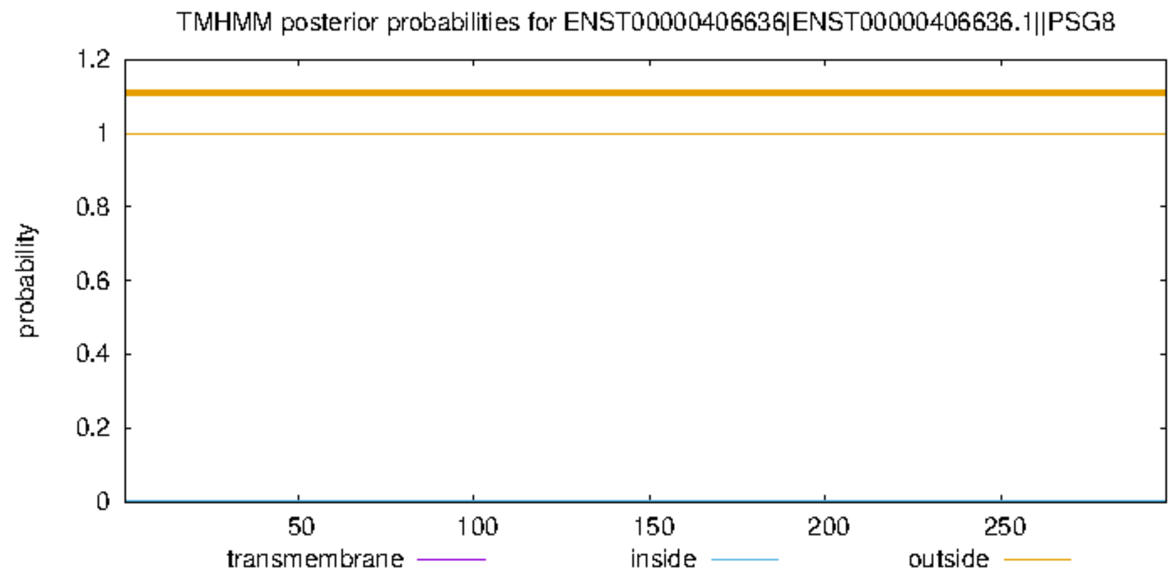

plot in postscript, script for making the plot in gnuplot, data for plot

---

```
# ENST00000403380|ENST00000403380.1|G5E9F7|PSG1 Length: 326
# ENST00000403380|ENST00000403380.1|G5E9F7|PSG1 Number of predicted TMHs:  0
# ENST00000403380|ENST00000403380.1|G5E9F7|PSG1 Exp number of AAs in TMHs: 0.21848
# ENST00000403380|ENST00000403380.1|G5E9F7|PSG1 Exp number, first 60 AAs:  0.12906
# ENST00000403380|ENST00000403380.1|G5E9F7|PSG1 Total prob of N-in:        0.01493
ENST00000403380|ENST00000403380.1|G5E9F7|PSG1   TMHMM1.0        outside      1   326
```

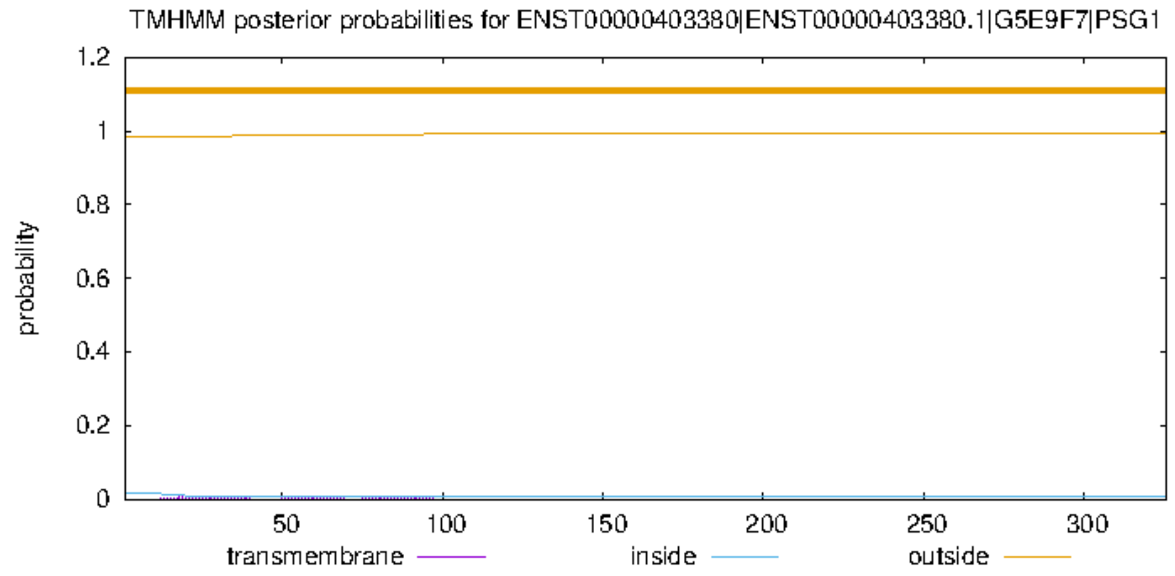

plot in postscript, script for making the plot in gnuplot, data for plot

---

```
# ENST00000593948|ENST00000593948.1|M0R0U8|PSG9 Length: 333
# ENST00000593948|ENST00000593948.1|M0R0U8|PSG9 Number of predicted TMHs:  0
# ENST00000593948|ENST00000593948.1|M0R0U8|PSG9 Exp number of AAs in TMHs: 0.23138
# ENST00000593948|ENST00000593948.1|M0R0U8|PSG9 Exp number, first 60 AAs:  0.03278
# ENST00000593948|ENST00000593948.1|M0R0U8|PSG9 Total prob of N-in:        0.00932
ENST00000593948|ENST00000593948.1|M0R0U8|PSG9   TMHMM1.0        outside      1   333
```

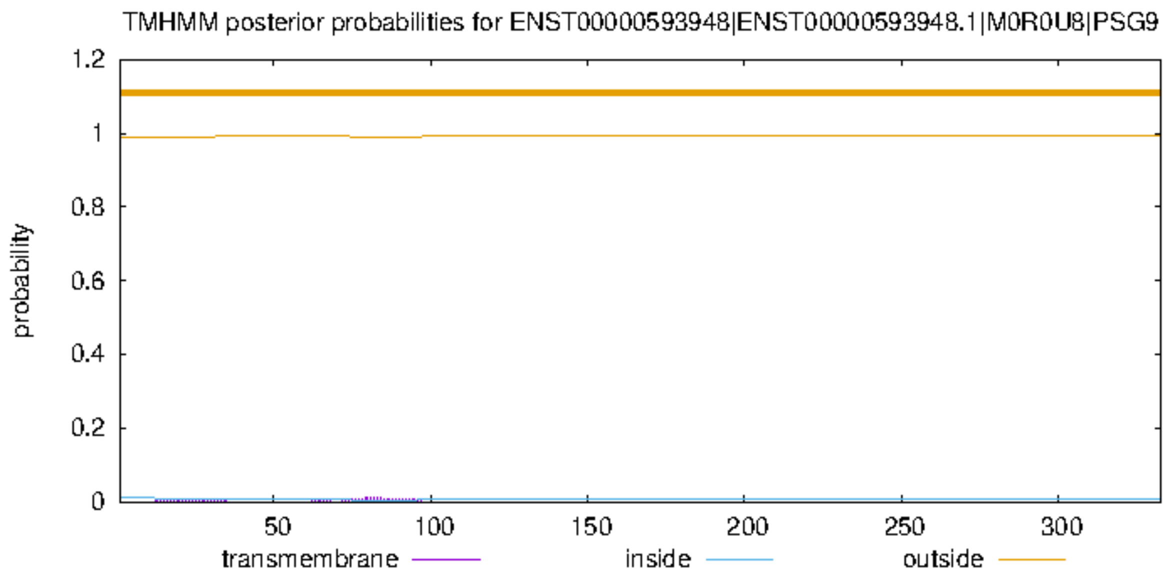

plot in postscript, script for making the plot in gnuplot, data for plot

---

```
# ENST00000594375|ENST00000594375.1|M0QZA7|PSG6 Length: 158
# ENST00000594375|ENST00000594375.1|M0QZA7|PSG6 Number of predicted TMHs:  0
# ENST00000594375|ENST00000594375.1|M0QZA7|PSG6 Exp number of AAs in TMHs: 0.00274
# ENST00000594375|ENST00000594375.1|M0QZA7|PSG6 Exp number, first 60 AAs:  0.00025
# ENST00000594375|ENST00000594375.1|M0QZA7|PSG6 Total prob of N-in:        0.02761
ENST00000594375|ENST00000594375.1|M0QZA7|PSG6	TMHMM1.0	outside	     1   158
```

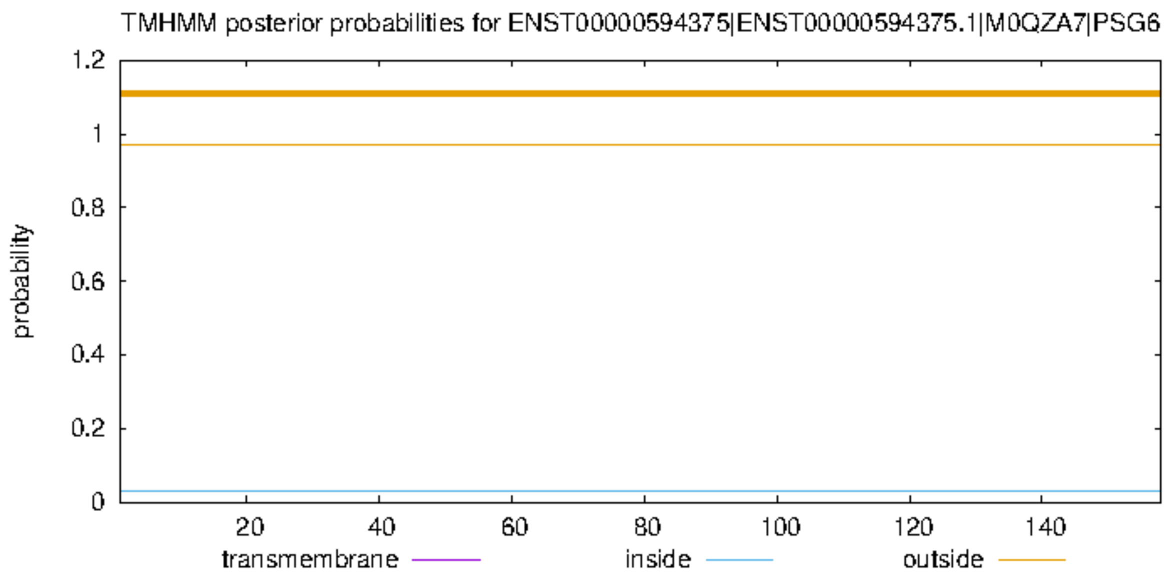

plot in postscript, script for making the plot in gnuplot, data for plot

---

```
# ENST00000406070|ENST00000406070.1||PSG7 Length: 419
# ENST00000406070|ENST00000406070.1||PSG7 Number of predicted TMHs:  0
# ENST00000406070|ENST00000406070.1||PSG7 Exp number of AAs in TMHs: 0.0226
# ENST00000406070|ENST00000406070.1||PSG7 Exp number, first 60 AAs:  0.02062
# ENST00000406070|ENST00000406070.1||PSG7 Total prob of N-in:        0.00184
ENST00000406070|ENST00000406070.1||PSG7	TMHMM1.0	outside	     1   419
```

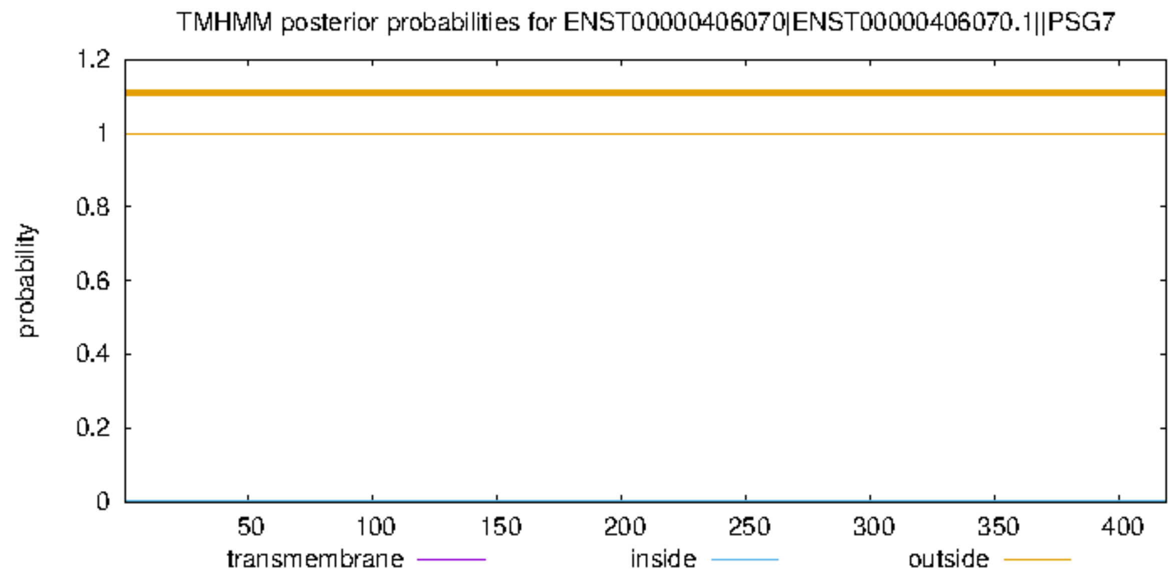

plot in postscript, script for making the plot in gnuplot, data for plot

---

```
# ENST00000406487|ENST00000406487.1||PSG2 Length: 335
# ENST00000406487|ENST00000406487.1||PSG2 Number of predicted TMHs:  0
# ENST00000406487|ENST00000406487.1||PSG2 Exp number of AAs in TMHs: 0.0828099999999999
# ENST00000406487|ENST00000406487.1||PSG2 Exp number, first 60 AAs:  0.0294
# ENST00000406487|ENST00000406487.1||PSG2 Total prob of N-in:        0.00570
ENST00000406487|ENST00000406487.1||PSG2 TMHMM1.0        outside      1   335
```

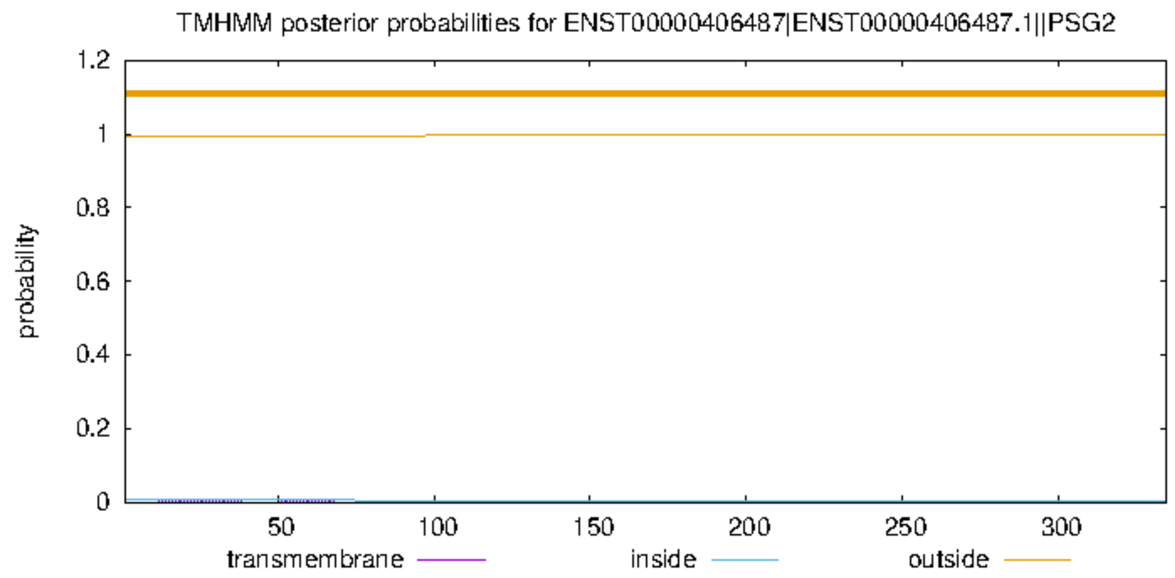

plot in postscript, script for making the plot in gnuplot, data for plot

---

```
# ENST00000327495|ENST00000327495.1||PSG3 Length: 428
# ENST00000327495|ENST00000327495.1||PSG3 Number of predicted TMHs:  0
# ENST00000327495|ENST00000327495.1||PSG3 Exp number of AAs in TMHs: 1.62508
# ENST00000327495|ENST00000327495.1||PSG3 Exp number, first 60 AAs:  1.30639
# ENST00000327495|ENST00000327495.1||PSG3 Total prob of N-in:        0.05873
ENST00000327495|ENST00000327495.1||PSG3 TMHMM1.0        outside      1   428
```

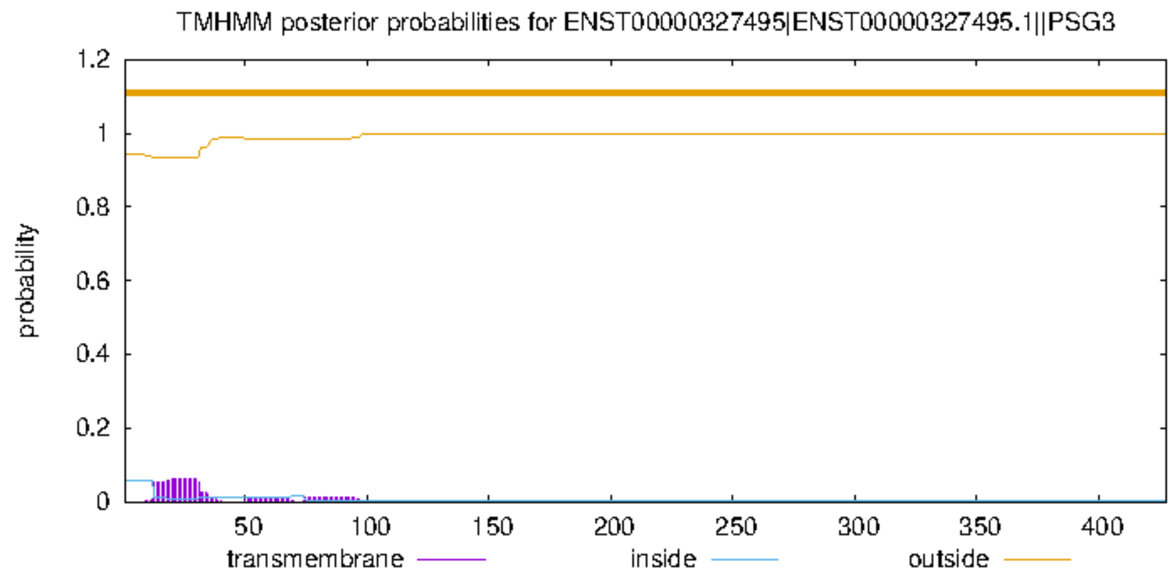

[plot](#) in postscript, [script](#) for making the plot in gnuplot, [data](#) for plot

---

```
# ENST00000320078|ENST00000320078.1||PSG11 Length: 335
# ENST00000320078|ENST00000320078.1||PSG11 Number of predicted TMHs:  0
# ENST00000320078|ENST00000320078.1||PSG11 Exp number of AAs in TMHs: 0.46121
# ENST00000320078|ENST00000320078.1||PSG11 Exp number, first 60 AAs:  0.35354
# ENST00000320078|ENST00000320078.1||PSG11 Total prob of N-in:        0.01790
ENST00000320078|ENST00000320078.1||PSG11        TMHMM1.0        outside      1   335
```

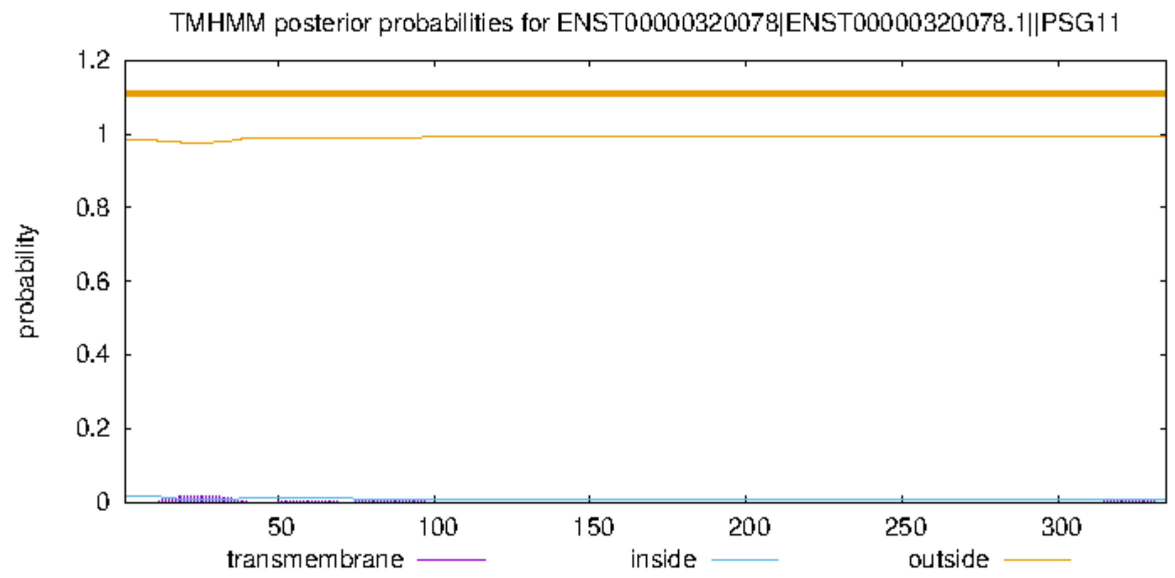

[plot](#) in postscript, [script](#) for making the plot in gnuplot, [data](#) for plot

---

```
# ENST00000407568|ENST00000407568.1|E9PC55|PSG5 Length: 157
# ENST00000407568|ENST00000407568.1|E9PC55|PSG5 Number of predicted TMHs:  0
# ENST00000407568|ENST00000407568.1|E9PC55|PSG5 Exp number of AAs in TMHs: 1.23865
# ENST00000407568|ENST00000407568.1|E9PC55|PSG5 Exp number, first 60 AAs:  1.01076
# ENST00000407568|ENST00000407568.1|E9PC55|PSG5 Total prob of N-in:        0.04826
ENST00000407568|ENST00000407568.1|E9PC55|PSG5   TMHMM1.0        outside      1   157
```

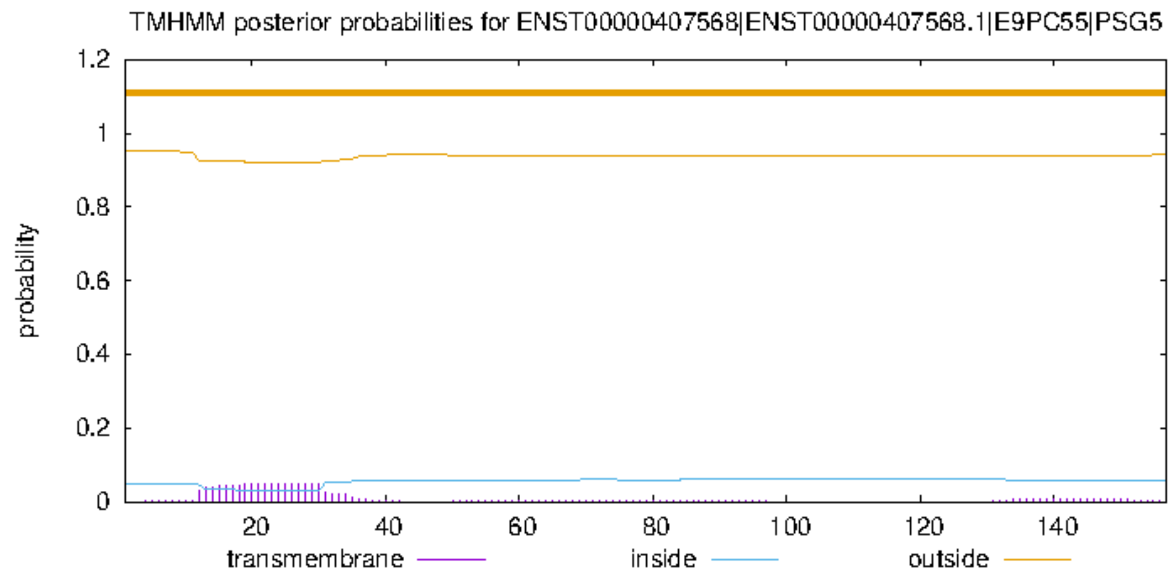

plot in postscript, script for making the plot in gnuplot, data for plot

---

```
# ENST00000596907|ENST00000596907.1|M0R0B3|PSG4 Length: 292
# ENST00000596907|ENST00000596907.1|M0R0B3|PSG4 Number of predicted TMHs:  0
# ENST00000596907|ENST00000596907.1|M0R0B3|PSG4 Exp number of AAs in TMHs: 0.00405
# ENST00000596907|ENST00000596907.1|M0R0B3|PSG4 Exp number, first 60 AAs:  0.00266
# ENST00000596907|ENST00000596907.1|M0R0B3|PSG4 Total prob of N-in:        0.00548
ENST00000596907|ENST00000596907.1|M0R0B3|PSG4   TMHMM1.0        outside      1   292
```

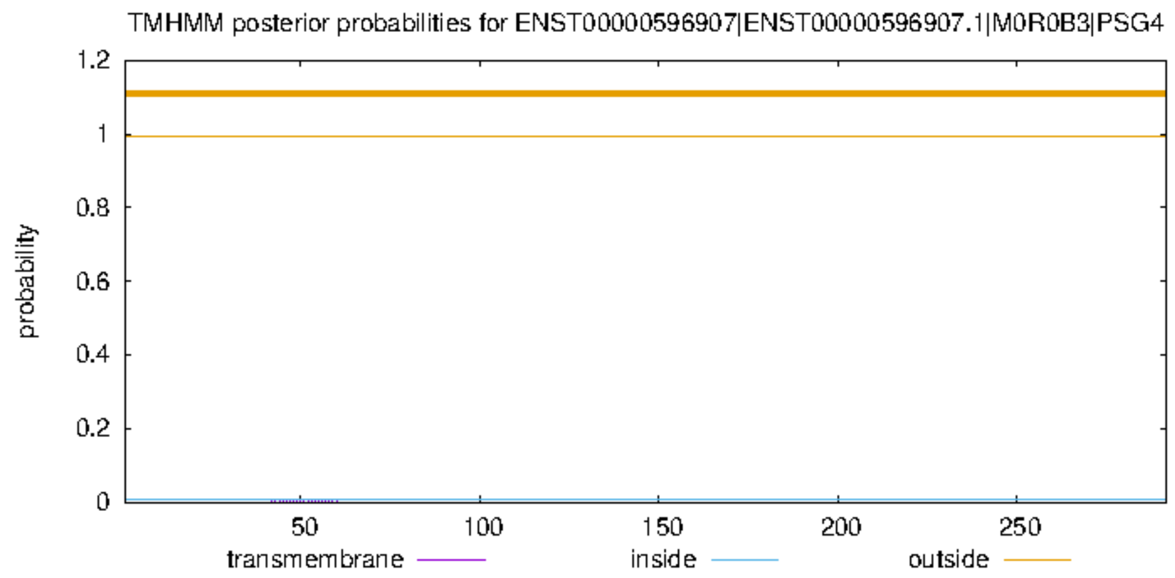

plot in postscript, script for making the plot in gnuplot, data for plot

---

```
# ENST00000599753|ENST00000599753.1|M0R314|PSG6 Length: 81
# ENST00000599753|ENST00000599753.1|M0R314|PSG6 Number of predicted TMHs:  0
# ENST00000599753|ENST00000599753.1|M0R314|PSG6 Exp number of AAs in TMHs: 0.00868999999999999
# ENST00000599753|ENST00000599753.1|M0R314|PSG6 Exp number, first 60 AAs:  0.00866
# ENST00000599753|ENST00000599753.1|M0R314|PSG6 Total prob of N-in:        0.23322
ENST00000599753|ENST00000599753.1|M0R314|PSG6   TMHMM1.0        outside      1    81
```

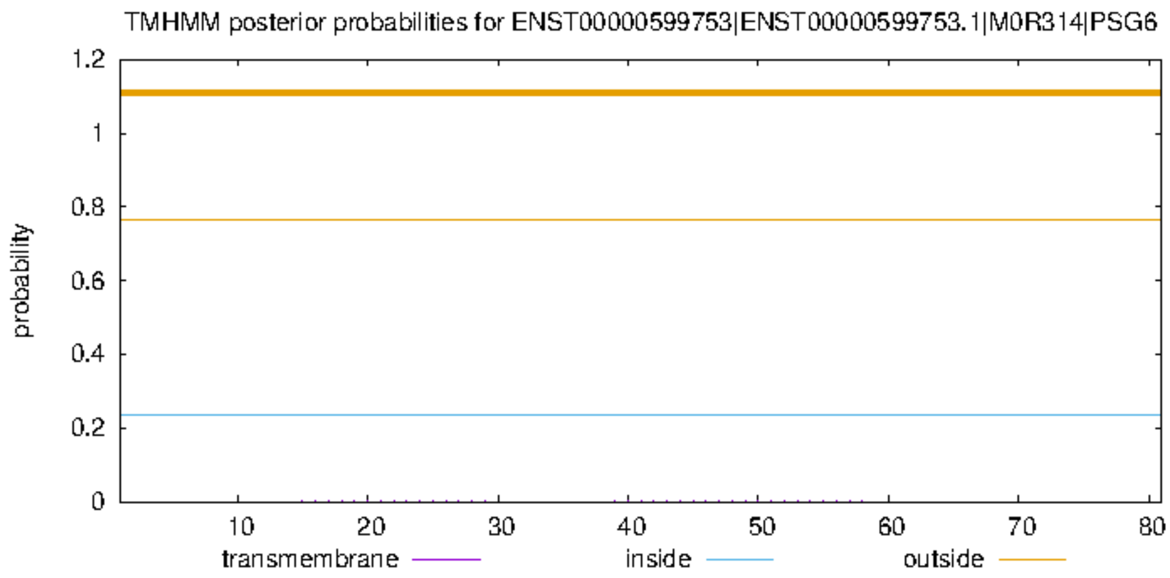

plot in postscript, script for making the plot in gnuplot, data for plot

---

```
# ENST00000601833|ENST00000601833.1|M0QXW0|PSG6 Length: 73
# ENST00000601833|ENST00000601833.1|M0QXW0|PSG6 Number of predicted TMHs:  0
# ENST00000601833|ENST00000601833.1|M0QXW0|PSG6 Exp number of AAs in TMHs: 2.27972
# ENST00000601833|ENST00000601833.1|M0QXW0|PSG6 Exp number, first 60 AAs:  2.27208
# ENST00000601833|ENST00000601833.1|M0QXW0|PSG6 Total prob of N-in:        0.40787
ENST00000601833|ENST00000601833.1|M0QXW0|PSG6   TMHMM1.0        outside      1    73
```

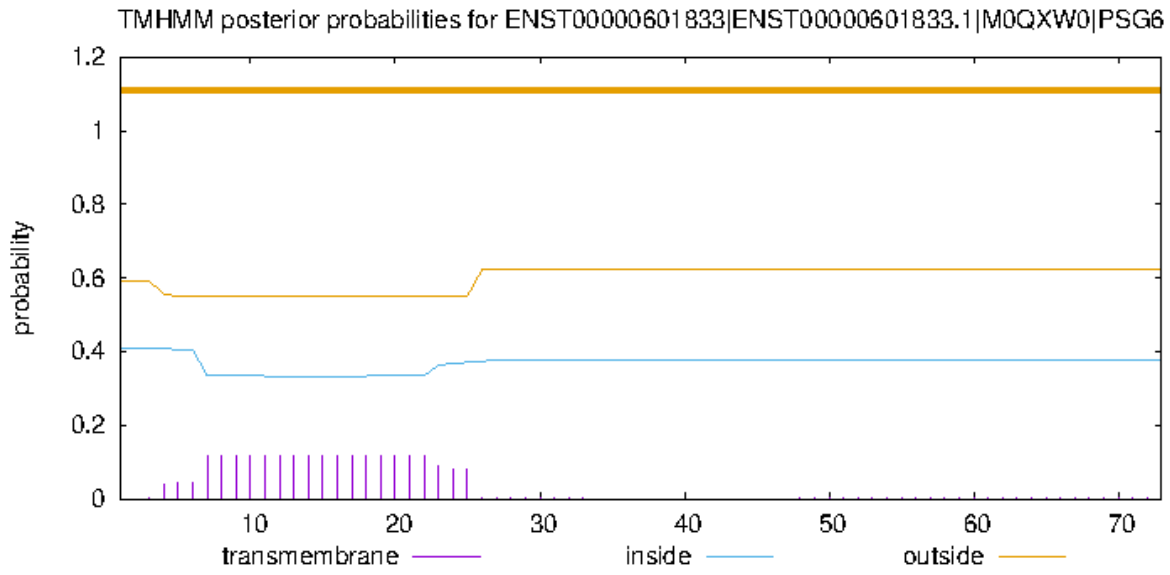

plot in postscript, script for making the plot in gnuplot, data for plot

---

```
# ENST00000599746|ENST00000599746.1|M0QY31|PSG4 Length: 244
# ENST00000599746|ENST00000599746.1|M0QY31|PSG4 Number of predicted TMHs:  0
# ENST00000599746|ENST00000599746.1|M0QY31|PSG4 Exp number of AAs in TMHs: 0.3678
# ENST00000599746|ENST00000599746.1|M0QY31|PSG4 Exp number, first 60 AAs:  0.31148
# ENST00000599746|ENST00000599746.1|M0QY31|PSG4 Total prob of N-in:        0.00664
ENST00000599746|ENST00000599746.1|M0QY31|PSG4   TMHMM1.0        outside      1   244
```

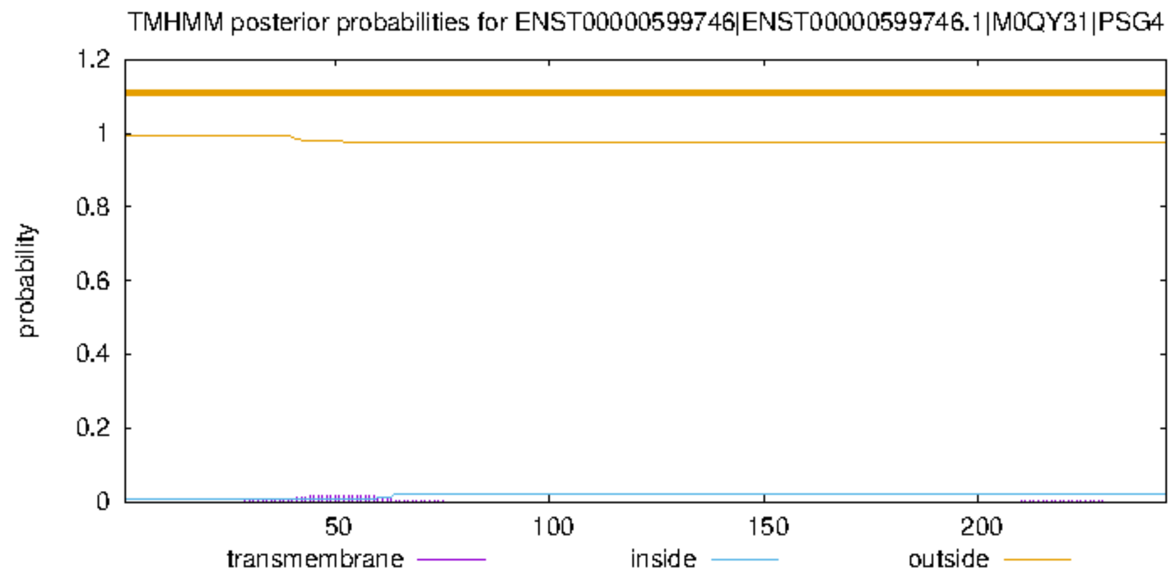

plot in postscript, script for making the plot in gnuplot, data for plot

---

```
# ENST00000595930|ENST00000595930.1|M0QY44|PSG1 Length: 154
# ENST00000595930|ENST00000595930.1|M0QY44|PSG1 Number of predicted TMHs:  0
# ENST00000595930|ENST00000595930.1|M0QY44|PSG1 Exp number of AAs in TMHs: 0.23988
# ENST00000595930|ENST00000595930.1|M0QY44|PSG1 Exp number, first 60 AAs:  0.2387
# ENST00000595930|ENST00000595930.1|M0QY44|PSG1 Total prob of N-in:        0.23690
ENST00000595930|ENST00000595930.1|M0QY44|PSG1   TMHMM1.0        outside      1   154
```

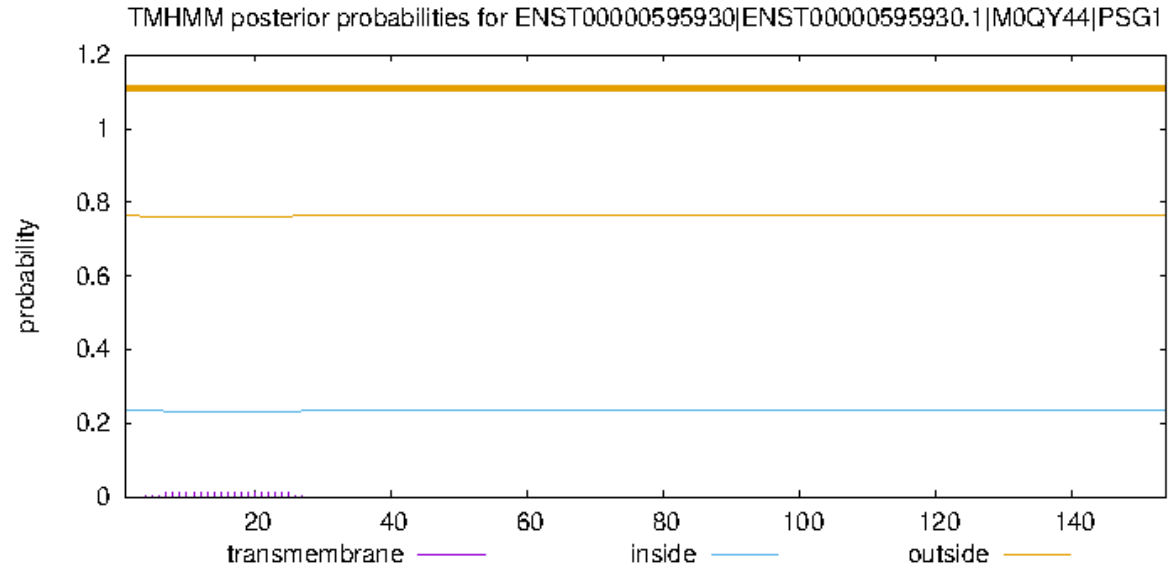

plot in postscript, script for making the plot in gnuplot, data for plot

---

```
# ENST00000595356|ENST00000595356.1||PSG1 Length: 417
# ENST00000595356|ENST00000595356.1||PSG1 Number of predicted TMHs:  0
# ENST00000595356|ENST00000595356.1||PSG1 Exp number of AAs in TMHs: 0.20816
# ENST00000595356|ENST00000595356.1||PSG1 Exp number, first 60 AAs:  0.12428
# ENST00000595356|ENST00000595356.1||PSG1 Total prob of N-in:        0.01115
ENST00000595356|ENST00000595356.1||PSG1 TMHMM1.0        outside      1   417
```

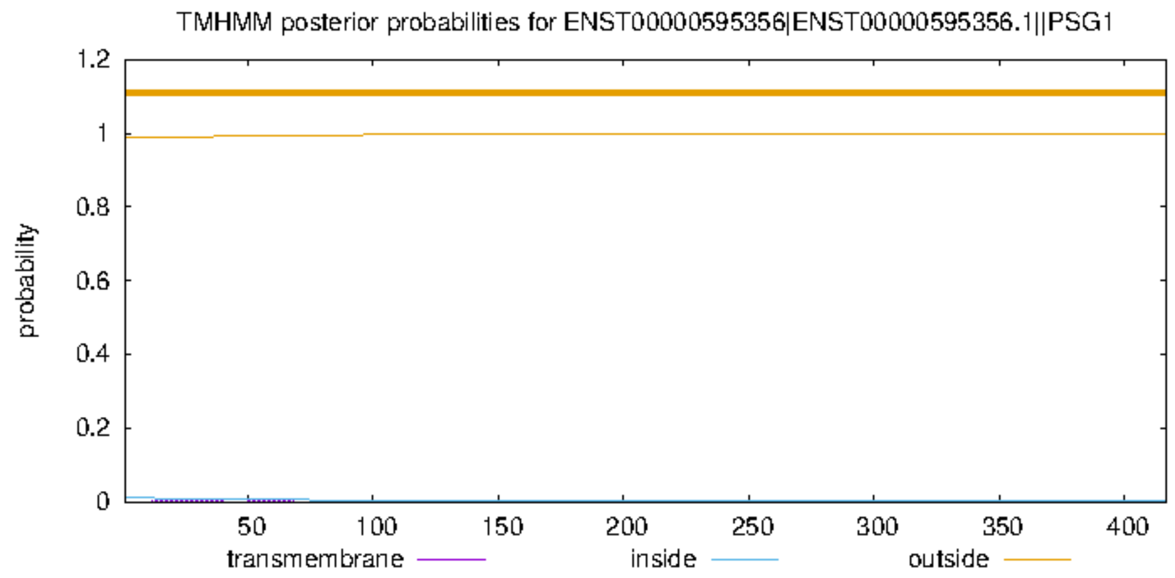

plot in postscript, script for making the plot in gnuplot, data for plot

---

```
# ENST00000595124|ENST00000595124.1|M0R235|PSG1 Length: 324
# ENST00000595124|ENST00000595124.1|M0R235|PSG1 Number of predicted TMHs:  0
# ENST00000595124|ENST00000595124.1|M0R235|PSG1 Exp number of AAs in TMHs: 0.21881
# ENST00000595124|ENST00000595124.1|M0R235|PSG1 Exp number, first 60 AAs:  0.12927
# ENST00000595124|ENST00000595124.1|M0R235|PSG1 Total prob of N-in:        0.01502
ENST00000595124|ENST00000595124.1|M0R235|PSG1   TMHMM1.0        outside      1   324
```

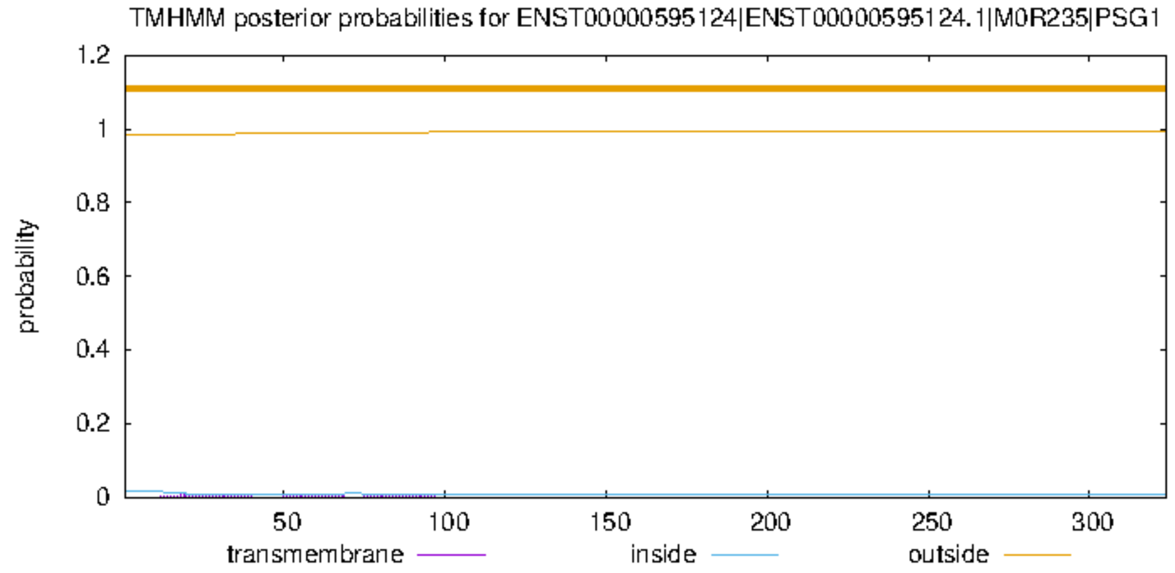

plot in postscript, script for making the plot in gnuplot, data for plot

---

```
# ENST00000596730|ENST00000596730.1|M0R0E4|PSG9 Length: 309
# ENST00000596730|ENST00000596730.1|M0R0E4|PSG9 Number of predicted TMHs:  0
# ENST00000596730|ENST00000596730.1|M0R0E4|PSG9 Exp number of AAs in TMHs: 8.47667
# ENST00000596730|ENST00000596730.1|M0R0E4|PSG9 Exp number, first 60 AAs:  0.04501
# ENST00000596730|ENST00000596730.1|M0R0E4|PSG9 Total prob of N-in:        0.01803
ENST00000596730|ENST00000596730.1|M0R0E4|PSG9   TMHMM1.0        outside      1   309
```

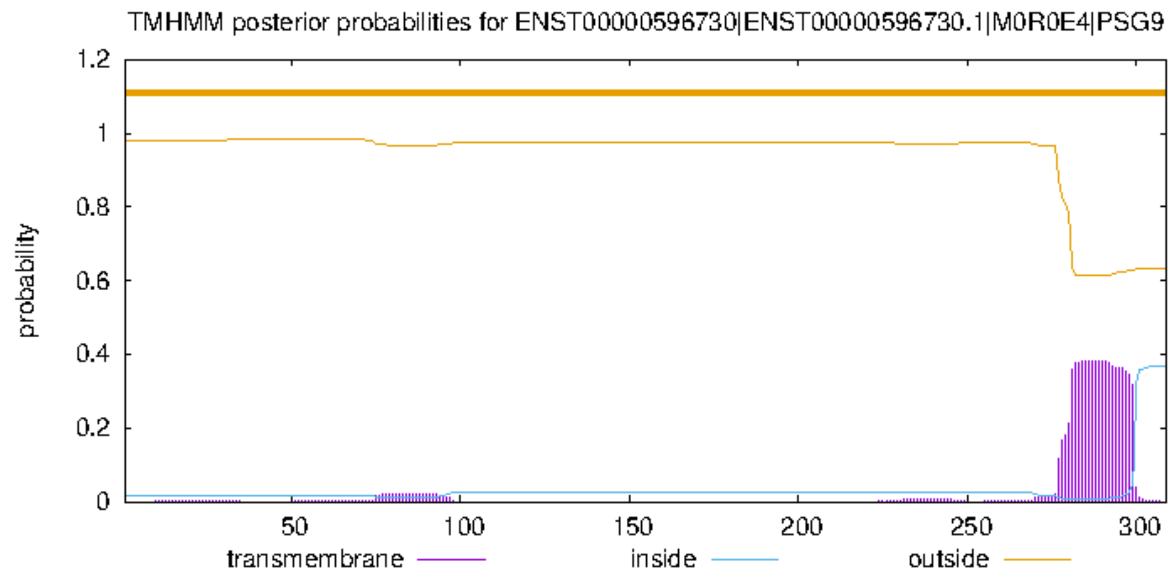

plot in postscript, script for making the plot in gnuplot, data for plot

---

```
# ENST00000597058|ENST00000597058.1|M0QZQ1|PSG1 Length: 242
# ENST00000597058|ENST00000597058.1|M0QZQ1|PSG1 Number of predicted TMHs:  0
# ENST00000597058|ENST00000597058.1|M0QZQ1|PSG1 Exp number of AAs in TMHs: 0.00249
# ENST00000597058|ENST00000597058.1|M0QZQ1|PSG1 Exp number, first 60 AAs:  0.00212
# ENST00000597058|ENST00000597058.1|M0QZQ1|PSG1 Total prob of N-in:        0.04317
ENST00000597058|ENST00000597058.1|M0QZQ1|PSG1   TMHMM1.0        outside      1   242
```

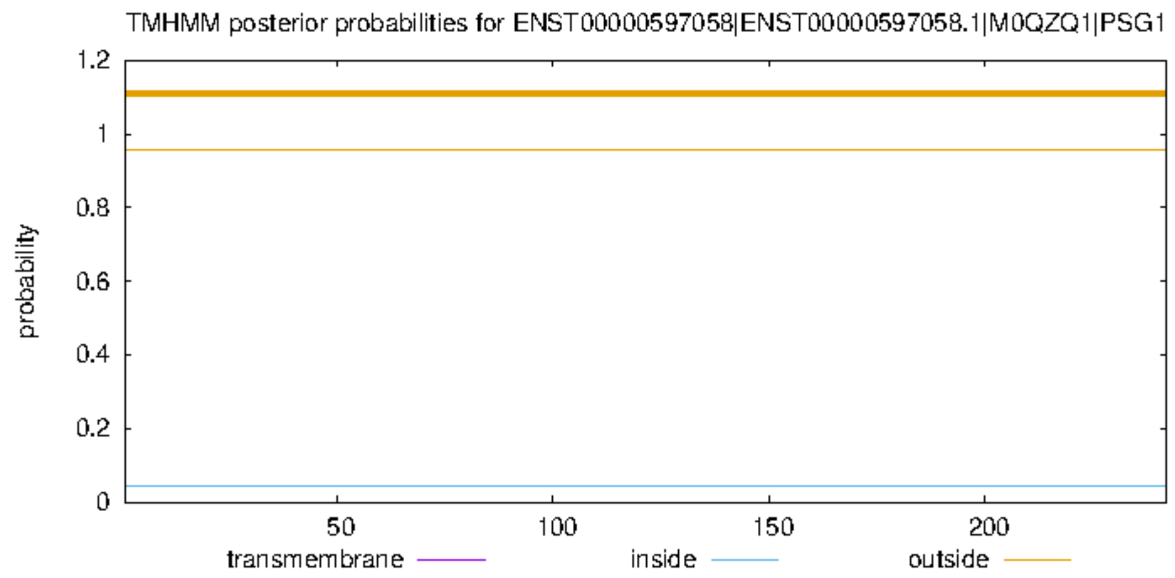

plot in postscript, script for making the plot in gnuplot, data for plot

---

```
# ENST00000597374|ENST00000597374.1|M0R2A2|PSG4 Length: 258
# ENST00000597374|ENST00000597374.1|M0R2A2|PSG4 Number of predicted TMHs:  0
# ENST00000597374|ENST00000597374.1|M0R2A2|PSG4 Exp number of AAs in TMHs: 0.00335
# ENST00000597374|ENST00000597374.1|M0R2A2|PSG4 Exp number, first 60 AAs:  1e-05
# ENST00000597374|ENST00000597374.1|M0R2A2|PSG4 Total prob of N-in:        0.03559
ENST00000597374|ENST00000597374.1|M0R2A2|PSG4   TMHMM1.0        outside      1   258
```

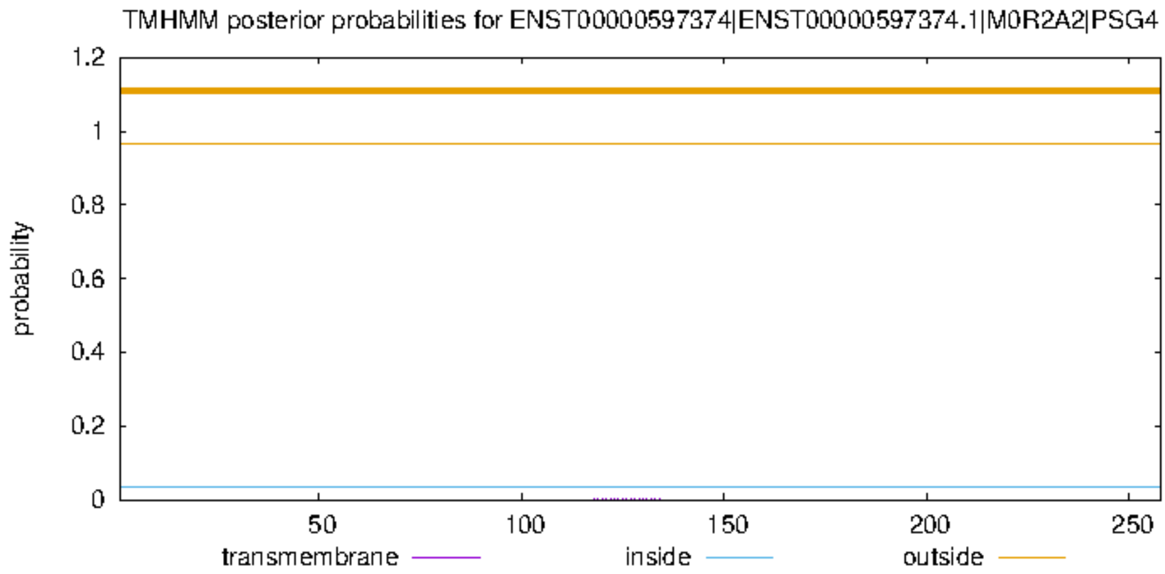

plot in postscript, script for making the plot in gnuplot, data for plot

---

```
# ENST00000600572|ENST00000600572.1|M0QYU2|PSG4 Length: 78
# ENST00000600572|ENST00000600572.1|M0QYU2|PSG4 Number of predicted TMHs:  0
# ENST00000600572|ENST00000600572.1|M0QYU2|PSG4 Exp number of AAs in TMHs: 0.0371800000000001
# ENST00000600572|ENST00000600572.1|M0QYU2|PSG4 Exp number, first 60 AAs:  0.0336700000000001
# ENST00000600572|ENST00000600572.1|M0QYU2|PSG4 Total prob of N-in:        0.40649
ENST00000600572|ENST00000600572.1|M0QYU2|PSG4   TMHMM1.0        outside      1    78
```

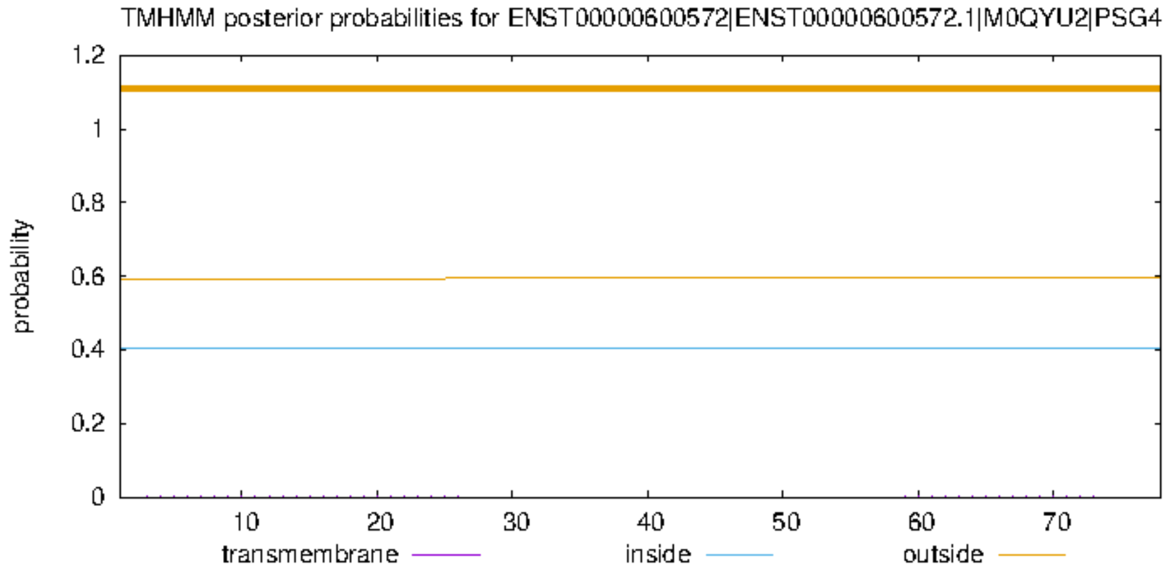

plot in postscript, script for making the plot in gnuplot, data for plot

---

```
# ENST00000599812|ENST00000599812.1|M0R1G9|PSG5 Length: 428
# ENST00000599812|ENST00000599812.1|M0R1G9|PSG5 Number of predicted TMHs:  0
# ENST00000599812|ENST00000599812.1|M0R1G9|PSG5 Exp number of AAs in TMHs: 0.420740000000001
# ENST00000599812|ENST00000599812.1|M0R1G9|PSG5 Exp number, first 60 AAs:  0.40304
# ENST00000599812|ENST00000599812.1|M0R1G9|PSG5 Total prob of N-in:        0.01852
ENST00000599812|ENST00000599812.1|M0R1G9|PSG5   TMHMM1.0        outside      1   428
```

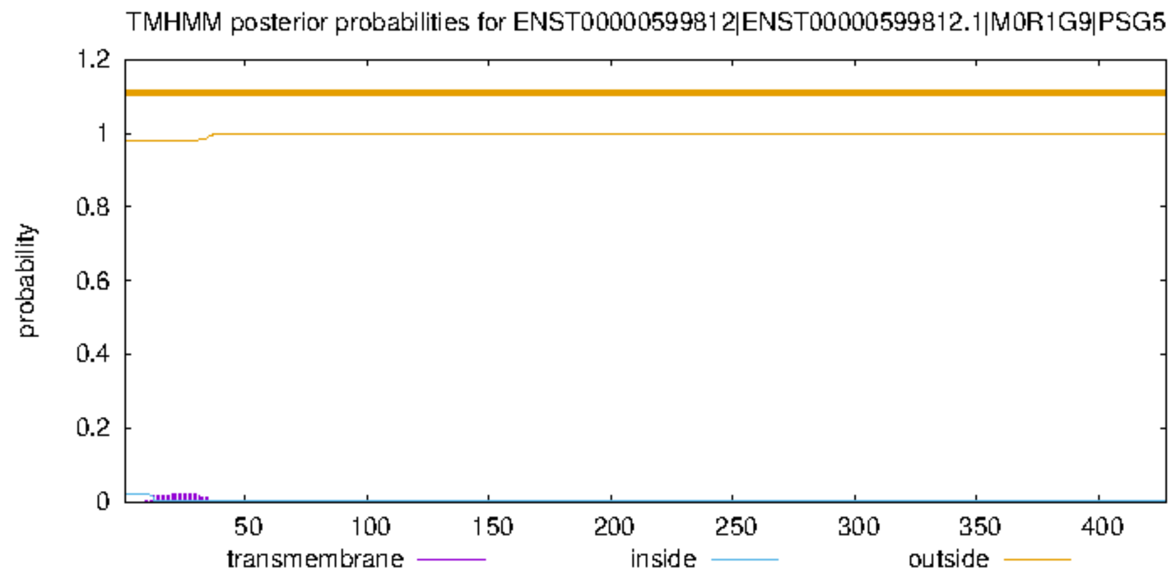

plot in postscript, script for making the plot in gnuplot, data for plot

---

```
# ENST00000598133|ENST00000598133.1|M0R276|PSG11 Length: 335
# ENST00000598133|ENST00000598133.1|M0R276|PSG11 Number of predicted TMHs:  0
# ENST00000598133|ENST00000598133.1|M0R276|PSG11 Exp number of AAs in TMHs: 0.598269999999999
# ENST00000598133|ENST00000598133.1|M0R276|PSG11 Exp number, first 60 AAs:  0.38512
# ENST00000598133|ENST00000598133.1|M0R276|PSG11 Total prob of N-in:        0.01963
ENST00000598133|ENST00000598133.1|M0R276|PSG11  TMHMM1.0        outside      1   335
```

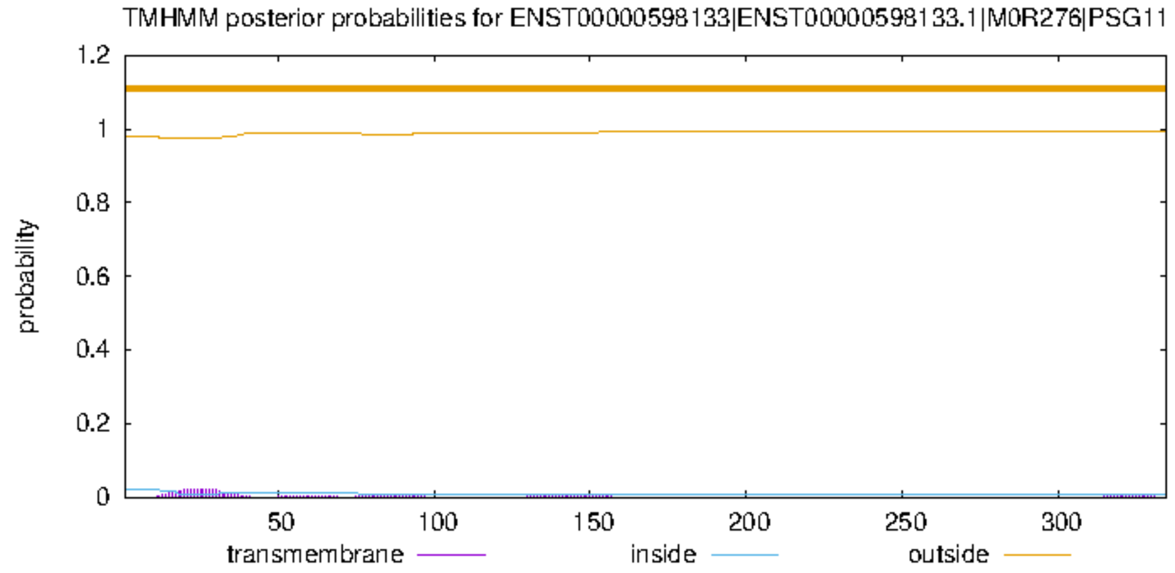

plot in postscript, script for making the plot in gnuplot, data for plot

---

```
# ENST00000621109|ENST00000621109.1|A0A087WYK1|PSG9 Length: 419
# ENST00000621109|ENST00000621109.1|A0A087WYK1|PSG9 Number of predicted TMHs:  0
# ENST00000621109|ENST00000621109.1|A0A087WYK1|PSG9 Exp number of AAs in TMHs: 0.171590000000001
# ENST00000621109|ENST00000621109.1|A0A087WYK1|PSG9 Exp number, first 60 AAs:  0.03001
# ENST00000621109|ENST00000621109.1|A0A087WYK1|PSG9 Total prob of N-in:        0.00738
ENST00000621109|ENST00000621109.1|A0A087WYK1|PSG9       TMHMM1.0        outside      1   419
```

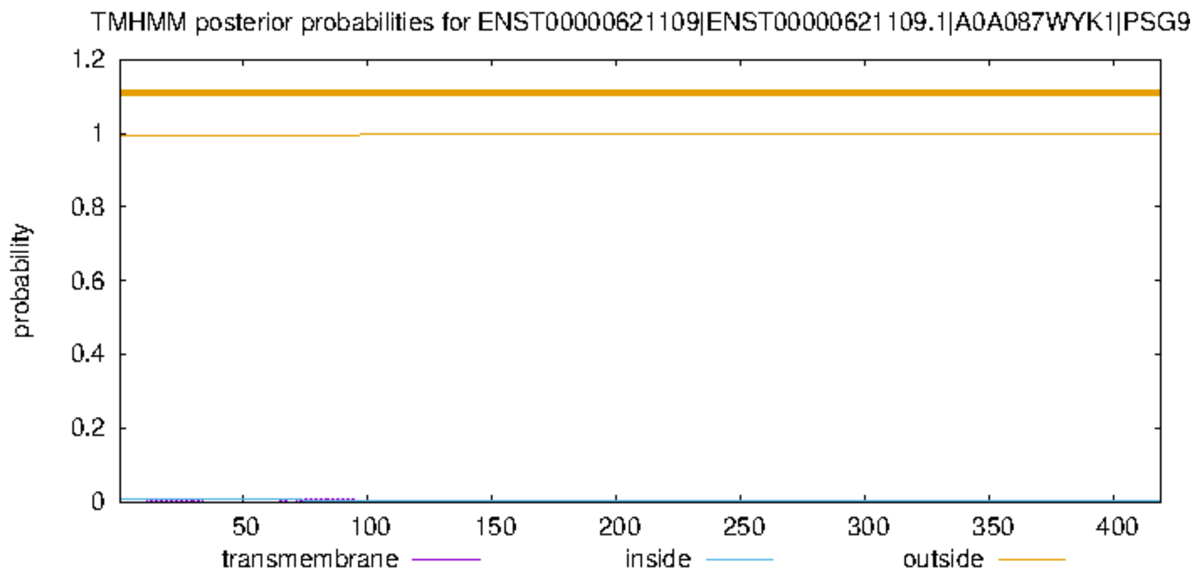

plot in postscript, script for making the plot in gnuplot, data for plot

---

```
# ENST00000614582|ENST00000614582.1||PSG3 Length: 428
# ENST00000614582|ENST00000614582.1||PSG3 Number of predicted TMHs:  0
# ENST00000614582|ENST00000614582.1||PSG3 Exp number of AAs in TMHs: 1.62508
# ENST00000614582|ENST00000614582.1||PSG3 Exp number, first 60 AAs:  1.30639
# ENST00000614582|ENST00000614582.1||PSG3 Total prob of N-in:        0.05873
ENST00000614582|ENST00000614582.1||PSG3 TMHMM1.0        outside      1   428
```

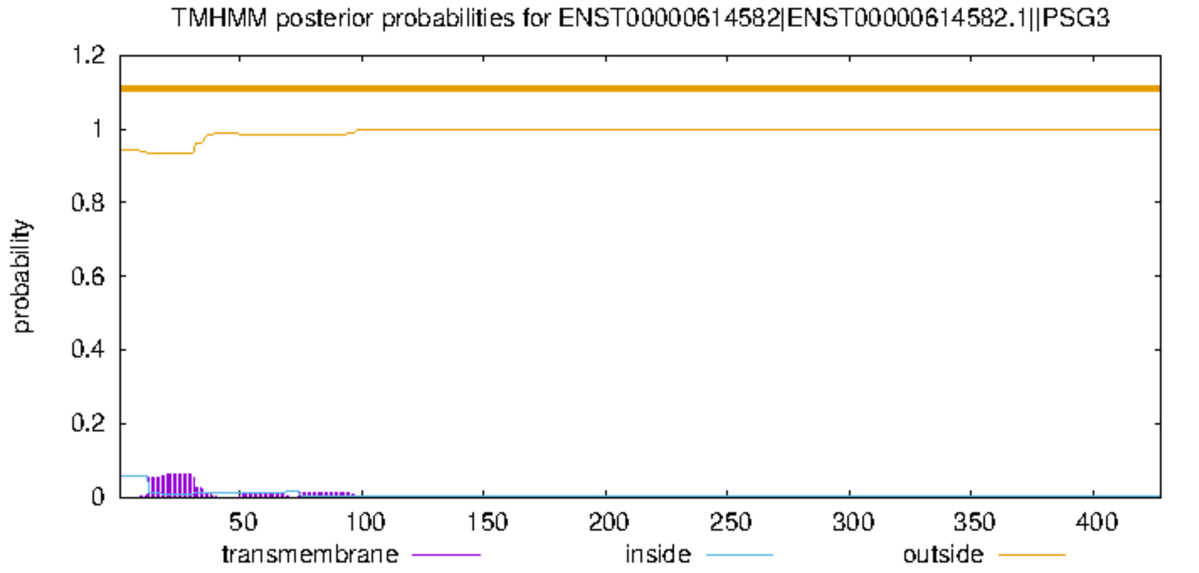

plot in postscript, script for making the plot in gnuplot, data for plot

---

```
# ENST00000599371|ENST00000599371.1|M0R2U8|PSG4 Length: 96
# ENST00000599371|ENST00000599371.1|M0R2U8|PSG4 Number of predicted TMHs:  0
# ENST00000599371|ENST00000599371.1|M0R2U8|PSG4 Exp number of AAs in TMHs: 0.17792
# ENST00000599371|ENST00000599371.1|M0R2U8|PSG4 Exp number, first 60 AAs:  0.16566
# ENST00000599371|ENST00000599371.1|M0R2U8|PSG4 Total prob of N-in:        0.20572
ENST00000599371|ENST00000599371.1|M0R2U8|PSG4   TMHMM1.0        outside      1    96
```

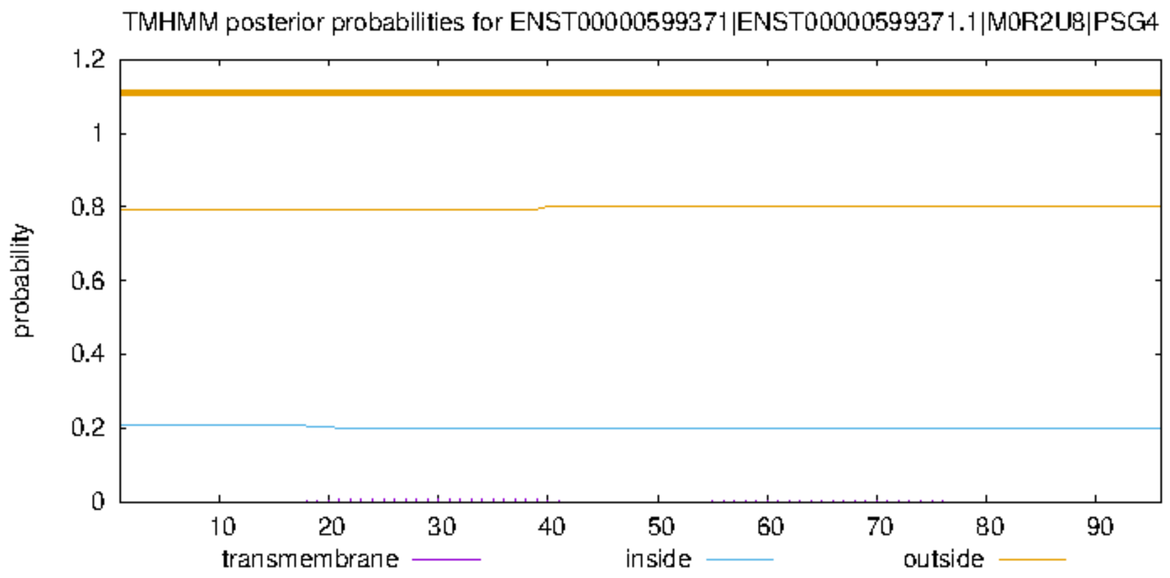

plot in postscript, script for making the plot in gnuplot, data for plot

---

```
# ENST00000595949|ENST00000595949.1|M0R215|PSG4 Length: 40
# ENST00000595949|ENST00000595949.1|M0R215|PSG4 Number of predicted TMHs:  0
# ENST00000595949|ENST00000595949.1|M0R215|PSG4 Exp number of AAs in TMHs: 0.02232
# ENST00000595949|ENST00000595949.1|M0R215|PSG4 Exp number, first 60 AAs:  0.02232
# ENST00000595949|ENST00000595949.1|M0R215|PSG4 Total prob of N-in:        0.10516
ENST00000595949|ENST00000595949.1|M0R215|PSG4   TMHMM1.0        outside      1    40
```

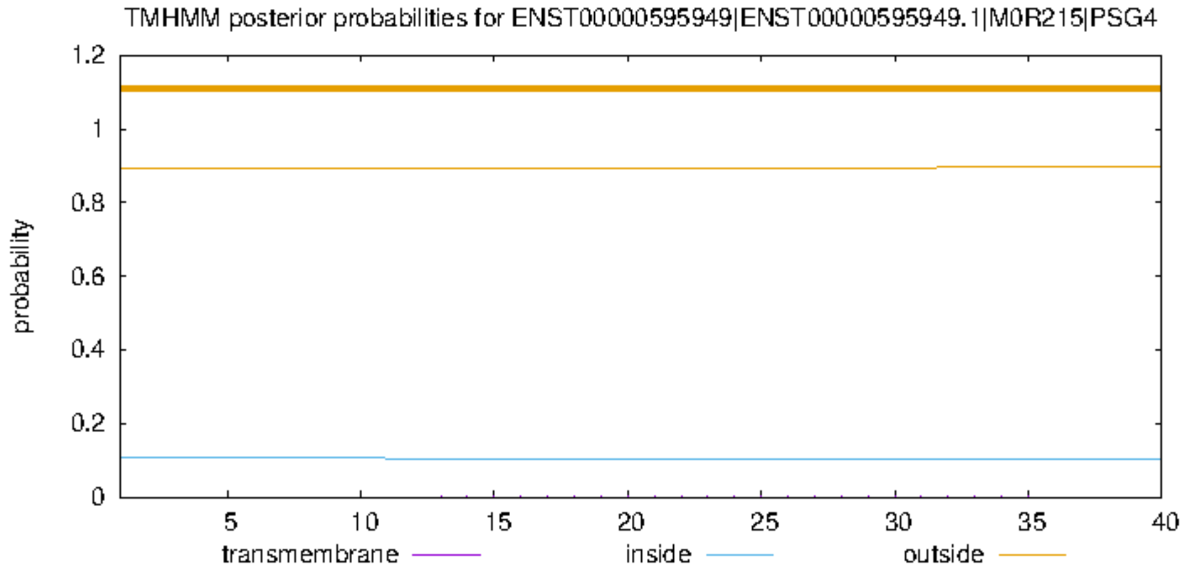

plot in postscript, script for making the plot in gnuplot, data for plot

---

```
# ENST00000601041|ENST00000601041.1|M0R046|PSG4 Length: 52
# ENST00000601041|ENST00000601041.1|M0R046|PSG4 Number of predicted TMHs:  0
# ENST00000601041|ENST00000601041.1|M0R046|PSG4 Exp number of AAs in TMHs: 0.06846
# ENST00000601041|ENST00000601041.1|M0R046|PSG4 Exp number, first 60 AAs:  0.06846
# ENST00000601041|ENST00000601041.1|M0R046|PSG4 Total prob of N-in:        0.11609
ENST00000601041|ENST00000601041.1|M0R046|PSG4   TMHMM1.0        outside      1    52
```

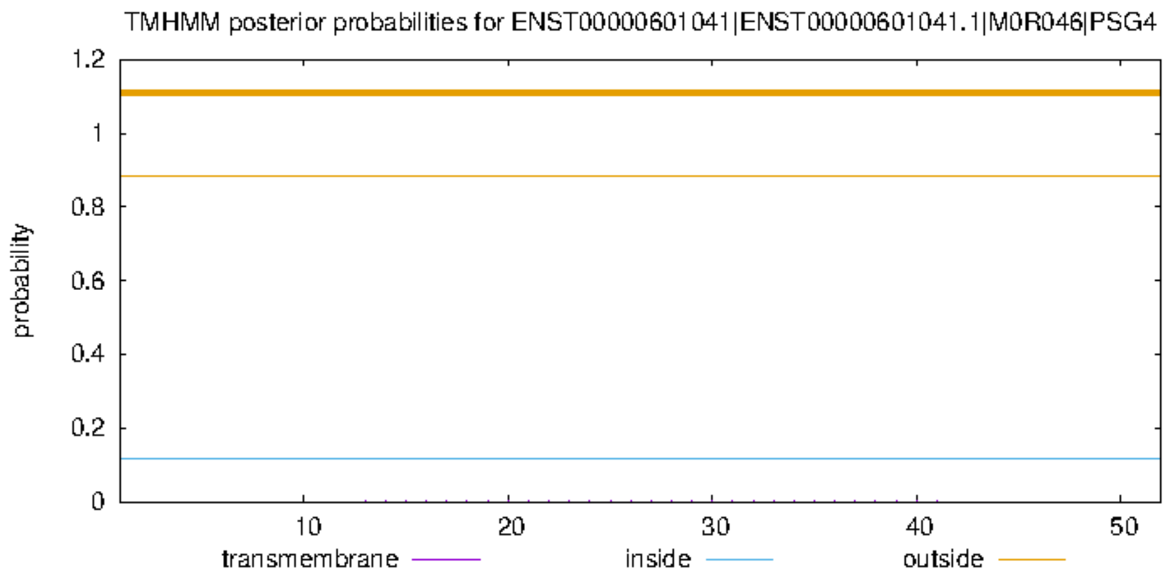

plot in postscript, script for making the plot in gnuplot, data for plot

---

```
# ENST00000599391|ENST00000599391.1|M0R2Y7|PSG4 Length: 180
# ENST00000599391|ENST00000599391.1|M0R2Y7|PSG4 Number of predicted TMHs:  0
# ENST00000599391|ENST00000599391.1|M0R2Y7|PSG4 Exp number of AAs in TMHs: 0.00741999999999999
# ENST00000599391|ENST00000599391.1|M0R2Y7|PSG4 Exp number, first 60 AAs:  0.00526
# ENST00000599391|ENST00000599391.1|M0R2Y7|PSG4 Total prob of N-in:        0.02145
ENST00000599391|ENST00000599391.1|M0R2Y7|PSG4   TMHMM1.0        outside      1   180
```

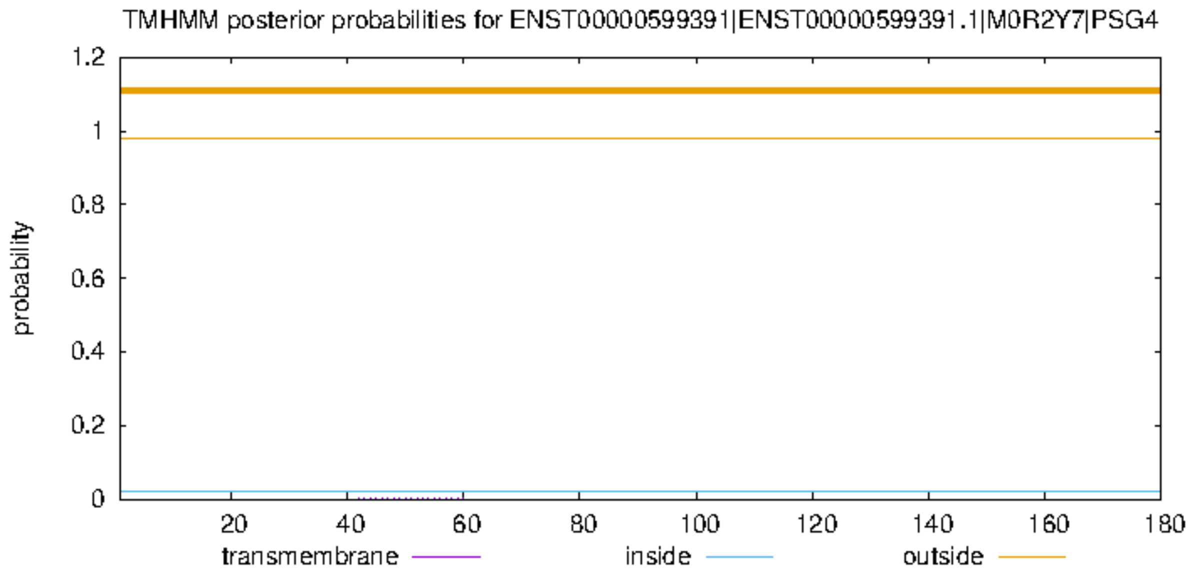

plot in postscript, script for making the plot in gnuplot, data for plot

---

```
# ENST00000595140|ENST00000595140.1|M0QX68|PSG3 Length: 475
# ENST00000595140|ENST00000595140.1|M0QX68|PSG3 Number of predicted TMHs:  0
# ENST00000595140|ENST00000595140.1|M0QX68|PSG3 Exp number of AAs in TMHs: 1.60511
# ENST00000595140|ENST00000595140.1|M0QX68|PSG3 Exp number, first 60 AAs:  1.29198
# ENST00000595140|ENST00000595140.1|M0QX68|PSG3 Total prob of N-in:        0.05820
ENST00000595140|ENST00000595140.1|M0QX68|PSG3   TMHMM1.0        outside      1   475
```

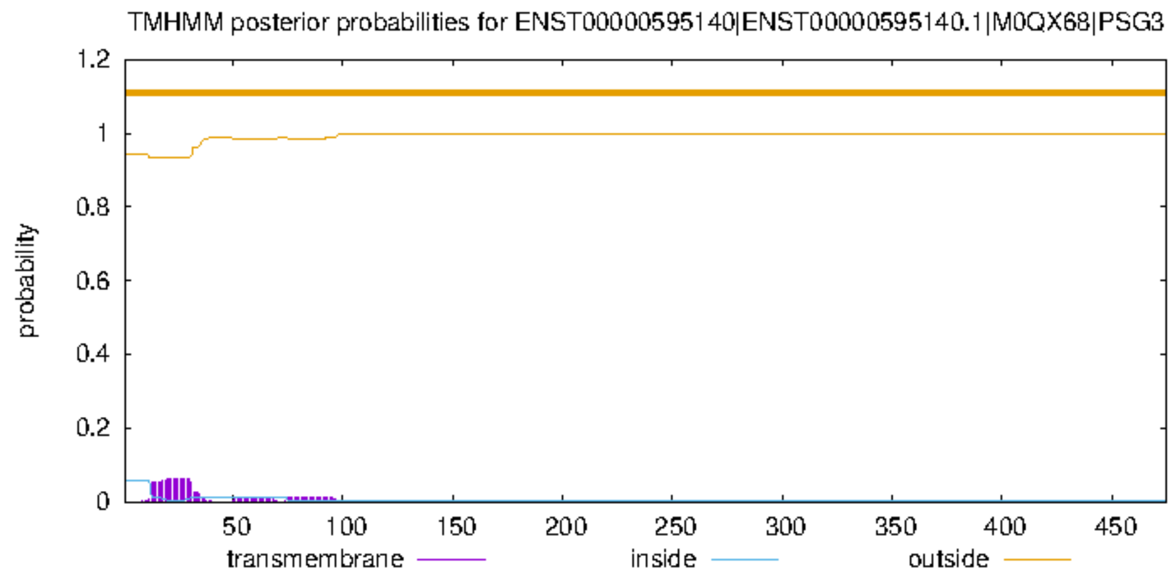

plot in postscript, script for making the plot in gnuplot, data for plot

---

```
# ENST00000623675|ENST00000623675.1|A0A096LNM5|PSG7 Length: 297
# ENST00000623675|ENST00000623675.1|A0A096LNM5|PSG7 Number of predicted TMHs:  0
# ENST00000623675|ENST00000623675.1|A0A096LNM5|PSG7 Exp number of AAs in TMHs: 0.000670000000000001
# ENST00000623675|ENST00000623675.1|A0A096LNM5|PSG7 Exp number, first 60 AAs:  5e-05
# ENST00000623675|ENST00000623675.1|A0A096LNM5|PSG7 Total prob of N-in:        0.00451
ENST00000623675|ENST00000623675.1|A0A096LNM5|PSG7       TMHMM1.0        outside      1   297
```

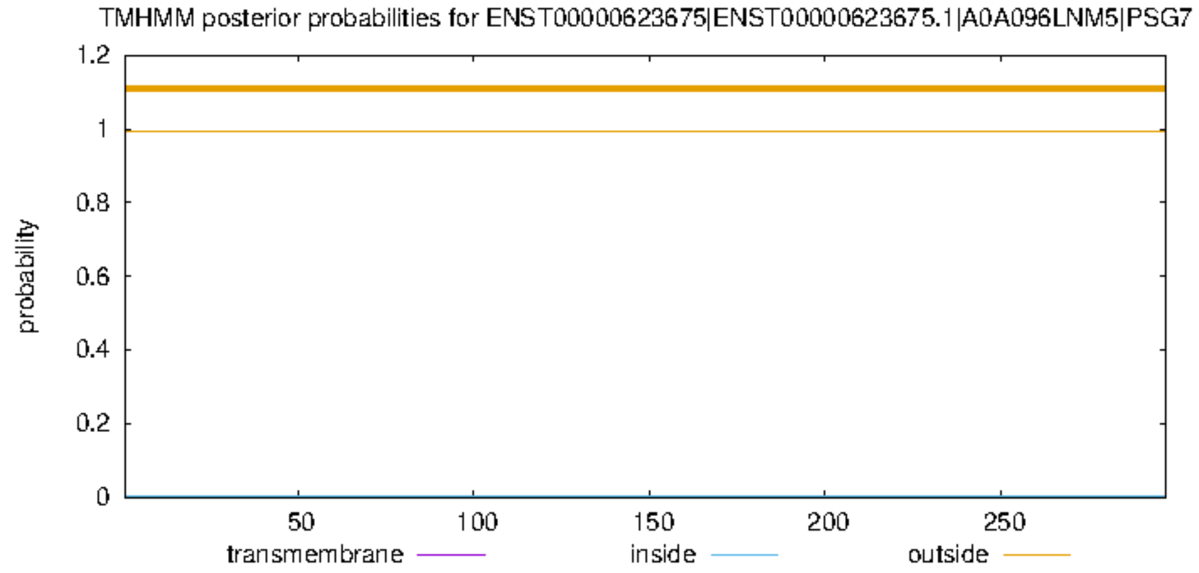

plot in postscript, script for making the plot in gnuplot, data for plot

---
