# Supplementary material for: Endogenous retroviral elements LTR8B and MER65 rewire PSG9 regulation to control trophoblast syncytialization and pre-eclampsia risk
Source: Genome Biol. 2026 Mar 9;27:73. doi: 10.1186/s13059-026-03944-z (PMC12969887; doi:10.1186/s13059-026-03944-z)
Supplement: Supplementary file 7 — Additional file 7. Analysis of PSG9 overexpression in SGHPL-4 cells. [file 13059_2026_3944_MOESM7_ESM.pdf]

edgeR_results

			logFC	logCPM	PValue	FDR
**ENSG00000196826**	ENSG00000196826	AC008758.1	8.23446174603821	-0.575230218583073	2.23E-16	7.63E-12
**ENSG00000183668**	ENSG00000183668	PSG9	7.72355106903139	12.3183094473509	2.25E-11	3.86E-07
**ENSG00000196358**	ENSG00000196358	NTNG2	-2.55262709267987	0.374665454813171	8.69E-09	9.91E-05
**ENSG00000170848**	ENSG00000170848	PSG6	2.70264374154154	-0.444215414897694	2.02E-07	0.001726638284909
**ENSG00000164627**	ENSG00000164627	KIF6	3.61965244969859	-1.33018454437332	2.77E-07	0.001899362442468
**ENSG00000155660**	ENSG00000155660	PDIA4	0.905579118468568	8.46062013398116	1.26E-06	0.007204651409616
**ENSG00000269900**	ENSG00000269900	RMRP	-6.58561287986905	-1.97033733460212	4.96E-06	0.021888310079383
**ENSG00000233791**	ENSG00000233791	LINC01136	-4.26799394487791	-2.13747310631701	5.12E-06	0.021888310079383
**ENSG00000277027**	ENSG00000277027	RMRP	9.75226245481436	0.848809351582347	8.38E-06	0.031039478204646
**ENSG00000123500**	ENSG00000123500	COL10A1	2.73016737435964	-0.377455010054374	9.07E-06	0.031039478204646

networkanalyst_enrichment_1

Pathway	Total	Expected	Hits	P.Value	FDR
**Assembly of collagen fibrils and other multimeric structures**	54	0.0162	1	0.0161	1
**Degradation of collagen**	61	0.0183	1	0.0182	1
**Collagen biosynthesis and modifying enzymes**	62	0.0186	1	0.0185	1
**Interferon alpha/beta signaling**	68	0.0204	1	0.0203	1
**Degradation of the extracellular matrix**	77	0.0231	1	0.0229	1
**Collagen formation**	85	0.0255	1	0.0253	1
**Extracellular matrix organization**	157	47	1	0.0465	1
**Interferon Signaling**	173	0.0518	1	0.0512	1
**Cytokine Signaling in Immune system**	286	0.0857	1	0.0838	1
**Immune System**	1140	341	1	312	1

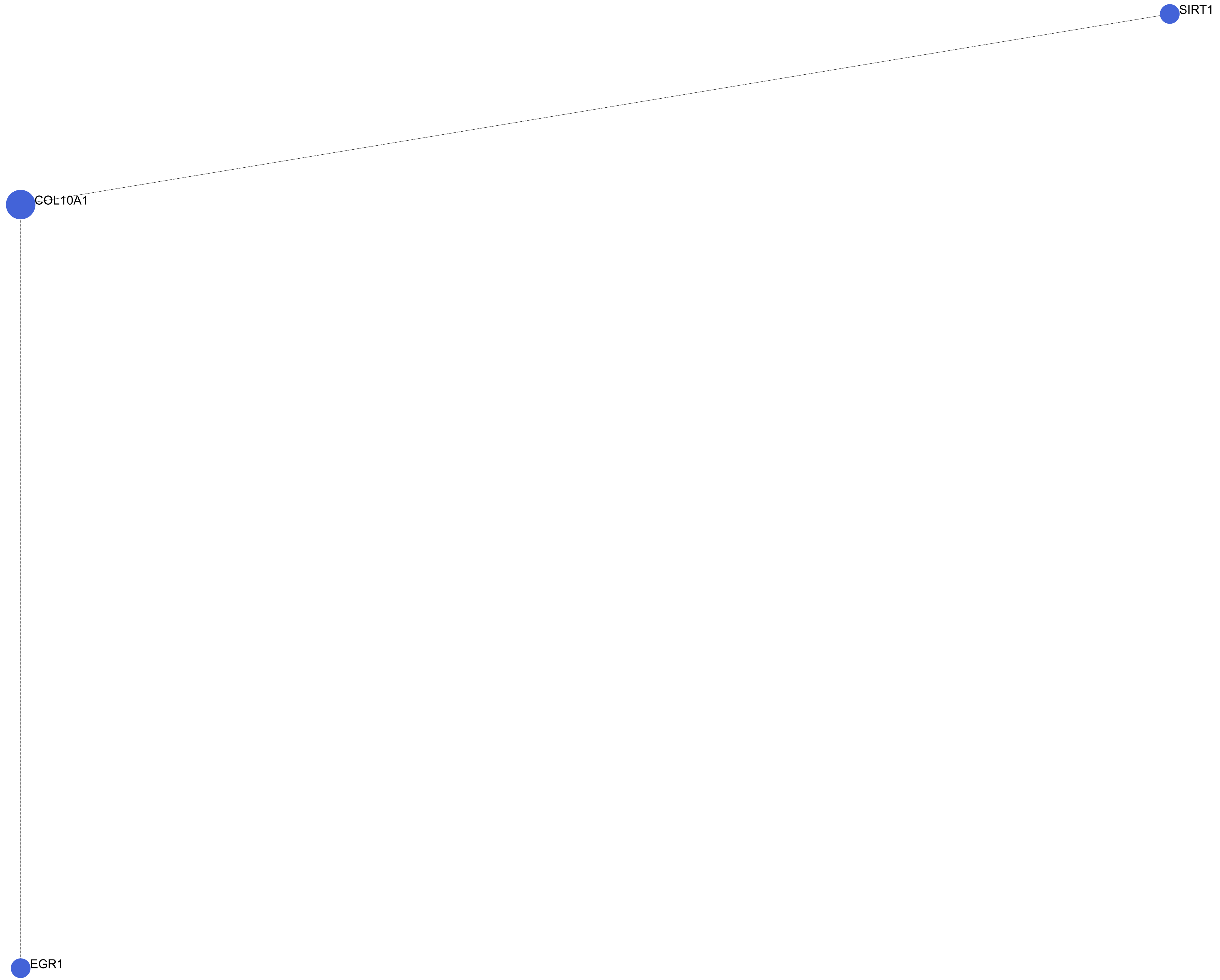
SIRT1
COL10A1
EGR1
